# Modulation of simultaneously collected hemodynamic and electrophysiological functional connectivity by ketamine and midazolam

**DOI:** 10.1002/hbm.24889

**Published:** 2019-12-06

**Authors:** Anna Forsyth, Rebecca McMillan, Doug Campbell, Gemma Malpas, Elizabeth Maxwell, Jamie Sleigh, Juergen Dukart, Jörg Hipp, Suresh D. Muthukumaraswamy

**Affiliations:** ^1^ School of Pharmacy, Faculty of Medical and Health Sciences The University of Auckland Auckland New Zealand; ^2^ Department of Anaesthesiology Auckland District Health Board Auckland New Zealand; ^3^ Department of Anaesthesiology Faculty of Medical and Health Sciences The University of Auckland Auckland New Zealand; ^4^ Institute of Neuroscience and Medicine, Brain & Behaviour (INM‐7) Research Centre Jülich Jülich Germany; ^5^ Institute of Systems Neuroscience, Medical Faculty Heinrich Heine University Düsseldorf Düsseldorf Germany; ^6^ Roche Pharma Research and Early Development, Roche Innovation Center Basel F. Hoffmann‐La Roche Ltd. Basel Switzerland

**Keywords:** electroencephalography, functional connectivity, functional magnetic resonance imaging, ketamine, midazolam, simultaneous EEG/FMRI

## Abstract

The pharmacological modulation of functional connectivity in the brain may underlie therapeutic efficacy for several neurological and psychiatric disorders. Functional magnetic resonance imaging (fMRI) provides a noninvasive method of assessing this modulation, however, the indirect nature of the blood‐oxygen level dependent signal restricts the discrimination of neural from physiological contributions. Here we followed two approaches to assess the validity of fMRI functional connectivity in developing drug biomarkers, using simultaneous electroencephalography (EEG)/fMRI in a placebo‐controlled, three‐way crossover design with ketamine and midazolam. First, we compared seven different preprocessing pipelines to determine their impact on the connectivity of common resting‐state networks. Independent components analysis (ICA)‐denoising resulted in stronger reductions in connectivity after ketamine, and weaker increases after midazolam, than pipelines employing physiological noise modelling or averaged signals from cerebrospinal fluid or white matter. This suggests that pipeline decisions should reflect a drug's unique noise structure, and if this is unknown then accepting possible signal loss when choosing extensive ICA denoising pipelines could engender more confidence in the remaining results. We then compared the temporal correlation structure of fMRI to that derived from two connectivity metrics of EEG, which provides a direct measure of neural activity. While electrophysiological estimates based on the power envelope were more closely aligned to BOLD signal connectivity than those based on phase consistency, no significant relationship between the change in electrophysiological and hemodynamic correlation structures was found, implying caution should be used when making cross‐modal comparisons of pharmacologically‐modulated functional connectivity.

## 1 INTRODUCTION

Functional integration in the brain requires coordinated information flow within and between neuronal networks (Bastos & Schoffelen, 2016), with this flow likely to be driven by synchronized rhythmic fluctuations in the activity of neuronal ensembles (Buzsáki & Wang, 2012; Siegel, Donner, & Engel, 2012; Singer, 1999; Womelsdorf et al., 2007). These fluctuations are thought to be reflected in the blood‐oxygen level dependent (BOLD) signal (Logothetis, 2003; Magri, Schridde, Murayama, Panzeri, & Logothetis, 2012), and functional connectivity analyses of fMRI data (fcFMRI) have confirmed the existence of spatially distinct fluctuations working in synchrony, now termed resting‐state networks (RSNs) (De Luca, Beckmann, De Stefano, Matthews, & Smith, 2006). These networks have been linked to various cognitive states (Fox et al., 2005; van den Heuvel & Hulshoff Pol, 2010), as well as several neurological and psychiatric disorders (M. Greicius, 2008; Rombouts, Barkhof, Goekoop, Stam, & Scheltens, 2005; Woodward, Rogers, & Heckers, 2011), and pharmacological modulations of these networks posited as potentially relevant to therapeutic action (Cole et al., 2013; Kelly et al., 2009; McCabe & Mishor, 2011). However, assessing pharmacological modulation of the BOLD signal is limited by the indirect nature of this measure. Drugs can affect neurotransmitters involved in signaling to the blood vessels controlling cerebral blood flow (CBF), vascular tone, and other properties of blood vessels and tissues, all of which could alter the neurovascular coupling characteristics that give rise to the BOLD signal and related connectivity metrics (Iannetti & Wise, 2007). As such, BOLD fMRI connectivity can provide a window into pharmacological modulation of neural communications, however, methodological challenges related to disentangling neural from non‐neural (physiological and artefactual) contributions to the signal remain.

Here we assessed fMRI connectivity in a drug study using two common connectivity methods; dual regression of RSNs derived from independent components analysis (ICA), and whole‐brain, all‐to‐all node‐based connectivity. In the first, we assessed the impact of different fMRI preprocessing pipelines on pharmacologically‐modulated RSNs. There is extensive literature regarding the impact and cleaning of noise in the BOLD signal (for reviews see: [Birn, 2012; Caballero‐Gaudes & Reynolds, 2017; Iacovella & Hasson, 2011; Murphy, Birn, & Bandettini, 2013]), with some researchers directly assessing the effect of various cleaning methodologies on datasets (Gavrilescu et al., 2008; Weissenbacher et al., 2009), however, there have been few articles evaluating the impact of these methods in pharmacological studies, and these are limited to one type of noise per article, for example, motion (Hlinka, Alexakis, Hardman, Siddiqui, & Auer, 2010), or using signals from physiological monitoring (Khalili‐Mahani et al., 2013). The current study assesses several types of noise removal on the same dataset, including CSF and WM regression, modelling physiological monitoring signals, and regressing noise components from spatially discrete sources, derived from ICA.

Each fMRI RSN has been associated with several EEG frequency bands (Mantini, Perrucci, Gratta, Romani, & Corbetta, 2007), making comparisons of the full components between modalities difficult. Therefore, in the second analysis we used a different connectivity metric; we compared the drug modulations of all‐to‐all connectivity matrices derived from the different fMRI preprocessing pipelines to those from two different measures of electrophysiological connectivity. We used two drugs that have been extensively researched in both EEG and fMRI studies and provide complementary insights into the effects of excitation and inhibition in the brain – ketamine and midazolam. At subanaesthetic concentrations, ketamine is primarily a nonselective antagonist of the glutamate *N*‐methyl‐d‐aspartate (NMDA) receptor (Krystal et al., 1994). Midazolam is a short‐acting sedating benzodiazepine, which performs as a positive allosteric modulator of the γ‐aminobutyric acid (GABA) A receptor (Michaloudis et al., 1998; Reinsel et al., 2000). The literature displays several commonalities in fMRI‐derived connectivity metrics, such as increased connectivity between the prefrontal cortex and subcortical areas with ketamine (Anticevic et al., 2015; Dandash et al., 2015; Grimm et al., 2015), and disruption of higher cognitive networks and increased connectivity within low‐level sensory networks with midazolam (M. Greicius, 2008; Kiviniemi et al., 2005; Liang et al., 2015). However, discrepancies between results for both drugs are also evident, especially with the default mode network (DMN), where ketamine has been shown to decrease connectivity (Bonhomme et al., 2016), increase connectivity (Fleming et al., 2019), or to have no effect (Mueller et al., 2018; Niesters et al., 2012), and midazolam to decrease (Liang et al., 2015), or have no effect (Greicius et al., 2008). While these discrepancies could be driven by differences in analysis, such as using different seed locations, they could also be due in part to different preprocessing strategies. Indeed, a recent study investigating the pharmacological modulation of neural activity (McMillan et al., 2019) showed that the commonly found deactivation of the subgenual anterior cingulate cortex by ketamine (De Simoni et al., 2013; Deakin et al., 2008; Doyle et al., 2013) disappeared after physiological noise correction. To assess the potential physiological confounds to the BOLD signal and its connectivity estimates, and to avoid comparison issues resulting from a priori seed location decisions, we used model‐free ICA combined with dual regression (Nickerson, Smith, Öngür, & Beckmann, 2017) to determine changes in six common RSNs, after seven different preprocessing strategies.

In the second part of this article, we compared pharmacologically‐modulated connectivity matrices derived from the different fMRI pipelines to those from two electrophysiological connectivity metrics. Unlike fMRI, EEG is a direct measure of neural activity, and has excellent temporal specificity, in the order of milliseconds (Laufs et al., 2003), but limited spatial resolution (Babiloni et al., 2004). In comparison, BOLD fMRI has excellent spatial specificity in the order of millimeters and can measure activity from deep cortical structures (Laufs et al., 2003), however it has poor temporal resolution (Iannetti & Wise, 2007). Recording these two modalities separately is limited by several factors that can vary from session to session including stability of drug response, spontaneous brain activity, and differences in the recording environment. Simultaneous EEG/fMRI has the potential to combine the spatiotemporal resolutions of these two modalities to provide more information than either alone, and to overcome pharmacological confounds of the BOLD signal. This technology has been used to compare temporal correlations of the BOLD signal with modulations of power in various EEG bands (Goldman, Stern, Engel, & Cohen, 2002; Mantini et al., 2007; Scheeringa et al., 2008), however, few studies have assessed the relationship between temporal correlations of the BOLD signal and commonly used methods of deriving electrophysiological connectivity (Deligianni, Centeno, Carmichael, & Clayden, 2014; Hipp & Siegel, 2015).

To our knowledge, this is the first attempt to assess the pharmacological modulations of the relationship between fMRI and EEG whole‐brain connectomes and builds on our previous work assessing the drug changes to power associations between these two modalities (Forsyth et al., 2018). To assess the relationship, whole‐brain all‐to‐all connectivity of the BOLD signal nodes was conducted and compared to electrophysiological connectivity estimates from the same nodes. Electrophysiological connectivity estimates were derived in two ways; using power envelopes (Hipp & Siegel, 2015): correlating time‐frequency power between nodes across time, and phase relations (Vinck, Oostenveld, van Wingerden, Battaglia, & Pennartz, 2011): assessing the distribution of phase angle differences between nodes, assuming that when neural populations are functionally coupled the timing (phase) of their oscillatory processes become synchronized.

In summary, the current study first assessed the impact of different preprocessing methods on ICA‐derived RSNs from the fMRI BOLD signal, to assess the influence of pharmacologically driven physiological noise. We then compared whole‐brain connectomes between the different fMRI pipelines and results from two different EEG connectivity methods. This analysis was intended to assess whether the modalities demonstrated similar changes in connectivity, or if data from both capture a more complete view; whether due to physiological confounds of the BOLD signal, or a difference in the captured temporal and spatial aspects of the neural activity.

## 2 METHODS

### 2.1 Participants and procedure

Thirty male participants (mean age 27.3 ± 6.2 years), physically and psychologically healthy, with an average body mass index of 24 (standard deviation [*SD*] = 3.5) were recruited via advertisements on campus fliers and University of Auckland websites, and screened for recreational drug use. The recruitment was limited to males due to the changes in GABA levels (Epperson et al., 2002) and EEG metrics (Sumner et al., 2018) across the menstrual cycle, which could confound a repeated measures design. Data from this study have previously been used to assess the sensitivity and direction of the spectral effects of each modality, and the temporal correlations between the BOLD signal and modulations of band‐limited EEG power (Forsyth et al., 2018), as well as the temporal dynamics of the pharmacological MRI response (McMillan et al., 2019). Participants were scanned on three separate occasions in a placebo‐controlled, three‐way cross‐over design using ketamine, midazolam, and placebo, where the participants were blinded to which drug they were receiving. Participants were randomized using a random‐number generator to one of six different condition‐order groups. Intravenous access was obtained via a cannula inserted into the antecubital fossa of the left arm. Drugs were administered to a subanaesthetic level through an intravenous line controlled by an infusion pump (Alaris PK, UK), programmed by a supervising anaesthesiologist, located in the MR control room. Racemic ketamine was administered with a 0.25 mg/kg bolus dose, followed by a 0.25 mg/kg/hr infusion. Doses were similar to those used in previous literature (Deakin et al., 2008; Muthukumaraswamy et al., 2015). Midazolam was administered with a 0.03 mg/kg bolus dose, followed by a 0.03 mg/kg/hr infusion, resulting in doses similar to prior studies (Greicius et al., 2008; Liang et al., 2015). Collection of plasma would have interfered with our scanning protocol, however, information regarding pharmacokinetics and plasma concentrations at similar doses can be found in the literature (for ketamine ~150 ng/ml (Clements and Nimmo, 1981) and for midazolam ~20 ng/ml (Platten et al., 1998)). Drug administration commenced 7 min into a 16‐min resting‐state scan. Participants were instructed to have their eyes open and fixated on a small cross on a projection screen. After the resting‐state scan, several tasks were performed, however, only resting‐state and structural scans will be presented here. There was a minimum of 48 hr between sessions to compensate for the washout periods of each of the drugs. The drugs were tolerated well, with only minimal and expected side effects, such as nausea and dizziness, in a small number (*n* = 6) of sessions.

### 2.2 Data acquisition

MR images were acquired on a 3 T MR scanner (Siemens Skyra, Erlangen, Germany) with a 20‐channel head coil. BOLD fMRI data were acquired using a T2*‐weighted echo planar imaging (EPI) sequence (TR 2200 ms, TE 27 ms, flip angle 79°, 30 interleaved 3 mm slices, voxel size 3 × 3 × 3 mm). For the resting‐state data reported here, 437 volumes were acquired (7 min predrug, 9 min postdrug). In each session, prior to the removal of the EEG cap and its attached contrast markers, a low‐resolution Magnetisation‐prepared Rapid Gradient‐Echo (MPRAGE) scan (TR 1900 ms, TE 3.21 ms, FOV 256 mm^2^, flip angle 9°, 96 2 mm slices, voxel size 1.3 × 1.3 × 2.0 mm) was acquired to capture electrode positions. In one of the three sessions for each participant, a high resolution MPRAGE scan was acquired after the EEG cap had been removed to avoid structural distortions (Klein et al., 2015; TR 2100 ms, TE 3.42 ms, flip angle 9°, 192 slices, voxel size 1 × 1 × 1 mm). A safety buzzer was placed in the right hand of the participant, foam wedges were used to stabilize the head, and typically several blankets used for participant comfort/warmth. Verbal contact was made with the participants between each scan to monitor any potential adverse events.

EEG data was recorded continuously using Brain Products (Brain Products GmbH, Germany; Gilching) equipment; two BrainAmp MR plus amplifiers with 64‐channel Braincaps. Electrode caps used the manufacturer standard layout with FCz as reference, AFz as ground and one drop‐down electrode attached to the participant's back to record the electrocardiogram (ECG). The amplifier system was placed on a sled behind the head coil within the scanner to reduce cable lengths. Data were recorded with a sampling rate of 5 kHz using BrainVision Recorder software, with BrainVision RecView used to check online data quality during MR acquisition. Filters of 0.1–250 Hz were used, and electrode impedances were below 10 kΩ prior to data acquisition. A SyncBox device (BrainProducts) was used to achieve synchronization between the EEG hardware and 10 MHz scanner clock. Before entering the scanner, individual electrode placements were recorded using an ultrasound digitization device (Zebris, Germany; Isny) for later source localization and co‐registration. Vitamin E capsules, used as contrast markers, were placed at electrode positions Cz, F5, CP5, and FC6.

During each scan participants wore a respiration belt, and a pulse oximeter plethsmyograph on the left index finger (Biopac, USA; Goleta, California). A nasal cannula was fitted to measure end‐tidal oxygen and carbon dioxide levels (ADInstruments, NZ; Dunedin). For safety monitoring, an additional pulse oximeter was placed on the middle finger of the left hand to measure heart rate and blood oxygen saturation (Nonin, USA; Plymouth, Minnesota). All these signals were recorded on a Biopac MP150 (CA) system. A blood‐pressure cuff was placed on participant's right arm for periodic (between scan) measurements of blood pressure.

### 2.3 EEG preprocessing

To remove the artefact caused by the fast switching of the MRI gradients, a variant of the standard template removal technique (Allen, Josephs, & Turner, 2000) was used, where the moving template is compensated for and reset based on obtained fMRI motion parameters (Moosmann et al., 2009). Data were low‐pass filtered (100 Hz cut‐off frequency) and down‐sampled to 500 Hz. The ballistocardiogram artefact caused by the pulsatile motion of blood in the head (Eichele, Moosmann, Wu, Gutberlet, & Debener, 2010) was removed using an automated method that combines ICA with singular value decomposition to remove and/or filter components from the data which share high levels of mutual information with the cardiac trace (Liu, de Zwart, van Gelderen, Kuo, & Duyn, 2012). The aforementioned steps were performed in EEGLAB (https://sccn.ucsd.edu/wiki/EEGLAB) with subsequent steps performed using a combination of custom scripts and Fieldtrip version 20160925 (Oostenveld, Fries, Maris, & Schoffelen, 2011) Subsequently, visual inspection of the raw EEG data allowed manual identification and removal of artefacts caused by head motion or jaw clenching. Average remaining data lengths in minutes (from the 7 min pre and postdrug blocks) were as follows: ketamine: predrug M = 6.63, standard error (*SE*) = .083, postdrug M = 6.31, *SE* = .15, midazolam: predrug M = 6.85, *SE* = .031, postdrug M = 6.59, *SE* = .093, placebo: predrug M = 6.63, *SE* = .076, postdrug = 6.59, *SE* = .067. Residual artefacts, such as those from eye blinks, were removed using ICA (number of components rejected: midazolam M = 7.67, *SE* = .31, ketamine M = 16.16, *SE* = .81, placebo M = 8.67, *SE* = .27) with components being manually identified through inspection of the temporal and spatial data.

### 2.4 fMRI preprocessing

fMRI analyses were carried out using the FMRIB Software Library (FSL) (Jenkinson, Beckmann, Behrens, Woolrich, & Smith, 2012). Seven different preprocessing pipelines were carried out to compare their effects on connectivity analyses. For all pipelines the following steps were applied: automated brain extraction using FSL's brain extraction tool (BET; Smith, 2002), motion correction using MCFLIRT (Jenkinson, Bannister, Brady, & Smith, 2002), spatial smoothing with a Gaussian kernel (5 mm FWHM), high‐pass temporal filtering at 0.01 Hz, registration to individual high‐resolution structural scans using FLIRT (Jenkinson et al., 2002), and registration to MNI standard brain images using FNIRT (Andersson, Jenkinson, & Smith, 2010). Further correction for motion and other physiological artefacts used FSL's FEAT for general linear modelling (GLM), with all models including 24 standard and extended motion parameters (Friston, Williams, Howard, Frackowiak, & Turner, 1996), and additional regressors differing between pipelines, as outlined below.

- *Simple preprocessing regressors (SIMPLE)*:

Average cerebrospinal fluid (CSF) and white matter (WM) signals, obtained using masks created by segmenting each individual's high‐resolution scan, and their temporal derivatives.

- *Physiological noise modelling (PNM) preprocessing regressors (PNM‐only)*:

Thirty‐four slice‐wise physiological regressors created using FSL's Physiological Noise Modelling (PNM) toolbox (Brooks et al., 2008), and two end‐tidal CO_2_ regressors. These included those for the cardiac and respiratory cycle (Glover, Li, & Ress, 2000), respiratory volume over time (Birn, Smith, Jones, & Bandettini, 2008), and heart rate (HR) (Chang, Cunningham, & Glover, 2009).

B. *Simple and PNM preprocessing regressors (SIMPLE + PNM)*:

CSF, WM, and PNM regressors.

C. *Independent components analysis (ICA) and PNM pre‐processing regressors (ICA + PNM)*:

Approximately 50 noise components per dataset derived from FSL's FIX (Griffanti et al., 2014; Salimi‐Khorshidi et al., 2014), plus the PNM regressors listed above.

D. *ICA regressors (ICA‐only)*

ICA regressors listed above.

E. *Simple and ICA preprocessing regressors (SIMPLE + ICA)*:

CSF, WM, and ICA regressors.

F. *Phase‐randomized preprocessing regressors (PHASERANDOMISED)*:

To assess whether the addition of regressors into the GLM resulted in a loss of signal as well as noise due to the reduced degrees of freedom (*df*), we created simulated PNM and ICA regressors via a technique created by Bright and Murphy (2015). Here the true noise regressors were repeatedly phase randomized until the temporal correlation with the true regressors was *r* < .1, and these were entered into the GLM instead of the true variables.

Data were split into 190 volumes predrug (7 min) and 190 volumes postdrug (7 min). Datasets exhibiting greater than 0.3 cm movement in at least 15% of their fMRI volumes, or those that had more than 20% of the EEG recording removed during visual inspection of the raw data, were removed from subsequent analyses. Consequently, all analyses were performed on 27 placebo, 25 midazolam, and 25 ketamine datasets (of which 23 had all three conditions from the same participants).

### 2.5 fMRI analysis

All participants' whole‐scan placebo data for all preprocessing pipelines were temporally concatenated together, and run through FSL's MELODIC 3.0 toolbox, which uses ICA to decompose 4D data sets into different spatial and temporal components (Beckmann & Smith, 2004). The number of ICs was estimated using the Laplace approximation to the Bayesian evidence for a probabilistic principal components model. These were visually examined and compared to the literature, before choosing six common resting‐state components (right and left Frontoparietal network (rFPN, lFPN), Sensory Motor Network (SMN), Visual Network (VN), posterior and anterior Default Mode Networks (pDMN, aDMN)) which were entered into a dual regression analysis using FSL (Nickerson et al., 2017). This analysis uses the ICA components derived from the pooled data as spatial regressors in a multiple regression onto each participant's 4D preprocessed data, which resulted, for each pipeline, in 27 participant‐specific time series per group‐level spatial component. These time series were variance‐normalized to allow for assessment of magnitude information and used as temporal regressors in a second multiple regression, again into each participant's 4D preprocessed data, to obtain participant‐ and condition‐specific spatial maps for each component. Subsequently, the differences between the most commonly used pipeline in the literature (SIMPLE) and all other pipelines were assessed at the group‐level with two‐tailed, paired‐sample *t*‐tests computed using FSL's randomize permutation‐testing tool (*p* < .05; Winkler, Ridgway, Webster, Smith, & Nichols, 2014). To correct for multiple comparisons, a threshold‐free cluster enhancement (TFCE) was used (Smith & Nichols, 2009). To assess whether ketamine or midazolam modulated the connectivity of these six networks, and if the changes were dependent on the preprocessing pipeline, ICA and dual regression were also performed on the data from the two drug sessions. Here, the resulting pre‐ and postdrug spatial maps for each of the 6 components from 25 participants in each drug condition were subjected to the same randomized paired‐sample *t* test as described above, and the analysis was repeated for each pipeline.

In order to quantify the differences in the modulations of RSN activity, participant‐level predrug parameter estimates (or those from the SIMPLE pipeline during placebo) were subtracted from postdrug parameter estimates (or those from the other pipelines during placebo), and then masked by the significant results from the randomization *t*‐tests. These were then masked by the component, and then by an inverse mask of the component, to assess the changes in connectivity both within the RSN and between the RSN and the rest of the brain. The parameter estimate values were summed, resulting in a value that represented both the strength of the connectivity change, and its spatial spread, both inside and outside the RSN, for each participant, for each condition. Subsequently, 6 (RSN) × 7 (Pipeline) repeated measures ANOVAs were performed for the ketamine and midazolam conditions, both within, and outside the RSNs, and 6 (RSN) × 6 (Pipeline) repeated measures ANOVAs were run for the placebo condition, again within and outside the RSNs. Posthoc *t*‐tests were performed between each pipeline across RSNs, and for each RSN separately, and Bonferroni corrected for multiple comparisons. It should be noted here that the decision to derive ICA and dual regression separately for each condition was based on the desire to explore each session‐type separately. There was no strong theoretical justification to directly compare the two drugs, and as spontaneous activity can vary from session to session (Birn et al., 2013), such a decision would have resulted in losing information specific to each session by combining across all three sessions.

To assess whole‐brain connectivity, 264 10 mm spherical *functionally defined* nodes (Power et al., 2011) were used. The averaged signal from within each node was used in an all‐to‐all Pearson's correlation analysis for pre‐ and postdrug for each of the preprocessing pipelines described above. The results were Fisher‐*Z* transformed, predrug subtracted from post, and one sample *t‐*tests to assess whether the group result was significantly different to zero, false discovery rate (FDR) corrected (*p* < .05).

### 2.6 EEG source localization and analysis

Individual electrode positions were co‐registered to the structural MRI of each participant. For source modelling, three‐shell boundary element models were constructed for each session using brain, skull, and scalp layers (Oostendorp & Oosterom, 1989). EEG data were average‐referenced and global covariance matrices were generated for the data, which were filtered into seven frequency bands: delta (1–4 Hz), theta (4–8 Hz), alpha (8–13 Hz), low beta (15–26 Hz), and high beta (28–40 Hz). These frequencies were chosen to avoid any potential slice artefact frequency and its harmonics (Ritter, Becker, Freyer, & Villringer, 2010). Linearly constrained minimum variance beamforming with 5% regularization (Veen, Drongelen, Yuchtman, & Suzuki, 1997) was applied at the 264 seed locations defined by Power et al. (2011) by reverse warping from the template head‐model provided with FieldTrip to generate spatial filters for each node. Data were split into trials equating to the TR in the fMRI data, and then sorted into those that fell within pre‐ and postdrug time frames.

Although there are myriad options to determine electrophysiological connectivity we selected two that were well defined in the literature, and capture relations between oscillatory neuronal activity using different data features: correlation of power and consistency of phases of band‐limited signals at two locations (Siegel et al., 2012; Wang, 2010). Electrophysiological connectivity measures based on the power envelopes allow assessment at similar time scales to the BOLD signal (Deligianni et al., 2014; Engel, Gerloff, Hilgetag, & Nolte, 2013), and are therefore thought to be more closely aligned with fMRI connectivity metrics than phase consistency measures. Here we test this prediction by comparing the two measures' connectivity metrices to those derived from the BOLD signal.

Power correlation: We quantified the correlation of band‐limited power at different locations, that is, the co‐variation of the amplitude oscillatory processes in different brain areas, using a method accounting for trivial correlation related to source leakage (see Hipp, Hawellek, Corbetta, Siegel, & Engel, 2012). This method discounts spurious correlation caused by limited spatial resolution by harnessing the fact that signal components from the same underlying source are characterized by an identical phase (Nolte et al., 2004). For each pair of signals the components sharing the same phase are removed before computing power estimates, essentially orthogonalising the signals before deriving power envelopes. Most human electrophysiological functional connectivity has been studied with magnetoencephalography (MEG) data (e.g., Brookes et al., 2011), and the methodology used in this article directly reflects that of Hipp et al. (2012), who demonstrated brain‐wide correlation of electrophysiological signals that were spatially highly structured and varied by frequency. Subsequently, we extracted Pearson correlation coefficients between each pair of nodes. After Fisher‐*Z* transformation, the predrug values were subtracted from the postdrug for each participant, and one sample *t‐*tests were performed to assess whether the group result was significantly different to zero, corrected for multiple comparisons across the nodes using FDR correction (*p* < .05), and maximum thresholded at ±7.

Consistency of phases: We used the weighted phase‐lag index (wPLI; Vinck et al., 2011) to quantify the consistency of phases, that is, agreement on the shortest temporal scale between band‐limited signals from different locations. The wPLI weights the contribution of the observed phase leads and lags by the magnitude of the Imaginary Component of the cross‐spectrum. This was calculated in trials of 2.2 s (equivalent to the TR of the fMRI data) using the FieldTrip toolbox. As with the orthogonalised data, results were Fisher‐*Z* transformed, predrug subtracted from post, averaged across the group using one‐sampled *t*‐tests, and FDR corrected.

### 2.7 Comparing whole‐brain connectomes from EEG and fMRI

**Figure 1:**
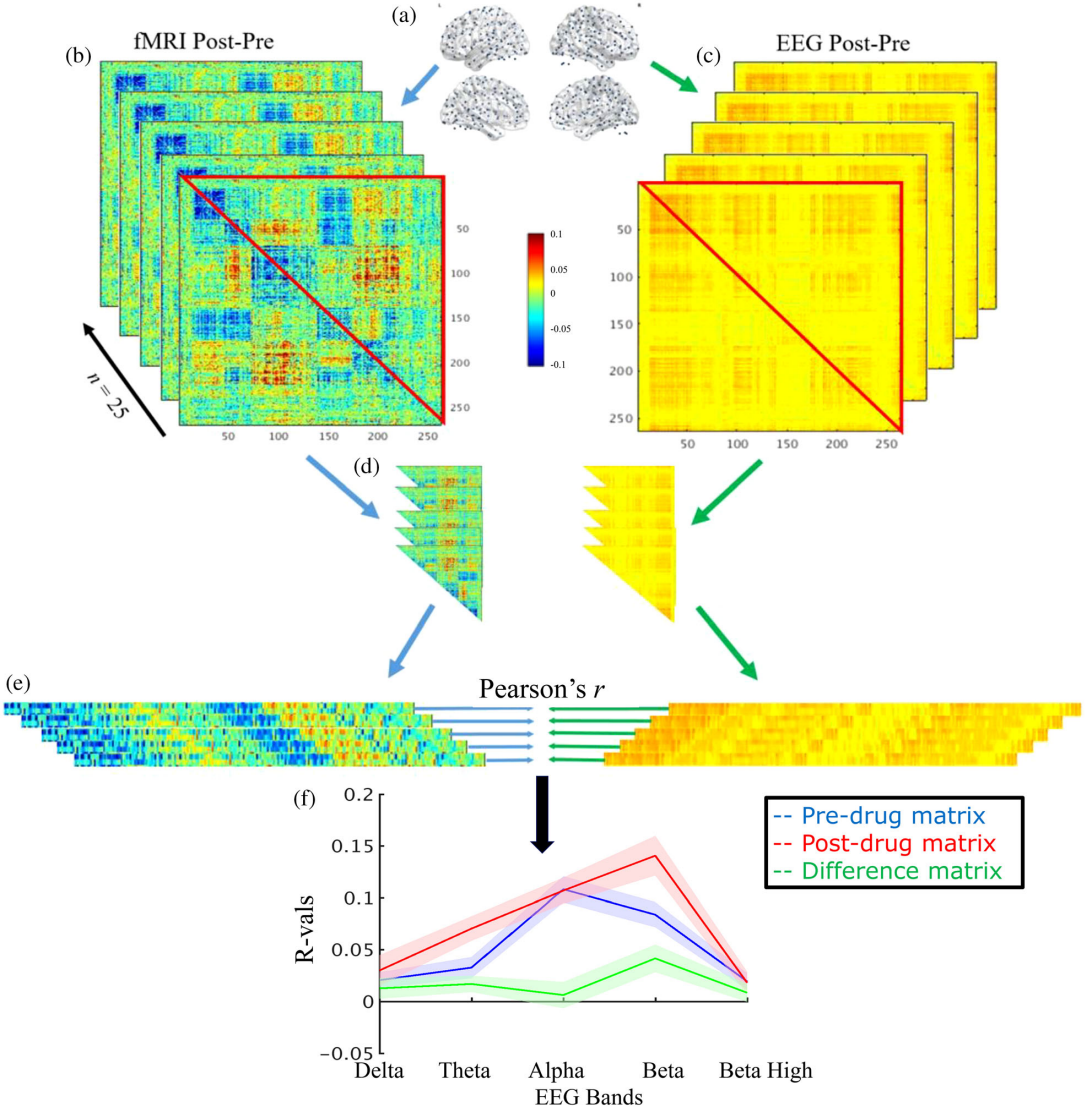
Comparing whole‐brain connectomes from EEG and fMRI: an illustration of the analysis workflow. (a) Connectivity analyses were performed in all‐to‐all fashion on 264 nodes prior to, and after drug administration for both fMRI (b) and EEG (c). Predrug matrices were subtracted from post‐drug, and the upper triangles (d) of the matrices were turned into vectors (e). These EEG and fMRI vectors were submitted to a correlation analysis, for each participant. Correlation values were Fisher *Z*‐transformed and averaged, and this was repeated for pre‐ and post‐drug separately, and for two different fMRI pre‐processing pipelines, and all five EEG bands (f)

For both variants of electrophysiological connectivity, and for two of the fMRI pipelines, the upper triangular part of the Fisher‐*Z* transformed Pre, Post, and difference (post‐pre) correlation matrices were vectorised, (see Figure 1 for schematic). The two fMRI pipelines were chosen to compare data cleaning between the two pipelines that showed the most difference during the dual regression analysis (SIMPLE and ICA‐only). A Pearson's correlation analysis between each EEG band and the two fMRI pipelines was run. One‐sample *t*‐tests were performed on the correlations to assess if they were significantly different from zero (*p* < .05 Bonferroni corrected for 30 tests run on each fMRI preprocessing pipeline: the five EEG bands, two electrophysiological connectivity metrics (power correlations, coherence), three conditions (predrug, postdrug, and post‐pre drug)). The coordinates and anatomical labels, defined by the Harvard‐Oxford Cortical and Subcortical Atlases, of the nodes involved with the 10 strongest modulations for each dataset can be found in Tables S1–S6.

## 3 RESULTS

### 3.1 Exploring differences in the noise structures between conditions

**Figure 2:**
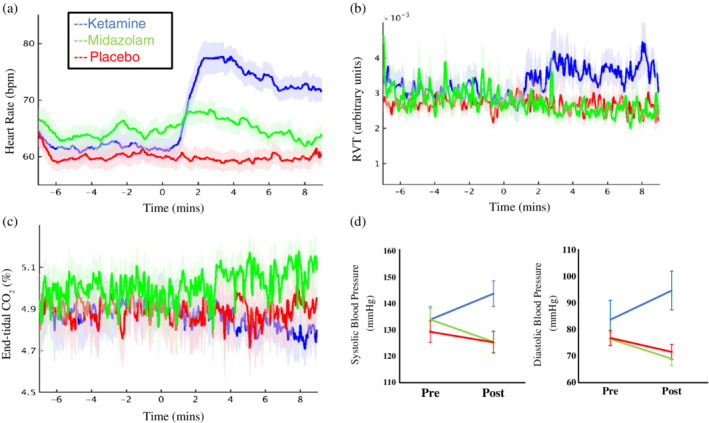
Changes in physiological measures. Depicted are the changes across time in heart rate (a), respiratory volume over time (b), and end‐tidal CO_2_, and the change in blood pressure between pre‐ and post‐drug, for placebo (red), ketamine (blue), and midazolam (green). Infusion started at time 0, and error bars/shading are the standard error of the mean. Only ketamine significantly modulated the first three parameters, with increases in HR (*p* < .001) and RVT (*p* = .04), and decreases in end‐tidal CO_2_ (*p* = .003). Ketamine also significantly increased both systolic (*p* = .01) and diastolic (*p* = .002) blood pressure, while midazolam decreased these (systolic: *p* < .001, diastolic: *p* = .005)

Figure 2 shows the group‐level changes over time of HR, blood pressure, RVT, and end‐tidal CO_2_. When looking at each condition separately, comparing pre‐ and postdrug, only ketamine had significant changes in HR (mΔ = 13.52 bpm, *SE* = 2.41, *p* < .001), end‐tidal CO_2_ (mΔ = −.15%, *SE* = .43, *p* = .003), and RVT (mΔ = 7.43e^−4^, *SE* = 3.31, *p* = .04). Ketamine also significantly increased systolic (mΔ = 9.86 mmHg, = = 3.27, *p* = .01) and diastolic (mΔ = 11 mmHg, SE = 2.74, *p* = .002) blood pressure, while midazolam significantly decreased these (systolic: mΔ = −8.429 mmHg, SE = 1.63, *p* < .001, diastolic: mΔ = −7.43 mmHg, SE = 2.23, *p* = .005). However, ketamine's impact on both systolic (*F*_[2,26]_ = 10.74, *p* < .001) and diastolic (*F*_[2,26]_ = 12.75, *p <* .001) blood pressure were significantly greater than midazolam's.

### 3.2 fMRI ICA and dual regression

In the first part of this article, we investigated the impact of six preprocessing approaches on the quantification of baseline and drug‐related changes in BOLD resting‐state functional connectivity as assessed with ICA. We started by comparing differences in the connectivity of RSNs resulting from a commonly used, simple preprocessing pipeline and those from six more sophisticated pipelines for placebo. We then assessed the pharmacological modulations of the RSNs by ketamine and midazolam, and how these modulations differ between preprocessing pipelines.

### 3.3 Placebo

Dual regression was used to assess the differences in connectivity of RSNs derived from the first 7 min of placebo resting‐state data for the commonly used preprocessing pipeline (SIMPLE) and six more sophisticated preprocessing pipelines for six commonly reported RSNs: the right and left frontoparietal network (lFPN, rFPN), the sensory motor network (SMN), the visual network (VN), and the posterior and anterior default mode networks (pDMN, aDMN).

**Figure 3:**
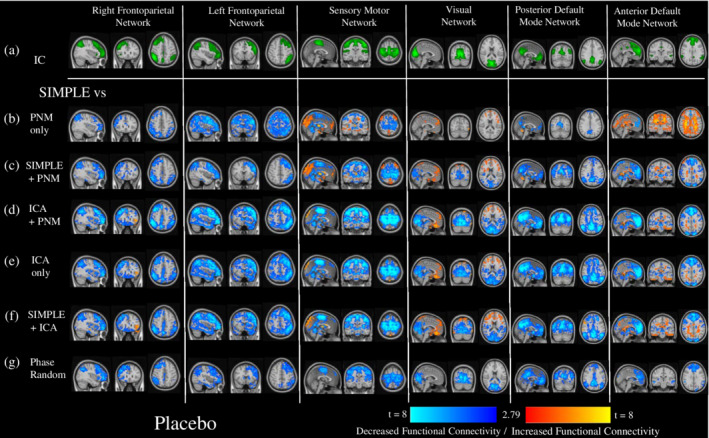
The impact of different preprocessing pipelines on the connectivity of six common resting‐state networks. This figure depicts the differences in connectivity of six RSNs derived from independent components (depicted in green, Row a.)) for six different preprocessing pipelines compared to the most frequently used preprocessing pipelines, using the first 7 min of placebo data. Depicted are t‐value maps from a dual regression between the simplest pipeline (CSF and WM regressors only), and all other pipelines, which are masked by areas that demonstrated significant change from a randomized paired‐*t* test, with a Bonferroni‐corrected threshold of *p* < 0.05. (b) PNM regressors only, (c) CSF, WM, and PNM regressors, (d) ICA‐derived and PNM regressors, (e) ICA derived regressors only, (f) CSF, WM, and ICA‐derived regressors, (g) CSF, WM, and the same number of regressors as in the most extensive pipeline (ICA + PNM), but phase‐randomized to ensure they are true noise. Increases in connectivity compared to the SIMPLE pipeline are shown in red‐yellow, decreases in dark‐light blue

The results of the dual regression analysis on the placebo data can be found in Figure 3. The pipelines using just the addition or replacement with PNM regressors (Figure 3b,c) showed areas of reduced connectivity within the networks compared to the SIMPLE pipeline, and increased connectivity between the networks and the rest of the brain, especially in the SMN, VN, and aDMN. The pipelines containing ICA (Figure 3d‐f) also showed primarily reduced connectivity, and while this was often limited to within network connectivity, it was more widespread than the changes with the phase‐randomized pipeline (Figure 3g), where connectivity changes were limited to reductions within the component areas. Some areas of increased connectivity between the VN and aDMN to other areas of the brain were found with the ICA‐denoised pipelines. Figure 6a depicts a bar plot of the summed changes in parameter estimates, both within the RSNs, and between them and the rest of the brain. The ICA‐pipelines (varying shades of blue) most commonly showed the largest decreases in connectivity compared to the SIMPLE pipeline, both within and outside the RSNs. Two 6 (RSN) × 6 (Pipeline) repeated measures ANOVAs were run on the within and outside RSN results, and, while both main effects and the interaction effect were significant, we focus on the main effect of pipeline for all ANOVAs run. For both within and outside the RSN, the Mauchley's test indicated that the assumption of sphericity had been violated (within: *χ* ^2^ = 515.34, *p <* .001, outside: *χ* ^2^ = 504.12, *p* < .001), therefore Greenhouse–Geisser corrected tests are reported (within: *ε* = .228, outside: *ε* = .223). The results show that there was a significant main effect of pipeline on RSN connectivity, with large effect sizes, both within: *F*_(1.1, 59.2)_ = 146.57, *p* < .001, *η* ^2^ = .734, and outside: *F*_(1.1, 60.4)_ = 232.15, *p* < .001, *η* ^2^ = .814. Posthoc *t*‐tests between pipelines (see Table S1a,b), across RSNs, show significant differences between all pipelines both within and outside RSNs. The average changes in connectivity to the SIMPLE pipeline, their SEs, across RSNs can be found in Table S1c,d). The vast majority of posthoc *t*‐test comparisons were statistically significant between pipelines for each RSN individually (see Table S1e,f). There were a few notable exceptions, mainly between pipelines containing PNM regression and the phase‐randomized pipeline and between pipelines containing ICA regressors, both within and outside RSNs.

The results of this analysis demonstrate that preprocessing choices can have substantial impact on the connectivity of resulting RSNs and highlight the importance of clearly detailing these choices. We next assessed the differences caused by the preprocessing pipelines on the pharmacological modulations of these RSNs, that is, the contrast of post‐ and preinfusion resting connectivity for ketamine and midazolam, again using dual regression.

### 3.4 Ketamine

**Figure 4:**
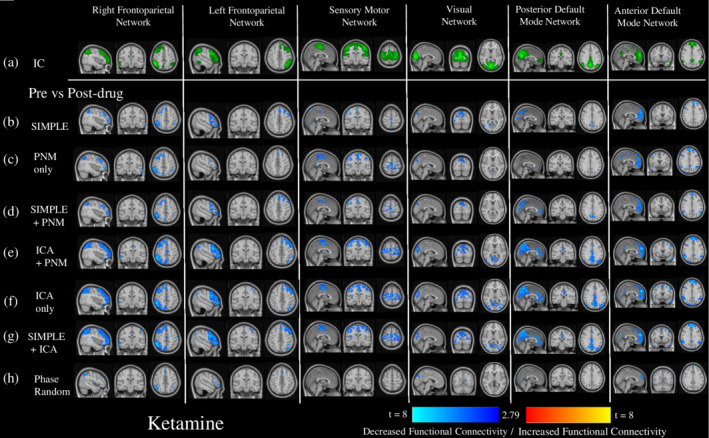
The impact of different preprocessing pipelines on the pharmacological modulation of six common resting‐state networks by ketamine. This figure depicts the changes in connectivity from baseline of six RSNs derived from independent components (depicted in green, Row a.)) after the administration of ketamine, for different preprocessing pipelines. Shown are *t*‐value maps from a dual regression between pre‐ and post‐drug, with increases in connectivity depicted in red‐yellow, and decreases in dark‐light blue, masked by areas that demonstrated significant change from a randomized paired‐*t* test, with a Bonferroni‐corrected threshold of *p* < .05. This was done after seven different preprocessing pipelines: (b) CSF and WM regressors only, (c) PNM regressors only, (d) CSF, WM, and PNM regressors, (e) ICA‐derived and PNM regressors, (f) ICA derived regressors only, (g) CSF, WM, and ICA‐derived regressors, (h) CSF, WM, and the same number of regressors as in the most extensive pipeline (ICA + PNM), but phase‐randomized to ensure they are true noise

Ketamine caused reduced connectivity in the RSNs, with spatial differences for each preprocessing pipeline (Figure 4). The pipelines containing only CSF and WM or PNM regression (Figure 4b‐d) generally depicted weaker and more spatially constricted reductions after drug administration than those utilizing ICA denoising (Figure 4e‐g). The phase‐randomized pipeline often reflected the simplest one, however the changes were spatially smaller, and disappeared altogether in the SMN (Figure 4h). ANOVAS run on data presented in Figure 6b (Greenhouse–Geisser corrected as: within: *χ* ^2^ = 183.73, *p <* .001, *ε* = .233, outside: *χ* ^2^ = 175.94, *p* < .001 *ε* = .311) showed a significant main effect on RSN connectivity, with large effect sizes, both within: *F*_(1.4, 33.5)_ = 103.70, *p* < .001, *η* ^2^ = .812, and outside: *F*_(1.9, 44.7)_ = 135.40, *p* < .001, *η* ^2^ = .849. Posthoc *t*‐tests between pipelines (see Table S2a,b), across RSNs, were mostly significantly different, with exceptions between some of the simpler pipelines and the ICA‐only and SIMPLE + ICA pipeline within RSNs, and between the ICA + PNM and PNM‐only pipelines outside RSNs. The average changes in connectivity after drug administration, and their SEs, across RSNs can be found in Table S2c,d). The vast majority of posthoc *t*‐test comparisons between pipelines for each RSN individually were statistically significant (see Table S2e,f). There were some exceptions, mainly between pipelines containing PNM regression and the phase‐randomized pipeline, between those containing PNM regression and the SIMPLE pipeline, between pipelines containing ICA regressors, and occasionally between ICA + PNM and the simpler pipelines, both within and outside RSNs.

### 3.5 Midazolam

**Figure 5:**
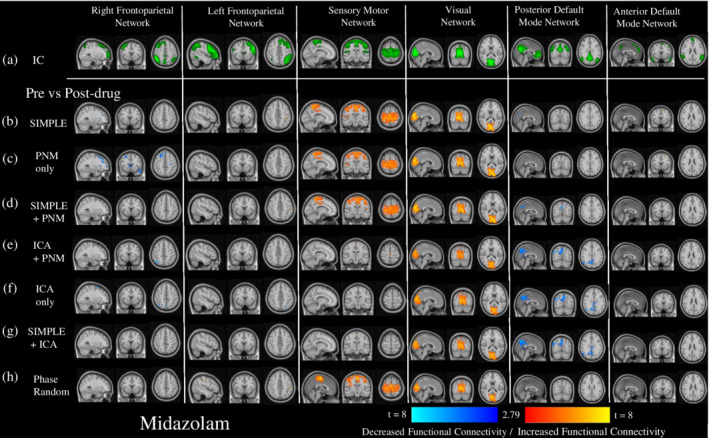
The impact of different preprocessing pipelines on the pharmacological modulation of six common resting‐state networks by midazolam. This figure depicts the changes in connectivity from baseline of six RSNs derived from independent components (depicted in green, Row a.)) after the administration of midazolam, for different preprocessing pipelines. Shown are *t*‐value maps from a dual regression between pre‐ and post‐drug, with increases in connectivity depicted in red‐yellow, and decreases in dark‐light blue, masked by areas that demonstrated significant change from a randomized paired‐*t* test, with a Bonferroni‐corrected threshold of *p* < .05. This was done after seven different preprocessing pipelines: (b) CSF and WM regressors only, (c) PNM regressors only, (d) CSF, WM, and PNM regressors, (e) ICA‐derived and PNM regressors, (f) ICA derived regressors only, (g) CSF, WM, and ICA‐derived regressors, (h) CSF, WM, and the same number of regressors as in the most extensive pipeline (ICA + PNM), but phase‐randomized to ensure they are true noise

**Figure 6:**
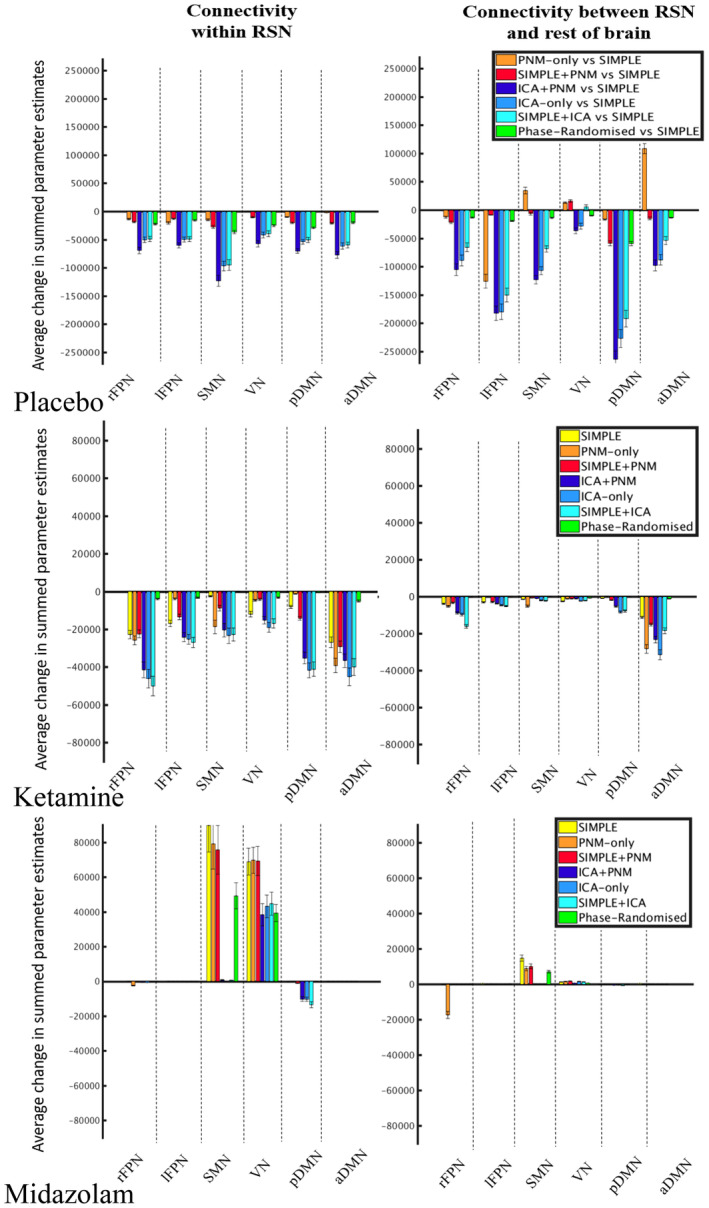
Average changes in connectivity within and outside RSNs for each preprocessing pipeline. This figure depicts the average change in summed parameter estimates from the results of the dual regressions on placebo (a), ketamine (b), and midazolam (c) data, for each RSN, and each preprocessing pipeline. NB: *y*‐axis scale is different for the two drug conditions compared to the placebo condition

In general, midazolam increased connectivity in sensory networks (SMN, VN), and decreased connectivity in some of the higher cognitive networks (rFPN, pDMN) (Figure 5). The pipelines containing only CSF and WM or PNM regression (Figure 5b–d) showed stronger and more wide‐spread increases in connectivity after drug administration in the SMN, similar changes in the VN, and fewer decreases in connectivity in the higher cognitive networks than the pipelines utilizing ICA derived regressors (Figure 5e–g). The phase‐randomized pipeline again most often reflected the simplest pipeline (Figure 5h). ANOVAS run on data presented in Figure 6c (Greenhouse–Geisser corrected as: within: *χ* ^2^ = 294.77, *p <* .001, *ε* = .187, outside: *χ* ^2^ = 298.53 *p* < .001, *ε* = .298) show that there was a significant main effect of pipeline on RSN connectivity, with large effect sizes, both within: *F*_(1.1, 27.0)_ = 32.41, *p* < .001, *η* ^2^ = .575, and outside: *F*_(1.8, 42.9)_ = 65.759, *p* < .001, *η* ^2^ = .733. Posthoc *t*‐tests between pipelines (see Table S3a,b), across RSNs, demonstrate primarily significant differences, with some exceptions within the simpler pipeline comparisons, and within those that used ICA regression. The average changes in connectivity after drug administration, the average differences between pipelines, and the respective SEs, across RSNs can be found in Table S3c,d). The vast majority of posthoc *t*‐test comparisons between pipelines for each RSN individually were statistically significant (see Table S3e,f). There were some exceptions, mainly between pipelines containing PNM regression and the phase‐randomized pipeline, between those containing PNM regression and the SIMPLE pipeline, between pipelines containing ICA regressors, and occasionally between ICA + PNM and the simpler pipelines, both within and outside RSNs.

The results from the first part of our article clearly demonstrate the effects of different preprocessing strategies on the results of an ICA and dual regression analysis to determine pharmacological modulation of RSNs. The overall pattern demonstrates similarities between the simpler pipelines (SIMPLE, PNM‐only, SIMPLE + PNM), occasionally between these and the phase‐randomized pipeline, and between those that used ICA regression. However, the lack of a ground truth makes discerning the most effective pipeline difficult, and all pipelines were brought forward to the all‐to‐all node connectivity analysis in the second part of the article.

### 3.6 Whole‐brain connectomes from EEG and fMRI

In the second part of the article, we compared fMRI connectivity to connectivity metrics derived from electrophysiological signals. We used another common method of assessing connectivity; the all‐to‐all relationship between the activity of brain‐wide nodes. We assessed the differences between pharmacologically modulated connectivity matrices of 264 nodes resulting from the different preprocessing pipelines from the first analysis and compared these to drug‐modulated matrices derived from electrophysiological measures of connectivity. As EEG is a direct measure of neural activity, comparing it to fMRI results may help clarify which preprocessing pipeline is closest to the ground truth. However, the relationship between the information that is used by the different methodologies in each modality is still unclear, and the electrophysiological metric that would best represent the information in correlations of the BOLD signal is yet to be determined. We utilized two EEG metrics, one based on correlations of the power envelope, and the other on phase relations between the nodes. Connectivity matrices were generally stable across the placebo scan, with only a few increases seen after the simpler preprocessing pipelines and in the delta power envelope when pre and post scan were compared (Figure S2).

### 3.7 Ketamine

**Figure 7:**
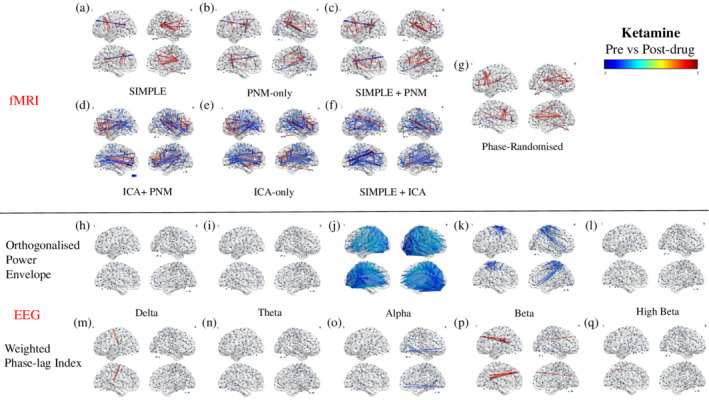
Modulation of all‐to‐all connectivity of 264 functional nodes after ketamine administration. Depicted are results from a FDR corrected (*p* < .05) one‐sample *t*‐test on the difference between post‐ and predrug connectivity estimates, increases depicted in red‐yellow, decreases in dark‐light blue. Connectivity values are derived through Pearson correlation of the BOLD signal for each preprocessing pipeline (a–g), and via either Pearson correlation of the orthogonalised power envelope (h–l), or the weighted phase‐lag index (m–q) for each EEG band

After preprocessing pipelines containing only containing only CSF and WM or PNM regression (Figure 7a–c), most of the significant changes in BOLD fMRI connectivity between the 264 nodes after ketamine administration were increases, with the strongest changes occurring between frontal (anterior cingulate cortex [ACC] and medial frontal gyrus [mFG]) and parietal (supramarginal gyrus [SMG] and precuneus), insular, and occipital (intracalcarine [ICC]) cortices. There was also at least one strong connectivity decrease between the frontal lobe and either the precuneus or the lateral occipital cortex (lOC) in each of these three pipelines (Table S4a–c). In comparison, far more changes were seen with the ICA‐denoising pipelines, and these were predominantly reduced connectivity with the strongest reductions occurring between the ACC and the insular and parietal (precuneus) cortices, between the paracingulate gyrus and the precuneus and occipital cortices, and within the sensory‐motor cortex (Figure 7d‐f, Table S4d–f). Some areas demonstrated increases in connectivity, predominantly in the ICA + PNM pipeline, with the strongest changes being between homologous areas of the prefrontal cortex (PFC), and between the ACC and the frontal pole (FP). The different pipelines had a range of 79–217 connections that significantly increased in connectivity, and for those that were present in all ICA‐denoised pipelines, 10 were also present in pipelines containing only CSF and WM or PNM regression, involving connections between the precuneus, operculum, and insular cortices to the lateral occipital cortex and frontal gryi. The phase‐randomized pipeline (Figure 7g, Table S4g) resulted in increases similar to the simplest pipeline.

Electrophysiological connectivity estimates derived from the power envelopes demonstrated reductions in connectivity in the alpha range (Figure 7j) within the occipital lobe, and between this lobe and frontal, parietal, and temporal areas. The strongest reductions all occurred in posterior areas of the brain (Table S5c). Reductions were also seen in the low beta range (Figure 7k, Table S5d), primarily within the sensory motor cortex (SMC); between pre‐ and postcentral gyri, and between their homologous areas, and also between this cortex and the precuneus and lOC.

Connectivity estimates derived from the wPLI showed far fewer connectivity changes. The delta band had one significant increase in connectivity, between the IC and the superior frontal gyrus (sFG; Figure 7m, Table S6a). The theta band demonstrated increases between the FP and primarily the IC areas (Figure 7n, Table S6b). The only band to show reduced connectivity was alpha, with the strongest changes occurring within the occipital and temporal cortices, and a small number between these posterior areas and the FP (Figure 7o, Table S6c). The low beta band demonstrated the strongest increases between the FP and posterior areas including the precuneus, cuneal cortex (CC), and insular cortex (Figure 7p, Table S6d). The high beta band showed only one significant change; an increase in the connectivity between the lOC and the sFG (Figure 7q, Table S6e).

### 3.8 Midazolam

**Figure 8:**
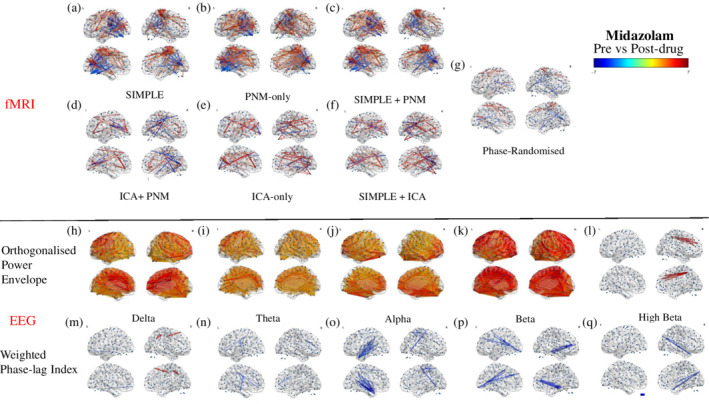
Modulation of all‐to‐all connectivity of 264 functional nodes after midazolam administration. Depicted are results from a FDR corrected (*p* < .05) one‐sample *t*‐test on the difference between post‐ and predrug connectivity estimates, increases depicted in red‐yellow, decreases in dark‐light blue. Connectivity values are derived through Pearson correlation of the BOLD signal for each preprocessing pipeline (a–g), and via either Pearson correlation of the orthogonalised power envelope (h–l), or the weighted phase‐lag index (m–q) for each EEG band

After midazolam administration, the strongest fMRI changes occurred in the pipelines containing only CSF and WM or PNM regression, with similar patterns occurring in all three; reductions radiating out from the occipital lobe, and spatially widespread increased connectivity (Figure 8a–c). The strongest reductions in all three included between the occipital lobe (ICC) and the SMC, with the SIMPLE + PNM also demonstrating reduced connectivity between the occipital and temporal lobes (Table S7a–c). The strongest increases were seen between the precuneus and other posterior areas (lingual gyrus (LG), CC, occipital pole (OP), and occipital fusiform gyrus (oFG)). The strongest changes in the pipelines which included ICA denoising reflected a pattern of increased connections within anterior areas (paracingulate gyrus [PcG]/ACC/FP; Figure 8d–f, Table S7d–f), and decreases centered around occipital and parietal areas; primarily between the SMG and the CC and ICC. Phase‐randomization of the regressors resulted in similar results to the simple pipeline, but with far fewer changes. The strongest changes were increases within the SMC and between this area and the precuneus/CC (Figure 8f, Table S7f).

Electrophysiological estimates derived from the power envelope showed broad, whole‐brain increases in connectivity in the delta, theta, alpha, and low beta bands, with the high beta band also showing a small number of strengthened connections (Figure 8h–l). The strongest increases in the delta band were between the FP and the insular cortex and thalamus, between the ACC and the posterior cingulate cortex (PCC) and insular cortex, and between the SMC and the PCC and precuneus (Figure 8h, Table S8a). The strongest changes in the theta band were increased connections between the mFG and the PCC, precuneus, and precentral gyrus, and between the PcG and the insular cortex and ICC (Figure 8i, Table S8b). The alpha band showed the largest increases primarily between the FP and the lOC, CC, precuneus, and ICC (Figure 8j, Table S8c). The strongest increases in the low beta band were between the lOC and the PcG, precuneus, CC, precentral gryus, inferior temporal gyrus (iFG), and temporal fusiform cortex (TFC; Figure 8k, Table S8d). In the high beta band, the strongest increases were between the FP/ACC/mFG and the PCC and precuneus (Figure 8l, Table S8e). In contrast, the connectivity estimates from the phase‐based analysis were substantially less susceptible to modulation by midazolam and primarily showed decreases in connection strengths across the bands (Figure 8m–q). The strongest decreases in the delta band occurred between the ACC and areas of the occipital cortex, and between the middle temporal gyrus (mTG) and areas of the frontal cortex (Figure 8m, Table S9a). This band also showed two strong increases, between the precuneus and lOC, and between sFG and precentral gyrus. The theta band showed the strongest reductions in connectivity between the SMC and areas of the frontal lobe, and the PcG (Figure 8n, Table S9b). Reductions in connectivity between the temporal pole (TP) and the thalamus, and between the lOC and the postcentral gyrus were found in the alpha band (Figure 8o, Table S9c). The low beta band showed the strongest reductions in connectivity between the limbic system and the FP, and between the SMC and the occipital and temporal lobes (Figure 8p, Table S9d). In the high beta band, the strongest reductions were centered anteriorly, primarily within the occipital cortex and between this area and the PCC/precuneus (Figure 8q, Table S9e).

To determine commonalities with the results from the dual regression analysis in the first part of the article, we assessed the all‐to‐all correlation changes after ketamine between nodes within the pDMN, and those within the SMN after midazolam (Figures S3 and S4). As with dual regression, reductions within the pDMN were seen primarily with pipelines which utilized ICA denoising, and increases within the SMN seen predominantly after the pipelines containing only WM/CSF/PNM regression. Repeated measures one‐way ANOVAs were performed (Mauchley's test indicated that the assumption of sphericity had been violated (pDMN: *χ* ^2^ = 58.13, *p <* .001, SMN: *χ* ^2^ = 396.68, *p* < .001), so Greenhouse–Geisser corrected tests are reported (pDMN: *ε* = .227, SMN: *ε* = .189)). Results showed that the choice of pipeline significantly affected changes in connectivity, and as with the dual regression, effect sizes were large (ketamine and pDMN: *F*_(1.3, 9.5)_ = 15.02, *p =* .002, *η* ^2^ = .682, midazolam and SMN: *F*_(1.1, 26.1)_ = 25.12, *p* > .001, *η* ^2^ = .522).

In summary, results from the fMRI pipelines containing only CSF and WM or PNM regression (SIMPLE, SIMPLE + PNM, and PNM‐only) were strikingly different to those utilizing ICA to remove spatially discrete noise sources. To formally compare the EEG and fMRI data, we next ran within‐participant correlations between the EEG and fMRI pre, post, and difference matrices (see Figure 1 for schematic). Due to the similarities between pipelines containing only CSF and WM or PNM regression and those containing ICA‐denoising, for clarity we ran this analysis on only two fMRI pipelines; those best representing these two groupings (SIMPLE and ICA‐only).

### 3.9 Comparing connectivity modulations between fMRI and EEG

**Figure 9:**
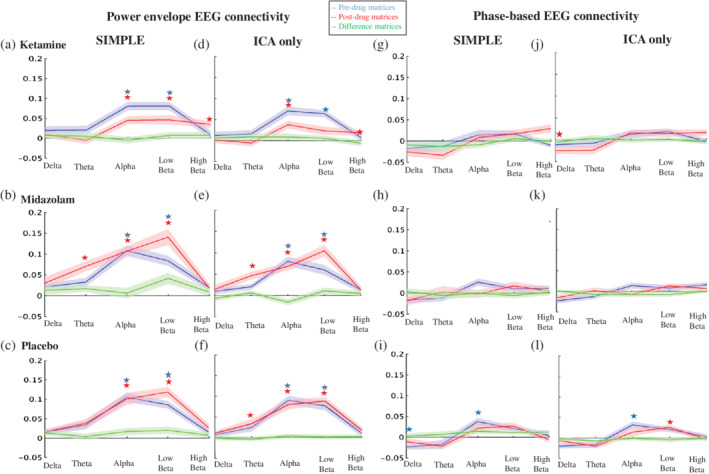
Correlations between EEG and fMRI whole‐brain connectomes. Line graphs depicting the Pearson correlation values between the upper triangles of the connectivity matrices of each band of EEG data derived from orthogonalising and correlating the power envelope (a–f), or based on phase measurements (g–l) and those from two fMRI preprocessing pipelines: one using only global signal regression pipeline (SIMPLE [a–c, g–i]) and one using no global signal regression (ICA‐only [d–f, j–l]). Stars depict significant results of one sample *t*‐tests on the EEG‐fMRI correlations (*p* < .0017), determining difference to zero, for each band. This was calculated for predrug data (blue), post‐drug (red), and on the difference (post‐pre) matrices (green)

Here the connectivity matrices and their modulations between the SIMPLE and ICA‐only fMRI pipelines (chosen to represent the two groupings of pipelines which had similar results; those containing WM and CSF or PNM regression, and those containing ICA‐denoising) and the orthogonalised power envelopes and phase‐lag index EEG data for five different frequency bands (delta, theta, alpha, low beta, high beta) were compared by correlating the all‐to‐all connectivity values between modalities for each subject and then performing population statistics (Figure 9, see Figure 1 for method schematic). Connectivity estimates for pre‐ and postdrug based on the power envelope were more closely correlated with both pipelines of fMRI data compared to wPLI. The strongest correlations were found in the alpha and low beta bands, for all three drug conditions (see Table 1 for the significant results). None of the correlations of drug contrasts (change in connectivity pre‐ vs. postadministration) reached significance.

**Table 1:** Correlations between fMRI and EEG connectivity matrices

Drug	FMRI pipeline	EEG metric	EEG band	Condition	*R*‐value	*p*‐value
Ketamine	SIMPLE	Power	Alpha	Predrug	.081	<.0001
Ketamine	SIMPLE	Power	Low‐beta	Predrug	.081	<.0001
Ketamine	SIMPLE	Power	Alpha	Postdrug	.045	<.0001
Ketamine	SIMPLE	Power	Low‐beta	Postdrug	.046	.0007
Ketamine	SIMPLE	Power	High‐beta	Postdrug	.035	.0003
Ketamine	ICA‐only	Power	Alpha	Predrug	.070	<.0001
Ketamine	ICA‐only	Power	Low‐beta	Predrug	.060	<.0001
Ketamine	ICA‐only	Power	Alpha	Postdrug	.043	<.0001
Ketamine	ICA‐only	Power	High‐beta	Postdrug	.023	.0003
Ketamine	ICA‐only	Phase	Delta	Postdrug	−.025	.0009
Midazolam	SIMPLE	Power	Alpha	Predrug	.11	<.0001
Midazolam	SIMPLE	Power	Low‐beta	Predrug	.084	<.0001
Midazolam	SIMPLE	Power	Theta	Postdrug	.070	<.0001
Midazolam	SIMPLE	Power	Alpha	Postdrug	.11	<.0001
Midazolam	SIMPLE	Power	Low‐beta	Postdrug	.14	<.0001
Midazolam	ICA‐only	Power	Alpha	Predrug	.085	<.0001
Midazolam	ICA‐only	Power	Low‐beta	Predrug	.065	<.0001
Midazolam	ICA‐only	Power	Theta	Postdrug	.049	<.0001
Midazolam	ICA‐only	Power	Alpha	Postdrug	.073	<.0001
Midazolam	ICA‐only	Power	Low‐beta	Postdrug	.11	<.0001
Placebo	SIMPLE	Power	Alpha	Predrug	.11	<.0001
Placebo	SIMPLE	Power	Low‐beta	Predrug	.086	<.0001
Placebo	SIMPLE	Power	Alpha	Postdrug	.10	<.0001
Placebo	SIMPLE	Power	Low‐beta	Postdrug	.12	<.0001
Placebo	ICA‐only	Power	Alpha	Predrug	.089	<.0001
Placebo	ICA‐only	Power	Low‐beta	Predrug	.075	<.0001
Placebo	ICA‐only	Power	Theta	Postdrug	.034	.0011
Placebo	ICA‐only	Power	Alpha	Postdrug	.079	<.0001
Placebo	ICA‐only	Power	Low‐beta	Postdrug	.085	<.0001
Placebo	SIMPLE	Phase	Delta	Predrug	−.024	.0004
Placebo	SIMPLE	Phase	Alpha	Predrug	.037	.0002
Placebo	ICA‐only	Phase	Alpha	Predrug	.032	.0003
Placebo	ICA‐only	Phase	Low‐beta	Postdrug	.025	.0001

*Note*. Displayed here are the Bonferroni‐corrected significant results of one‐sampled *t*‐tests on the *R*‐values (*SE* in brackets) for each EEG band for each comparison. These were calculated for both the SIMPLE pipeline (CSF and WM regressors) and the ICA‐only pipeline for fMRI, and the orthogonalised power envelope and phase‐based connectivity metrics for EEG.

## 4 DISCUSSION

### 4.1 Impacts of fMRI preprocessing on ICA and dual regression

This study evaluated the impact of physiological noise on the pharmacological modulation of ICA‐derived RSNs using seven preprocessing pipelines that utilized different amounts and types of noise regression. Non‐neuronal physiological processes, particularly those which lead to changes in CBF, cause global fluctuations in T2* BOLD signals (Power et al., 2018; Power, Plitt, Laumann, & Martin, 2017). These fluctuations lead to positive biases in apparent functional connectivity, which may vary across conditions in a pharmacological design (Glasser et al., 2016; Power et al., 2017). The simplest method to clean the BOLD signal is to remove the global mean (Anticevic et al., 2015; Dandash et al., 2015; Scheidegger et al., 2012), however, it has been argued that global mean regression could impact functional networks that are spatially widespread (Bright & Murphy, 2015; Glasser et al., 2018), introducing negative biases in connectivity metrics (Murphy, Birn, Handwerker, Jones, & Bandettini, 2009). Additionally, some global fluctuations have been shown to correlate with arousal state (Chang et al., 2016; Wong, DeYoung, & Liu, 2016) and global electrophysiological signals (Schölvinck, Maier, Ye, Duyn, & Leopold, 2010; Wen & Liu, 2016). The most common approach to noise removal in pharmacological fMRI studies is a more indirect method of accessing these global fluctuations: regressing out average CSF and WM signals (Bonhomme et al., 2016; Grimm et al., 2015; Joules et al., 2015; Khalili‐Mahani et al., 2015; Mueller et al., 2018; J. J. Wong, O'Daly, Mehta, Young, & Stone, 2016), like in our SIMPLE pipeline. Additionally, physiological monitoring of cardiac and respiratory cycles, and end‐tidal CO_2_ levels, can allow for the development and removal of regressors based on these variables (Birn, Diamond, Smith, & Bandettini, 2006; Chang et al., 2009; Chang & Glover, 2009; Glover et al., 2000; Shmueli et al., 2007). However, these may incompletely remove global fluctuations based on noise (Power et al., 2017). We recorded these signals and included their derived regressors in three of our pipelines: by themselves, with the CSF and WM variables, and with additional regressors derived from ICA. The visual identification of artefacts through spatial ICA (Griffanti et al., 2014) allows regression of noise sources that are spatially distinct. We ran ICA‐denoising by itself for one of our pipelines, however, mathematically spatial ICA cannot separate globally structured noise from the data (Glasser et al., 2016; Power et al., 2017) so we also included two pipelines combining ICA regressors with CSF and WM or PNM regressors. The most striking and consistent result was the large differences found between pipelines which included ICA‐denoising and those that were restricted to CSF and WM or PNM regression only.

With our placebo condition, we compared six different preprocessing pipelines to the most commonly used method in the literature; regressing out average CSF and WM signals (SIMPLE; Figures 3 and 6a). Adding PNM regressors (Figure 3c) had less of an impact than including ICA‐derived regressors (Figure 3d–f) and using PNM regressors alone (Figure 3b) often resulted in predominantly increased connectivity (SMN, VN, aDMN), possibly reflecting remaining spurious positive correlations. When additional regressors are added to a GLM, not only are the degrees of freedom reduced, the risk of mistakenly adding information that looks like signal back into the dataset is heightened. To this end, we ran a pipeline (phase‐randomized) that included the CSF and WM regressors, with the addition of artificially created noise regressors to match the number used in our most extensive pipeline (ICA + PNM; Bright & Murphy, 2015). This pipeline showed decreased connectivity compared to the SIMPLE pipeline (Figure 3g), but this was almost always limited to inside the borders of the component, potentially representing only a loss of degrees of freedom: removing signal, but not adding noise. One could conclude from this that the increased number of regressors from ICA denoising does reduce the power, however the more global reduction seen with these pipelines could be representative of the components being better described after this preprocessing step; more distinct from other areas of the brain.

While adding true noise regressors to a GLM removes variance associated with the actual confound (Power et al., 2014), it has also been demonstrated to remove additional variance at random, including what is argued to be signal because it contains distinct RSN structures (Bright & Murphy, 2015). Pharmacological studies may require different approaches due to the change in noise volume when a drug is introduced; additional nuisance regressors may become justifiable as the noise increases (Bright & Murphy, 2015). This makes comparing datasets with and without drug difficult and may result in accepting a certain amount of signal loss to more accurately compare true signal. If methods are more successful at cleaning predrug than postdrug, then the latter, noisier section may retain more spurious positive correlations, which would look like an increase in connectivity compared to predrug. Additionally, different drugs may require different amounts of cleaning depending on their modulation of physiological parameters. Indeed, the drugs used here differ in this respect; ketamine significantly increased heart rate, blood pressure, and respiratory volume, and decreased end‐tidal CO_2_, while midazolam only significantly decreased blood pressure (Figure 2), similar to previous analyses on these drugs (Michaloudis et al., 1998; Reinsel et al., 2000).

We assessed drug modulations of the RSNs for each pipeline. Ketamine reduced connectivity within all networks (Figures 4 and 6b), unlike some previous research where no changes within the VN (Niesters et al., 2012) and DMN (Mueller et al., 2018; Niesters et al., 2012) were found. Reductions similar to those found with our SIMPLE pipeline within the DMN were also found by Bonhomme et al. (2016): these authors used two dosing levels, with the lowest being most similar to ours, and their preprocessing like our SIMPLE pipeline. Interestingly, at higher doses they found further reductions in the DMN, as we did when using more extensive cleaning strategies (ICA‐denoising), and other networks also began to disintegrate. Midazolam caused increases in lower level sensory networks (SMN, VN), and decreases in two of the higher‐level cognitive networks (rFPN, pDMN; Figures 5 and 6c). These results were similar to previous studies (Greicius et al., 2008; Kiviniemi et al., 2005; Liang et al., 2015), with the exception of one finding no change in the SMN (Kiviniemi et al., 2005). Overall, the general locations where connectivity to the RSNs was significantly pharmacologically modulated, and the direction of these changes, were the same across all pipelines, however those involving only CSF or WM and PNM regression demonstrated stronger and more widespread changes (predominantly increases) after midazolam, whereas those involving more extensive cleaning (greater numbers of regressors [ICA + PNM, ICA‐only, SIMPLE + ICA]) resulted in greater modulation (predominantly decreases) after ketamine.

This pattern could represent different noise structures caused by the two drugs; more extensive cleaning on the dataset which modulated fewer physiological parameters (midazolam) removed too much signal to reveal the differences between pre‐ and postdrug, whereas when more different types of noise were involved (with ketamine) the ratio of removed signal‐to‐noise was reduced, allowing us to see changes in the remaining signal. Modulations of RVT (Birn et al., 2006), HR (Shmueli et al., 2007), and CO_2_ (Birn et al., 2006) have been shown to cause CBF fluctuations in regions with high blood volume, particularly the occipital and posterior cingulate cortices, potentially indicating that for drugs that significantly alter these variables such as ketamine, the regression of CSF and white matter signals would be inadequate to remove these confounds (Murphy et al., 2013). Midazolam only significantly changed BP, however spontaneous BP modulations have been shown to predict up to 60% of CBF fluctuations in the middle cerebral artery (Mitsis, Poulin, Robbins, & Marmarelis, 2004), and substantial transient changes cause spatially widespread modulation of BOLD activation (Harper, Bandler, Spriggs, & Alger, 2000; Kalisch, Elbel, Gössl, Czisch, & Auer, 2001; Wang et al., 2006). While cerebral autoregulatory mechanisms hold CBF constant through arterial blood pressure fluctuations of 50–150 mmHg, delays in these mechanisms can lead to alterations in CBF, confounding the BOLD signal, which may also be correlated with the autoregulatory mechanisms themselves (Kontos et al., 1978; Lang et al., 1999; Whittaker, Driver, Venzi, Bright, & Murphy, 2019). Furthermore, midazolam caused mild, un‐significant decreases in RVT and increases to CO_2,_ and hypercapnia states have been shown to slow the restoration of CBF (Aaslid, Lindegaard, Sorteberg, & Nornes, 1989). Potentially the changes to blood pressure with midazolam, unaccompanied by substantial changes in heart rate, and supplemented by mild changes to RVT and end‐tidal CO_2_ in an opposite direction to ketamine's modulations, caused global artefacts that were well described by the variance in the CSF and WM signals. Indeed, we found that regression of these signals caused a greater reduction in temporal *SD* after midazolam compared to ketamine (Table S10).

Nevertheless, a more parsimonious explanation is that the simpler preprocessing pipelines were inadequate at cleaning pharmacological simultaneous EEG/fMRI data. If the simpler pipelines were cleaning the postdrug data less effectively than the predrug, then remaining spurious positive correlations caused by physiological noise in the former may have artificially inflated connectivity increases with midazolam, and masked connectivity decreases with ketamine. Head motion at the sub‐millimeter level has been shown to cause spurious correlations in resting‐state fcfMRI studies (Maknojia, Churchill, Schweizer, & Graham, 2019; Power et al., 2014; Van Dijk, Sabuncu, & Buckner, 2012), and it becomes particularly problematic with pharmacological simultaneous imaging, due to discomfort caused by the EEG cap, and drug‐induced motion. When looking at the spatial maps of the average temporal variance (Figure S7 and Table S10), we see that while CSF and WM regression removed a substantial amount of variance in areas known to be affected by head motion from the midazolam dataset, both conditions retained high temporal variance in some of these areas until ICA‐denoising was run. Notably, in fMRI data, head motion artefact can be seen in the superior sagittal sinus, which runs between the areas making up the SMN, and this location had many voxels with high temporal variance, especially in the midazolam data. While some groups (Greicius et al., 2008; Liang et al., 2015) have found midazolam‐induced increases in BOLD SMN connectivity, the one study which scrubbed volumes of high motion (>.05 mm) found no change (Kiviniemi et al., 2005). Potentially, our fMRI results were influenced by motion artefact near the SMN, and the extensive ICA denoising allowed correction of this in the fMRI data.

In conclusion, we have shown that different preprocessing pipelines can have a substantial impact on ICA‐derived RSNs and their pharmacological modulation. Determining an ideal amount of cleaning may rely on assessing the amount and nature of physiological noise of the specific drug under investigation. However, with the current evidence available, researchers utilizing simultaneous EEG/fMRI may have to accept a certain amount of signal loss by using more extensive denoising pipelines, such as those incorporating ~50 noise regressors from ICA analysis, to be more confident that the remaining signal is accurately representing drug action.

### 4.2 Comparing whole‐brain connectomes from EEG and fMRI

We next investigated the pharmacological modulation of whole‐brain connectivity, using simultaneous EEG/fMRI, and assessed whether the patterns seen from the BOLD signal are related to those from the different EEG bands (Figures 7 and 8). When formally comparing the two modalities (Figure 9), we found the strongest relationship to the hemodynamic correlation structure, both pre‐ and postdrug, to be from the orthogonalised power envelopes in the alpha and beta bands. This was is in line with a previous nonpharmacological report (Hipp & Siegel, 2015), where dominance of the alpha and beta bands was found if not corrected for difference in SNR across frequency ranges. Deligianni et al. (2014) found that connectomes in the delta through alpha bands (1–13 Hz) best predicted rsfMRI connectomes. Connectivity based on the EEG power envelope showing a stronger relationship to fMRI than that based on phase measurements stands to reason, as changes occur over similar time scales to the BOLD signal, and the intrinsic coupling it reflects is more closely related to structural connectivity (Engel et al., 2013). Additionally, phase‐based connectivity analyses assume connectivity is instantaneous, unlike power‐based metrics, making the latter more flexible for exploratory analyses.

In the current study, no significant correlation between the pharmacological modulations of each modality was found. This could be explained in two ways; first, there may be no relationship between the pharmacological modulations of a Pearson's correlation matrix of fMRI data and our two electrophysiological connectivity metrics. This could be due to differences in the neural sources detected by the two modalities, or the changes to the fMRI connectivity could be dominated by vascular effects, overshadowing any true neural changes that could be mapped to the EEG data. Secondly, there may be statistical power issues; either limiting our ability to find a significant relationship when reducing the number of connections to those which were pharmacologically modulated as opposed to all 69,423 connections, or, alternatively, perhaps the larger number of connections when using the whole grid inflated our positive results pre‐ and postdrug. Hipp and Siegel (2015) showed that their correlations in the alpha and low‐beta bands may have been partially driven by coincidental correlations due to relatively increased power in these bands. A similar situation could have occurred here, not just with our EEG power differences, but also when we compare the two fMRI pipelines. The more extensive ICA‐denoising pipeline has fewer degrees of freedom than the SIMPLE one, which may mean that the differences in power between the two pipelines slightly inflated the SIMPLE pipeline's correlations with the EEG data.

While we found no formal relationship between the pharmacological modulations of the two modalities, if this was due to EEG's poor spatial resolution hindering the node‐for‐node analysis, particularly when the number of nodes is limited as it was in the difference matrices, then visually inspecting the overall direction and general locations of the changes may give some insight into which fMRI pipelines were modulated in a similar fashion to the EEG data. After ketamine administration, the electrophysiological metric derived from the power envelope more closely resembled changes from pipelines using ICA‐denoising; the alpha and beta bands showed reduced connectivity, primarily involving occipital and sensory‐motor areas respectively (Figure 7j,k), similar to previous findings using dynamic causal modelling (Muthukumaraswamy et al., 2015). The ICA pipelines' results (Figure 7d–f) also showed predominantly reduced connectivity across the brain, with some of the strongest changes after ICA‐ occurring within the sensory‐motor cortex. After midazolam administration, the power‐based electrophysiological connectivity more closely resembled the direction and general spatial pattern of those pipelines containing only CSF and WM or PNM regression (Figure 8a–c); global increases in connectivity were seen between 1 and 26 Hz (Figure 8h–k; Figure S1 shows the underlying spatial structure of the top 1% of connectivity changes in these bands), with the strongest increases between anterior and posterior regions. While not a formal analysis, this pattern of more cleaning (ICA pipelines) with a drug which introduced more different types of physiological noise (ketamine), and less cleaning (only CSF and WM or PNM regression) with a drug that only significantly modulated BP (midazolam) best resembling EEG results suggests that the fMRI pipeline decisions should be based on the specific noise structure of the drug. However, until the precise effects of particular noise structures are better understood, a more conservative approach utilizing extensive ICA denoising may be the more appropriate choice; accepting possible signal loss to retain confidence in residual data.

Last, it should be noted that unlike the power‐envelope correlations, the phase‐based electrophysiological connectivity metric produced far fewer changes in connectivity after drug administration; generally, these were increases after ketamine (Figure 8m–q), apart from in the alpha band as found previously (Blain‐Moraes, Lee, Ku, Noh, & Mashour, 2014; Vlisides et al., 2018), and decreases after midazolam (Figure 8m–q), also previously found (Ferrarelli et al., 2010), although a recent article found no change in overall functional connectivity using PLI (Numan et al., 2019). While the electrophysiological connectivity metric derived from the power envelope more closely resembled fMRI, this method results in a loss of some of the rich temporal information that electrophysiological methods can provide such as true zero‐phase synchrony. This would imply that continuing to use both modalities will allow us to gain a fuller view of the pharmacological modulation of neural activity.

### 4.3 Limitations and conclusions

The main limitation of this study, as with most fMRI studies, is the lack of a gold standard to compare techniques with. This was an exploratory study, aiming to assess the pharmacological modulation of connectivity by ketamine or midazolam, and how preprocessing decisions may affect these results. As such, each session was treated as a separate study when running the ICA and dual regression, to reduce the likelihood of the loss of data specific to each drug. However, running three ICAs may have introduced different noise patterns into the components, and this limited our ability to make conclusions about general modulations to the RSNs. Direct comparisons between drugs was outside the scope of this study; future research could do this by running ICA and dual regression across sessions, and for clarity use drugs that primarily differ across only one physiological parameter, and have uncomplicated pharmacology, with specific receptor affinities. Another limitation is the absence of field map collection in our protocol, which may have led to some signal distortion in regions where the magnetic field was inhomogeneous (Wilson et al., 2002). The correct denoising pipeline may need to reflect the amount and type of noise introduced by the drug, however pharmacological simultaneous imaging is particularly affected by head motion artefacts and utilizing more extensive cleaning strategies including ICA denoising can balance potential neural signal loss with the confidence that remaining signal represents true drug effect.

Defining the best parcellation for simultaneous EEG/fMRI analysis was beyond the scope of this article, and the use of 264 nodes may have caused some redundancy in the EEG connectomes due to the poor spatial resolution of this modality. However, we note that it is a trade‐off; selecting a large number of nodes reduces the risk of under‐sampling the fMRI data, as this may have missed activity represented in the EEG, whereas over‐sampling the EEG data does not result in loss of information. However, this choice may affect the statistical power to define true correlations between the modalities, hence the importance of viewing the brain‐maps and connectomes, assessing general spatial locations and directions of connectivity changes. There is the possibility that the EEG contains small motion artefacts (Fellner et al., 2016) that our rejection and correction methods were not able to capture, thereby limiting its usefulness as a direct measure of neural activity to compare the BOLD signal fluctuations with. However, our EEG pharmacological modulations look strikingly similar to previous MEG estimates (Hall, Barnes, Furlong, Seri, & Hillebrand, 2010; Muthukumaraswamy et al., 2015), although in the future the addition of hardware to better capture participant motion would be a valuable addition to studies such as this (Abbott et al., 2015; van der Meer et al., 2016). Additionally, the methods utilized in this study do not represent an exhaustive analysis of the different methodologies used to derive connectivity measures in either fMRI or EEG; the lack of a formal relationship found between the modalities of drug modulations to functional connectivity does not preclude future positive findings, which could involve using partial correlation measures to disentangle indirect and direct connections, or comparing dynamic connectivity. Nevertheless, the limited relationship between these electrophysiological metrics and that of a commonly derived fcMRI method is notable and would suggest caution in making cross‐modal conclusions based on results using these methodologies. EEG data can also help us to improve upon our fMRI preprocessing methods by providing a direct measure of neural activity, however, determining the metric that utilizes the most similar neural information as the BOLD signal requires further investigation.

## Supporting information


**Data S1**: Supporting Information

## Data Availability

Data are available from the corresponding author upon reasonable request.
