# Supplementary material for: Modulation of simultaneously collected hemodynamic and electrophysiological functional connectivity by ketamine and midazolam
Source: Hum Brain Mapp. 2019 Dec 6;41(6):1472–94. doi: 10.1002/hbm.24889 (PMC7267972; doi:10.1002/hbm.24889)
Supplement: Supplementary file 1 — Data S1: Supporting Information [file HBM-41-1472-s001.docx]

***Supplementary Figures and Tables:***

Contents

[Supplementary Table 1: Placebo - Results from ANOVA on Dual Regression Quantification 2](#_Toc24026248)

[Supplementary Table 2: KETAMINE - Results from ANOVA on Dual Regression Quantification 25](#_Toc24026249)

[Supplementary Table 3: Midazolam - Results from ANOVA on Dual Regression Quantification 52](#_Toc24026250)

[Supplementary Figure 1: Whole-brain Node Connectivity - Top one percent of changes 79](#_Toc24026251)

[Supplementary Figure 2: Whole-brain Node Connectivity - Placebo changes 80](#_Toc24026252)

[Supplementary Figure 3: Node Connectivity - Changes to pDMN 81](#_Toc24026253)

[Supplementary Figure 4: Node Connectivity - Changes to SMN 82](#_Toc24026254)

[Supplementary Table 4: Strongest ten changes in BOLD whole-brain node connectivity after Ketamine 83](#_Toc24026255)

[Supplementary Table 5: Strongest ten changes in orthogonalised power envelope EEG whole-brain node connectivity after Ketamine 87](#_Toc24026256)

[Supplementary Table 6: Strongest ten changes in WPLI EEG whole-brain node connectivity after Ketamine 89](#_Toc24026257)

[Supplementary Table 7: Strongest ten changes in BOLD whole-brain node connectivity after Midazolam 92](#_Toc24026258)

[Supplementary Table 8: Strongest ten changes in orthogonalised power envelope EEG whole-brain node connectivity after Midazolam 96](#_Toc24026259)

[Supplementary Table 9: Strongest ten changes in WPLI EEG whole-brain node connectivity after Ketamine 99](#_Toc24026260)

[Supplementary Figure 5: Spatial distribution of variance across time for the ketamine session 102](#_Toc24026261)

[Supplementary Figure 6: Spatial distribution of variance across time for the midazolam session 103](#_Toc24026262)

[Supplementary Table 10: Differences in average variance across time between pre-processing pipelines 104](#_Toc24026263)

[Supplementary Figure 7: Spatial maps of highest 75% temporal variance 105](#_Toc24026264)

# Supplementary Table 1: Placebo - Results from ANOVA on Dual Regression Quantification

|  | | Mean Difference (I-J) | Std. Error | Sig.^b^ | 95% Confidence Interval for Difference^b^ | |
| --- | --- | --- | --- | --- | --- | --- |
|  |  |  |  |  | Lower Bound | Upper Bound |
| PNM-only vs SIMPLE | SIMPLE+PNM vs SIMPLE | 8348.504^*^ | 675.628 | 0.000 | 6271.568 | 10425.440 |
|  | ICA + PNM vs SIMPLE | 66057.952^*^ | 4161.918 | 0.000 | 53263.877 | 78852.028 |
|  | ICA-only vs SIMPLE | 48901.334^*^ | 3987.237 | 0.000 | 36644.242 | 61158.427 |
|  | SIMPLE + ICA vs SIMPLE | 47017.716^*^ | 4125.224 | 0.000 | 34336.441 | 59698.990 |
|  | Phase-Randomised vs SIMPLE | 14716.072^*^ | 1020.211 | 0.000 | 11579.859 | 17852.285 |
| SIMPLE+PNM vs SIMPLE | PNM-only vs SIMPLE | -8348.504^*^ | 675.628 | 0.000 | -10425.440 | -6271.568 |
|  | ICA + PNM vs SIMPLE | 57709.448^*^ | 4143.348 | 0.000 | 44972.457 | 70446.440 |
|  | ICA-only vs SIMPLE | 40552.830^*^ | 3951.185 | 0.000 | 28406.563 | 52699.097 |
|  | SIMPLE + ICA vs SIMPLE | 38669.211^*^ | 4065.699 | 0.000 | 26170.919 | 51167.504 |
|  | Phase-Randomised vs SIMPLE | 6367.568^*^ | 1152.950 | 0.000 | 2823.306 | 9911.829 |
| ICA + PNM vs SIMPLE | PNM-only vs SIMPLE | -66057.952^*^ | 4161.918 | 0.000 | -78852.028 | -53263.877 |
|  | SIMPLE+PNM vs SIMPLE | -57709.448^*^ | 4143.348 | 0.000 | -70446.440 | -44972.457 |
|  | ICA-only vs SIMPLE | -17156.618^*^ | 756.644 | 0.000 | -19482.603 | -14830.633 |
|  | SIMPLE + ICA vs SIMPLE | -19040.237^*^ | 816.396 | 0.000 | -21549.904 | -16530.570 |
|  | Phase-Randomised vs SIMPLE | -51341.880^*^ | 3781.219 | 0.000 | -62965.658 | -39718.103 |
| ICA-only vs SIMPLE | PNM-only vs SIMPLE | -48901.334^*^ | 3987.237 | 0.000 | -61158.427 | -36644.242 |
|  | SIMPLE+PNM vs SIMPLE | -40552.830^*^ | 3951.185 | 0.000 | -52699.097 | -28406.563 |
|  | ICA + PNM vs SIMPLE | 17156.618^*^ | 756.644 | 0.000 | 14830.633 | 19482.603 |
|  | SIMPLE + ICA vs SIMPLE | -1883.619^*^ | 508.678 | 0.008 | -3447.338 | -319.900 |
|  | Phase-Randomised vs SIMPLE | -34185.262^*^ | 3601.222 | 0.000 | -45255.712 | -23114.812 |
| SIMPLE + ICA vs SIMPLE | PNM-only vs SIMPLE | -47017.716^*^ | 4125.224 | 0.000 | -59698.990 | -34336.441 |
|  | SIMPLE+PNM vs SIMPLE | -38669.211^*^ | 4065.699 | 0.000 | -51167.504 | -26170.919 |
|  | ICA + PNM vs SIMPLE | 19040.237^*^ | 816.396 | 0.000 | 16530.570 | 21549.904 |
|  | ICA-only vs SIMPLE | 1883.619^*^ | 508.678 | 0.008 | 319.900 | 3447.338 |
|  | Phase-Randomised vs SIMPLE | -32301.644^*^ | 3740.019 | 0.000 | -43798.768 | -20804.520 |
| Phase-Randomised vs SIMPLE | PNM-only vs SIMPLE | -14716.072^*^ | 1020.211 | 0.000 | -17852.285 | -11579.859 |
|  | SIMPLE+PNM vs SIMPLE | -6367.568^*^ | 1152.950 | 0.000 | -9911.829 | -2823.306 |
|  | ICA + PNM vs SIMPLE | 51341.880^*^ | 3781.219 | 0.000 | 39718.103 | 62965.658 |
|  | ICA-only vs SIMPLE | 34185.262^*^ | 3601.222 | 0.000 | 23114.812 | 45255.712 |
|  | SIMPLE + ICA vs SIMPLE | 32301.644^*^ | 3740.019 | 0.000 | 20804.520 | 43798.768 |
| Based on estimated marginal means | | | | | | |
| *. The mean difference is significant at the .05 level. | | | | | | |
| b. Adjustment for multiple comparisons: Bonferroni. | | | | | | |

**Supplementary Table 1A.)** Post-hoc paired *t*-tests between all contrasts of pre-processing pipelines vs the SIMPLE pipeline, across RSNs, for connectivity changes within RSNs

| (I) Pipeline | | Mean Difference (I-J) | Std. Error | Sig.^b^ | 95% Confidence Interval for Difference^b^ | |
| --- | --- | --- | --- | --- | --- | --- |
|  |  |  |  |  | Lower Bound | Upper Bound |
| PNM-only vs SIMPLE | SIMPLE+PNM vs SIMPLE | 15970.158^*^ | 2256.955 | 0.000 | 9032.093 | 22908.224 |
|  | ICA + PNM vs SIMPLE | 134876.979^*^ | 7305.185 | 0.000 | 112420.244 | 157333.714 |
|  | ICA-only vs SIMPLE | 120012.351^*^ | 7272.297 | 0.000 | 97656.716 | 142367.986 |
|  | SIMPLE + ICA vs SIMPLE | 87618.491^*^ | 5896.634 | 0.000 | 69491.756 | 105745.225 |
|  | Phase-Randomised vs SIMPLE | 21787.322^*^ | 2014.093 | 0.000 | 15595.836 | 27978.808 |
| SIMPLE+PNM vs SIMPLE | PNM-only vs SIMPLE | -15970.158^*^ | 2256.955 | 0.000 | -22908.224 | -9032.093 |
|  | ICA + PNM vs SIMPLE | 118906.820^*^ | 7483.241 | 0.000 | 95902.725 | 141910.916 |
|  | ICA-only vs SIMPLE | 104042.193^*^ | 7438.811 | 0.000 | 81174.678 | 126909.708 |
|  | SIMPLE + ICA vs SIMPLE | 71648.332^*^ | 5949.459 | 0.000 | 53359.210 | 89937.455 |
|  | Phase-Randomised vs SIMPLE | 5817.164^*^ | 1194.179 | 0.000 | 2146.161 | 9488.167 |
| ICA + PNM vs SIMPLE | PNM-only vs SIMPLE | -134876.979^*^ | 7305.185 | 0.000 | -157333.714 | -112420.244 |
|  | SIMPLE+PNM vs SIMPLE | -118906.820^*^ | 7483.241 | 0.000 | -141910.916 | -95902.725 |
|  | ICA-only vs SIMPLE | -14864.628^*^ | 865.930 | 0.000 | -17526.568 | -12202.688 |
|  | SIMPLE + ICA vs SIMPLE | -47258.488^*^ | 1944.288 | 0.000 | -53235.387 | -41281.589 |
|  | Phase-Randomised vs SIMPLE | -113089.656^*^ | 7057.168 | 0.000 | -134783.967 | -91395.345 |
| ICA-only vs SIMPLE | PNM-only vs SIMPLE | -120012.351^*^ | 7272.297 | 0.000 | -142367.986 | -97656.716 |
|  | SIMPLE+PNM vs SIMPLE | -104042.193^*^ | 7438.811 | 0.000 | -126909.708 | -81174.678 |
|  | ICA + PNM vs SIMPLE | 14864.628^*^ | 865.930 | 0.000 | 12202.688 | 17526.568 |
|  | SIMPLE + ICA vs SIMPLE | -32393.860^*^ | 1951.584 | 0.000 | -38393.189 | -26394.532 |
|  | Phase-Randomised vs SIMPLE | -98225.029^*^ | 7031.916 | 0.000 | -119841.713 | -76608.344 |
| SIMPLE + ICA vs SIMPLE | PNM-only vs SIMPLE | -87618.491^*^ | 5896.634 | 0.000 | -105745.225 | -69491.756 |
|  | SIMPLE+PNM vs SIMPLE | -71648.332^*^ | 5949.459 | 0.000 | -89937.455 | -53359.210 |
|  | ICA + PNM vs SIMPLE | 47258.488^*^ | 1944.288 | 0.000 | 41281.589 | 53235.387 |
|  | ICA-only vs SIMPLE | 32393.860^*^ | 1951.584 | 0.000 | 26394.532 | 38393.189 |
|  | Phase-Randomised vs SIMPLE | -65831.168^*^ | 5558.487 | 0.000 | -82918.412 | -48743.925 |
| Phase-Randomised vs SIMPLE | PNM-only vs SIMPLE | -21787.322^*^ | 2014.093 | 0.000 | -27978.808 | -15595.836 |
|  | SIMPLE+PNM vs SIMPLE | -5817.164^*^ | 1194.179 | 0.000 | -9488.167 | -2146.161 |
|  | ICA + PNM vs SIMPLE | 113089.656^*^ | 7057.168 | 0.000 | 91395.345 | 134783.967 |
|  | ICA-only vs SIMPLE | 98225.029^*^ | 7031.916 | 0.000 | 76608.344 | 119841.713 |
|  | SIMPLE + ICA vs SIMPLE | 65831.168^*^ | 5558.487 | 0.000 | 48743.925 | 82918.412 |
| Based on estimated marginal means | | | | | | |
| *. The mean difference is significant at the .05 level. | | | | | | |
| b. Adjustment for multiple comparisons: Bonferroni. | | | | | | |

**Supplementary Table 1B.)** Post-hoc paired *t*-tests between all contrasts of pre-processing pipelines vs the SIMPLE pipeline, across RSNs for connectivity changes between RSNs and the rest of the brain

| Pipeline | Mean | Std. Error | 95% Confidence Interval | |
| --- | --- | --- | --- | --- |
|  |  |  | Lower Bound | Upper Bound |
| PNM-only | -10205.213 | 730.420 | -11670.250 | -8740.177 |
| SIMPLE+PNM | -18553.717 | 1035.938 | -20631.545 | -16475.890 |
| ICA + PNM | -76263.166 | 4552.981 | -85395.288 | -67131.043 |
| ICA-only | -59106.547 | 4356.401 | -67844.381 | -50368.714 |
| SIMPLE + ICA | -57222.929 | 4486.057 | -66220.820 | -48225.038 |
| Phase-randomised | -24921.285 | 1218.911 | -27366.112 | -22476.459 |

**Supplementary Table 1C.)** Average changes in connectivity within RSNS, across RSNs, for each pipeline compared to the SIMPLE pipeline

| Pipeline | Mean | Std. Error | 95% Confidence Interval | |
| --- | --- | --- | --- | --- |
|  |  |  | Lower Bound | Upper Bound |
| PNM-only | 82.816 | 2116.321 | -4161.986 | 4327.619 |
| SIMPLE+PNM | -15887.342 | 981.053 | -17855.086 | -13919.598 |
| ICA + PNM | -134794.162 | 7898.871 | -150637.292 | -118951.033 |
| ICA-only | -119929.535 | 7868.529 | -135711.805 | -104147.265 |
| SIMPLE + ICA | -87535.674 | 6379.233 | -100330.796 | -74740.553 |
| Phase-randomised | -21704.506 | 1098.353 | -23907.524 | -19501.488 |

**Supplementary Table 1D.)** Average changes in connectivity between RSNs and the rest of the brain, across RSNs, for each pipeline compared to the SIMPLE pipeline

| RSN | | | Mean Difference (I-J) | Std. Error | Sig.^b^ | 95% Confidence Interval for Difference^b^ | |
| --- | --- | --- | --- | --- | --- | --- | --- |
|  |  |  |  |  |  | Lower Bound | Upper Bound |
| rFPN | PNM-only vs SIMPLE | SIMPLE+PNM vs SIMPLE | 5420.490^*^ | 1297.231 | 0.002 | 1432.697 | 9408.283 |
|  |  | ICA + PNM vs SIMPLE | 55770.527^*^ | 4614.523 | 0.000 | 41585.105 | 69955.949 |
|  |  | ICA-only vs SIMPLE | 36725.354^*^ | 4014.722 | 0.000 | 24383.770 | 49066.939 |
|  |  | SIMPLE + ICA vs SIMPLE | 35438.395^*^ | 4154.128 | 0.000 | 22668.265 | 48208.525 |
|  |  | Phase-Randomised vs SIMPLE | 8994.890^*^ | 1856.246 | 0.000 | 3288.638 | 14701.142 |
|  | SIMPLE+PNM vs SIMPLE | PNM-only vs SIMPLE | -5420.490^*^ | 1297.231 | 0.002 | -9408.283 | -1432.697 |
|  |  | ICA + PNM vs SIMPLE | 50350.037^*^ | 4633.467 | 0.000 | 36106.381 | 64593.693 |
|  |  | ICA-only vs SIMPLE | 31304.865^*^ | 4005.524 | 0.000 | 18991.556 | 43618.174 |
|  |  | SIMPLE + ICA vs SIMPLE | 30017.905^*^ | 4112.782 | 0.000 | 17374.878 | 42660.933 |
|  |  | Phase-Randomised vs SIMPLE | 3574.400 | 1712.301 | 0.625 | -1689.353 | 8838.154 |
|  | ICA + PNM vs SIMPLE | PNM-only vs SIMPLE | -55770.527^*^ | 4614.523 | 0.000 | -69955.949 | -41585.105 |
|  |  | SIMPLE+PNM vs SIMPLE | -50350.037^*^ | 4633.467 | 0.000 | -64593.693 | -36106.381 |
|  |  | ICA-only vs SIMPLE | -19045.172^*^ | 1324.023 | 0.000 | -23115.327 | -14975.018 |
|  |  | SIMPLE + ICA vs SIMPLE | -20332.132^*^ | 1459.420 | 0.000 | -24818.508 | -15845.756 |
|  |  | Phase-Randomised vs SIMPLE | -46775.637^*^ | 4802.425 | 0.000 | -61538.683 | -32012.590 |
|  | ICA-only vs SIMPLE | PNM-only vs SIMPLE | -36725.354^*^ | 4014.722 | 0.000 | -49066.939 | -24383.770 |
|  |  | SIMPLE+PNM vs SIMPLE | -31304.865^*^ | 4005.524 | 0.000 | -43618.174 | -18991.556 |
|  |  | ICA + PNM vs SIMPLE | 19045.172^*^ | 1324.023 | 0.000 | 14975.018 | 23115.327 |
|  |  | SIMPLE + ICA vs SIMPLE | -1286.959 | 1030.694 | 1.000 | -4455.396 | 1881.478 |
|  |  | Phase-Randomised vs SIMPLE | -27730.464^*^ | 4142.094 | 0.000 | -40463.599 | -14997.329 |
|  | SIMPLE + ICA vs SIMPLE | PNM-only vs SIMPLE | -35438.395^*^ | 4154.128 | 0.000 | -48208.525 | -22668.265 |
|  |  | SIMPLE+PNM vs SIMPLE | -30017.905^*^ | 4112.782 | 0.000 | -42660.933 | -17374.878 |
|  |  | ICA + PNM vs SIMPLE | 20332.132^*^ | 1459.420 | 0.000 | 15845.756 | 24818.508 |
|  |  | ICA-only vs SIMPLE | 1286.959 | 1030.694 | 1.000 | -1881.478 | 4455.396 |
|  |  | Phase-Randomised vs SIMPLE | -26443.505^*^ | 4362.631 | 0.000 | -39854.590 | -13032.419 |
|  | Phase-Randomised vs SIMPLE | PNM-only vs SIMPLE | -8994.890^*^ | 1856.246 | 0.000 | -14701.142 | -3288.638 |
|  |  | SIMPLE+PNM vs SIMPLE | -3574.400 | 1712.301 | 0.625 | -8838.154 | 1689.353 |
|  |  | ICA + PNM vs SIMPLE | 46775.637^*^ | 4802.425 | 0.000 | 32012.590 | 61538.683 |
|  |  | ICA-only vs SIMPLE | 27730.464^*^ | 4142.094 | 0.000 | 14997.329 | 40463.599 |
|  |  | SIMPLE + ICA vs SIMPLE | 26443.505^*^ | 4362.631 | 0.000 | 13032.419 | 39854.590 |
| lFPN | PNM-only vs SIMPLE | SIMPLE+PNM vs SIMPLE | -6860.031^*^ | 1731.672 | 0.003 | -12183.332 | -1536.730 |
|  |  | ICA + PNM vs SIMPLE | 40114.664^*^ | 3458.642 | 0.000 | 29482.515 | 50746.812 |
|  |  | ICA-only vs SIMPLE | 29823.785^*^ | 3544.061 | 0.000 | 18929.053 | 40718.517 |
|  |  | SIMPLE + ICA vs SIMPLE | 29070.642^*^ | 3667.077 | 0.000 | 17797.746 | 40343.538 |
|  |  | Phase-Randomised vs SIMPLE | -4310.291 | 2431.635 | 1.000 | -11785.338 | 3164.755 |
|  | SIMPLE+PNM vs SIMPLE | PNM-only vs SIMPLE | 6860.031^*^ | 1731.672 | 0.003 | 1536.730 | 12183.332 |
|  |  | ICA + PNM vs SIMPLE | 46974.695^*^ | 3939.989 | 0.000 | 34862.845 | 59086.544 |
|  |  | ICA-only vs SIMPLE | 36683.816^*^ | 3973.371 | 0.000 | 24469.347 | 48898.284 |
|  |  | SIMPLE + ICA vs SIMPLE | 35930.673^*^ | 3937.200 | 0.000 | 23827.399 | 48033.947 |
|  |  | Phase-Randomised vs SIMPLE | 2549.740 | 1286.115 | 0.789 | -1403.883 | 6503.363 |
|  | ICA + PNM vs SIMPLE | PNM-only vs SIMPLE | -40114.664^*^ | 3458.642 | 0.000 | -50746.812 | -29482.515 |
|  |  | SIMPLE+PNM vs SIMPLE | -46974.695^*^ | 3939.989 | 0.000 | -59086.544 | -34862.845 |
|  |  | ICA-only vs SIMPLE | -10290.879^*^ | 871.624 | 0.000 | -12970.321 | -7611.437 |
|  |  | SIMPLE + ICA vs SIMPLE | -11044.022^*^ | 1090.046 | 0.000 | -14394.911 | -7693.132 |
|  |  | Phase-Randomised vs SIMPLE | -44424.955^*^ | 4253.170 | 0.000 | -57499.547 | -31350.363 |
|  | ICA-only vs SIMPLE | PNM-only vs SIMPLE | -29823.785^*^ | 3544.061 | 0.000 | -40718.517 | -18929.053 |
|  |  | SIMPLE+PNM vs SIMPLE | -36683.816^*^ | 3973.371 | 0.000 | -48898.284 | -24469.347 |
|  |  | ICA + PNM vs SIMPLE | 10290.879^*^ | 871.624 | 0.000 | 7611.437 | 12970.321 |
|  |  | SIMPLE + ICA vs SIMPLE | -753.143 | 961.283 | 1.000 | -3708.206 | 2201.920 |
|  |  | Phase-Randomised vs SIMPLE | -34134.076^*^ | 4256.992 | 0.000 | -47220.418 | -21047.734 |
|  | SIMPLE + ICA vs SIMPLE | PNM-only vs SIMPLE | -29070.642^*^ | 3667.077 | 0.000 | -40343.538 | -17797.746 |
|  |  | SIMPLE+PNM vs SIMPLE | -35930.673^*^ | 3937.200 | 0.000 | -48033.947 | -23827.399 |
|  |  | ICA + PNM vs SIMPLE | 11044.022^*^ | 1090.046 | 0.000 | 7693.132 | 14394.911 |
|  |  | ICA-only vs SIMPLE | 753.143 | 961.283 | 1.000 | -2201.920 | 3708.206 |
|  |  | Phase-Randomised vs SIMPLE | -33380.933^*^ | 4200.509 | 0.000 | -46293.641 | -20468.226 |
|  | Phase-Randomised vs SIMPLE | PNM-only vs SIMPLE | 4310.291 | 2431.635 | 1.000 | -3164.755 | 11785.338 |
|  |  | SIMPLE+PNM vs SIMPLE | -2549.740 | 1286.115 | 0.789 | -6503.363 | 1403.883 |
|  |  | ICA + PNM vs SIMPLE | 44424.955^*^ | 4253.170 | 0.000 | 31350.363 | 57499.547 |
|  |  | ICA-only vs SIMPLE | 34134.076^*^ | 4256.992 | 0.000 | 21047.734 | 47220.418 |
|  |  | SIMPLE + ICA vs SIMPLE | 33380.933^*^ | 4200.509 | 0.000 | 20468.226 | 46293.641 |
| SMN | PNM-only vs SIMPLE | SIMPLE+PNM vs SIMPLE | 12574.291^*^ | 1851.833 | 0.000 | 6881.606 | 18266.976 |
|  |  | ICA + PNM vs SIMPLE | 108274.671^*^ | 8866.227 | 0.000 | 81019.162 | 135530.179 |
|  |  | ICA-only vs SIMPLE | 82040.013^*^ | 8225.730 | 0.000 | 56753.445 | 107326.581 |
|  |  | SIMPLE + ICA vs SIMPLE | 80262.899^*^ | 9008.087 | 0.000 | 52571.302 | 107954.496 |
|  |  | Phase-Randomised vs SIMPLE | 21794.649^*^ | 2653.394 | 0.000 | 13637.898 | 29951.400 |
|  | SIMPLE+PNM vs SIMPLE | PNM-only vs SIMPLE | -12574.291^*^ | 1851.833 | 0.000 | -18266.976 | -6881.606 |
|  |  | ICA + PNM vs SIMPLE | 95700.380^*^ | 8719.655 | 0.000 | 68895.446 | 122505.313 |
|  |  | ICA-only vs SIMPLE | 69465.722^*^ | 7974.067 | 0.000 | 44952.789 | 93978.655 |
|  |  | SIMPLE + ICA vs SIMPLE | 67688.608^*^ | 8742.929 | 0.000 | 40812.129 | 94565.087 |
|  |  | Phase-Randomised vs SIMPLE | 9220.358^*^ | 2352.478 | 0.004 | 1988.647 | 16452.069 |
|  | ICA + PNM vs SIMPLE | PNM-only vs SIMPLE | -108274.671^*^ | 8866.227 | 0.000 | -135530.179 | -81019.162 |
|  |  | SIMPLE+PNM vs SIMPLE | -95700.380^*^ | 8719.655 | 0.000 | -122505.313 | -68895.446 |
|  |  | ICA-only vs SIMPLE | -26234.658^*^ | 3623.870 | 0.000 | -37374.730 | -15094.586 |
|  |  | SIMPLE + ICA vs SIMPLE | -28011.772^*^ | 3375.437 | 0.000 | -38388.141 | -17635.402 |
|  |  | Phase-Randomised vs SIMPLE | -86480.021^*^ | 8138.254 | 0.000 | -111497.680 | -61462.363 |
|  | ICA-only vs SIMPLE | PNM-only vs SIMPLE | -82040.013^*^ | 8225.730 | 0.000 | -107326.581 | -56753.445 |
|  |  | SIMPLE+PNM vs SIMPLE | -69465.722^*^ | 7974.067 | 0.000 | -93978.655 | -44952.789 |
|  |  | ICA + PNM vs SIMPLE | 26234.658^*^ | 3623.870 | 0.000 | 15094.586 | 37374.730 |
|  |  | SIMPLE + ICA vs SIMPLE | -1777.114 | 1543.084 | 1.000 | -6520.679 | 2966.452 |
|  |  | Phase-Randomised vs SIMPLE | -60245.363^*^ | 7398.977 | 0.000 | -82990.423 | -37500.304 |
|  | SIMPLE + ICA vs SIMPLE | PNM-only vs SIMPLE | -80262.899^*^ | 9008.087 | 0.000 | -107954.496 | -52571.302 |
|  |  | SIMPLE+PNM vs SIMPLE | -67688.608^*^ | 8742.929 | 0.000 | -94565.087 | -40812.129 |
|  |  | ICA + PNM vs SIMPLE | 28011.772^*^ | 3375.437 | 0.000 | 17635.402 | 38388.141 |
|  |  | ICA-only vs SIMPLE | 1777.114 | 1543.084 | 1.000 | -2966.452 | 6520.679 |
|  |  | Phase-Randomised vs SIMPLE | -58468.250^*^ | 8116.350 | 0.000 | -83418.574 | -33517.926 |
|  | Phase-Randomised vs SIMPLE | PNM-only vs SIMPLE | -21794.649^*^ | 2653.394 | 0.000 | -29951.400 | -13637.898 |
|  |  | SIMPLE+PNM vs SIMPLE | -9220.358^*^ | 2352.478 | 0.004 | -16452.069 | -1988.647 |
|  |  | ICA + PNM vs SIMPLE | 86480.021^*^ | 8138.254 | 0.000 | 61462.363 | 111497.680 |
|  |  | ICA-only vs SIMPLE | 60245.363^*^ | 7398.977 | 0.000 | 37500.304 | 82990.423 |
|  |  | SIMPLE + ICA vs SIMPLE | 58468.250^*^ | 8116.350 | 0.000 | 33517.926 | 83418.574 |
| VN | PNM-only vs SIMPLE | SIMPLE+PNM vs SIMPLE | 10439.364^*^ | 933.068 | 0.000 | 7571.035 | 13307.693 |
|  |  | ICA + PNM vs SIMPLE | 57396.032^*^ | 5561.963 | 0.000 | 40298.103 | 74493.960 |
|  |  | ICA + PNM vs SIMPLE | 41962.898^*^ | 5212.494 | 0.000 | 25939.265 | 57986.531 |
|  |  | SIMPLE + ICA vs SIMPLE | 39517.061^*^ | 5113.460 | 0.000 | 23797.868 | 55236.255 |
|  |  | Phase-Randomised vs SIMPLE | 24937.432^*^ | 1815.945 | 0.000 | 19355.068 | 30519.797 |
|  | SIMPLE+PNM vs SIMPLE | PNM-only vs SIMPLE | -10439.364^*^ | 933.068 | 0.000 | -13307.693 | -7571.035 |
|  |  | ICA + PNM vs SIMPLE | 46956.668^*^ | 5399.781 | 0.000 | 30357.298 | 63556.037 |
|  |  | ICA + PNM vs SIMPLE | 31523.534^*^ | 5115.356 | 0.000 | 15798.512 | 47248.555 |
|  |  | SIMPLE + ICA vs SIMPLE | 29077.697^*^ | 5063.568 | 0.000 | 13511.874 | 44643.520 |
|  |  | Phase-Randomised vs SIMPLE | 14498.068^*^ | 1672.294 | 0.000 | 9357.300 | 19638.837 |
|  | ICA + PNM vs SIMPLE | PNM-only vs SIMPLE | -57396.032^*^ | 5561.963 | 0.000 | -74493.960 | -40298.103 |
|  |  | SIMPLE+PNM vs SIMPLE | -46956.668^*^ | 5399.781 | 0.000 | -63556.037 | -30357.298 |
|  |  | ICA + PNM vs SIMPLE | -15433.134^*^ | 1149.296 | 0.000 | -18966.163 | -11900.105 |
|  |  | SIMPLE + ICA vs SIMPLE | -17878.970^*^ | 1380.864 | 0.000 | -22123.859 | -13634.082 |
|  |  | Phase-Randomised vs SIMPLE | -32458.599^*^ | 4724.090 | 0.000 | -46980.838 | -17936.361 |
|  | ICA + PNM vs SIMPLE | PNM-only vs SIMPLE | -41962.898^*^ | 5212.494 | 0.000 | -57986.531 | -25939.265 |
|  |  | SIMPLE+PNM vs SIMPLE | -31523.534^*^ | 5115.356 | 0.000 | -47248.555 | -15798.512 |
|  |  | ICA + PNM vs SIMPLE | 15433.134^*^ | 1149.296 | 0.000 | 11900.105 | 18966.163 |
|  |  | SIMPLE + ICA vs SIMPLE | -2445.837 | 852.625 | 0.089 | -5066.877 | 175.204 |
|  |  | Phase-Randomised vs SIMPLE | -17025.466^*^ | 4404.482 | 0.005 | -30565.204 | -3485.727 |
|  | SIMPLE + ICA vs SIMPLE | PNM-only vs SIMPLE | -39517.061^*^ | 5113.460 | 0.000 | -55236.255 | -23797.868 |
|  |  | SIMPLE+PNM vs SIMPLE | -29077.697^*^ | 5063.568 | 0.000 | -44643.520 | -13511.874 |
|  |  | ICA + PNM vs SIMPLE | 17878.970^*^ | 1380.864 | 0.000 | 13634.082 | 22123.859 |
|  |  | ICA + PNM vs SIMPLE | 2445.837 | 852.625 | 0.089 | -175.204 | 5066.877 |
|  |  | Phase-Randomised vs SIMPLE | -14579.629^*^ | 4334.876 | 0.022 | -27905.394 | -1253.864 |
|  | Phase-Randomised vs SIMPLE | PNM-only vs SIMPLE | -24937.432^*^ | 1815.945 | 0.000 | -30519.797 | -19355.068 |
|  |  | SIMPLE+PNM vs SIMPLE | -14498.068^*^ | 1672.294 | 0.000 | -19638.837 | -9357.300 |
|  |  | ICA + PNM vs SIMPLE | 32458.599^*^ | 4724.090 | 0.000 | 17936.361 | 46980.838 |
|  |  | ICA + PNM vs SIMPLE | 17025.466^*^ | 4404.482 | 0.005 | 3485.727 | 30565.204 |
|  |  | SIMPLE + ICA vs SIMPLE | 14579.629^*^ | 4334.876 | 0.022 | 1253.864 | 27905.394 |
| pDMN | PNM-only vs SIMPLE | SIMPLE+PNM vs SIMPLE | 10045.262^*^ | 1545.638 | 0.000 | 5293.845 | 14796.679 |
|  |  | ICA + PNM vs SIMPLE | 60311.705^*^ | 4297.334 | 0.000 | 47101.349 | 73522.061 |
|  |  | ICA + PNM vs SIMPLE | 43905.058^*^ | 4381.078 | 0.000 | 30437.266 | 57372.849 |
|  |  | SIMPLE + ICA vs SIMPLE | 40757.975^*^ | 4484.783 | 0.000 | 26971.384 | 54544.566 |
|  |  | Phase-Randomised vs SIMPLE | 19192.639^*^ | 2173.394 | 0.000 | 12511.449 | 25873.830 |
|  | SIMPLE+PNM vs SIMPLE | PNM-only vs SIMPLE | -10045.262^*^ | 1545.638 | 0.000 | -14796.679 | -5293.845 |
|  |  | ICA + PNM vs SIMPLE | 50266.443^*^ | 3514.267 | 0.000 | 39463.298 | 61069.588 |
|  |  | ICA + PNM vs SIMPLE | 33859.796^*^ | 3656.181 | 0.000 | 22620.397 | 45099.194 |
|  |  | SIMPLE + ICA vs SIMPLE | 30712.713^*^ | 3705.793 | 0.000 | 19320.804 | 42104.623 |
|  |  | Phase-Randomised vs SIMPLE | 9147.377^*^ | 2114.941 | 0.001 | 2645.875 | 15648.880 |
|  | ICA + PNM vs SIMPLE | PNM-only vs SIMPLE | -60311.705^*^ | 4297.334 | 0.000 | -73522.061 | -47101.349 |
|  |  | SIMPLE+PNM vs SIMPLE | -50266.443^*^ | 3514.267 | 0.000 | -61069.588 | -39463.298 |
|  |  | ICA + PNM vs SIMPLE | -16406.647^*^ | 972.605 | 0.000 | -19396.515 | -13416.780 |
|  |  | SIMPLE + ICA vs SIMPLE | -19553.730^*^ | 1047.459 | 0.000 | -22773.704 | -16333.756 |
|  |  | Phase-Randomised vs SIMPLE | -41119.066^*^ | 3739.304 | 0.000 | -52613.992 | -29624.139 |
|  | ICA + PNM vs SIMPLE | PNM-only vs SIMPLE | -43905.058^*^ | 4381.078 | 0.000 | -57372.849 | -30437.266 |
|  |  | SIMPLE+PNM vs SIMPLE | -33859.796^*^ | 3656.181 | 0.000 | -45099.194 | -22620.397 |
|  |  | ICA + PNM vs SIMPLE | 16406.647^*^ | 972.605 | 0.000 | 13416.780 | 19396.515 |
|  |  | SIMPLE + ICA vs SIMPLE | -3147.082^*^ | 772.266 | 0.002 | -5521.090 | -773.075 |
|  |  | Phase-Randomised vs SIMPLE | -24712.418^*^ | 3862.039 | 0.000 | -36584.641 | -12840.196 |
|  | SIMPLE + ICA vs SIMPLE | PNM-only vs SIMPLE | -40757.975^*^ | 4484.783 | 0.000 | -54544.566 | -26971.384 |
|  |  | SIMPLE+PNM vs SIMPLE | -30712.713^*^ | 3705.793 | 0.000 | -42104.623 | -19320.804 |
|  |  | ICA + PNM vs SIMPLE | 19553.730^*^ | 1047.459 | 0.000 | 16333.756 | 22773.704 |
|  |  | ICA + PNM vs SIMPLE | 3147.082^*^ | 772.266 | 0.002 | 773.075 | 5521.090 |
|  |  | Phase-Randomised vs SIMPLE | -21565.336^*^ | 3880.818 | 0.000 | -33495.287 | -9635.384 |
|  | Phase-Randomised vs SIMPLE | PNM-only vs SIMPLE | -19192.639^*^ | 2173.394 | 0.000 | -25873.830 | -12511.449 |
|  |  | SIMPLE+PNM vs SIMPLE | -9147.377^*^ | 2114.941 | 0.001 | -15648.880 | -2645.875 |
|  |  | ICA + PNM vs SIMPLE | 41119.066^*^ | 3739.304 | 0.000 | 29624.139 | 52613.992 |
|  |  | ICA + PNM vs SIMPLE | 24712.418^*^ | 3862.039 | 0.000 | 12840.196 | 36584.641 |
|  |  | SIMPLE + ICA vs SIMPLE | 21565.336^*^ | 3880.818 | 0.000 | 9635.384 | 33495.287 |
| aDMN | PNM-only vs SIMPLE | SIMPLE+PNM vs SIMPLE | 18471.650^*^ | 1357.735 | 0.000 | 14297.862 | 22645.437 |
|  |  | ICA + PNM vs SIMPLE | 74480.116^*^ | 5794.705 | 0.000 | 56666.718 | 92293.515 |
|  |  | ICA + PNM vs SIMPLE | 58950.898^*^ | 5530.513 | 0.000 | 41949.648 | 75952.148 |
|  |  | SIMPLE + ICA vs SIMPLE | 57059.322^*^ | 5235.433 | 0.000 | 40965.173 | 73153.470 |
|  |  | Phase-Randomised vs SIMPLE | 17687.112^*^ | 1881.573 | 0.000 | 11903.004 | 23471.221 |
|  | SIMPLE+PNM vs SIMPLE | PNM-only vs SIMPLE | -18471.650^*^ | 1357.735 | 0.000 | -22645.437 | -14297.862 |
|  |  | ICA + PNM vs SIMPLE | 56008.467^*^ | 5644.230 | 0.000 | 38657.643 | 73359.291 |
|  |  | ICA + PNM vs SIMPLE | 40479.249^*^ | 5492.567 | 0.000 | 23594.648 | 57363.849 |
|  |  | SIMPLE + ICA vs SIMPLE | 38587.672^*^ | 5136.316 | 0.000 | 22798.217 | 54377.127 |
|  |  | Phase-Randomised vs SIMPLE | -784.537 | 2248.681 | 1.000 | -7697.167 | 6128.093 |
|  | ICA + PNM vs SIMPLE | PNM-only vs SIMPLE | -74480.116^*^ | 5794.705 | 0.000 | -92293.515 | -56666.718 |
|  |  | SIMPLE+PNM vs SIMPLE | -56008.467^*^ | 5644.230 | 0.000 | -73359.291 | -38657.643 |
|  |  | ICA + PNM vs SIMPLE | -15529.218^*^ | 1078.214 | 0.000 | -18843.736 | -12214.700 |
|  |  | SIMPLE + ICA vs SIMPLE | -17420.795^*^ | 1108.002 | 0.000 | -20826.884 | -14014.706 |
|  |  | Phase-Randomised vs SIMPLE | -56793.004^*^ | 5207.328 | 0.000 | -72800.758 | -40785.251 |
|  | ICA + PNM vs SIMPLE | PNM-only vs SIMPLE | -58950.898^*^ | 5530.513 | 0.000 | -75952.148 | -41949.648 |
|  |  | SIMPLE+PNM vs SIMPLE | -40479.249^*^ | 5492.567 | 0.000 | -57363.849 | -23594.648 |
|  |  | ICA + PNM vs SIMPLE | 15529.218^*^ | 1078.214 | 0.000 | 12214.700 | 18843.736 |
|  |  | SIMPLE + ICA vs SIMPLE | -1891.577 | 716.894 | 0.164 | -4095.368 | 312.215 |
|  |  | Phase-Randomised vs SIMPLE | -41263.786^*^ | 4952.932 | 0.000 | -56489.503 | -26038.069 |
|  | SIMPLE + ICA vs SIMPLE | PNM-only vs SIMPLE | -57059.322^*^ | 5235.433 | 0.000 | -73153.470 | -40965.173 |
|  |  | SIMPLE+PNM vs SIMPLE | -38587.672^*^ | 5136.316 | 0.000 | -54377.127 | -22798.217 |
|  |  | ICA + PNM vs SIMPLE | 17420.795^*^ | 1108.002 | 0.000 | 14014.706 | 20826.884 |
|  |  | ICA + PNM vs SIMPLE | 1891.577 | 716.894 | 0.164 | -312.215 | 4095.368 |
|  |  | Phase-Randomised vs SIMPLE | -39372.209^*^ | 4695.648 | 0.000 | -53807.014 | -24937.404 |
|  | Phase-Randomised vs SIMPLE | PNM-only vs SIMPLE | -17687.112^*^ | 1881.573 | 0.000 | -23471.221 | -11903.004 |
|  |  | SIMPLE+PNM vs SIMPLE | 784.537 | 2248.681 | 1.000 | -6128.093 | 7697.167 |
|  |  | ICA + PNM vs SIMPLE | 56793.004^*^ | 5207.328 | 0.000 | 40785.251 | 72800.758 |
|  |  | ICA + PNM vs SIMPLE | 41263.786^*^ | 4952.932 | 0.000 | 26038.069 | 56489.503 |
|  |  | SIMPLE + ICA vs SIMPLE | 39372.209^*^ | 4695.648 | 0.000 | 24937.404 | 53807.014 |
| Based on estimated marginal means | | | | | | | |
| *. The mean difference is significant at the .05 level. | | | | | | | |
| b. Adjustment for multiple comparisons: Bonferroni. | | | | | | | |

**Supplementary Table 1E.)** Post-hoc paired *t*-tests between all contrasts of pipelines vs the SIMPLE pipeline, for each RSN separately, for connectivity changes within RSNs

| RSN | | | Mean Difference (I-J) | Std. Error | Sig.^b^ | 95% Confidence Interval for Difference^b^ | |
| --- | --- | --- | --- | --- | --- | --- | --- |
|  |  |  |  |  |  | Lower Bound | Upper Bound |
| rFPN | PNM-only vs SIMPLE | SIMPLE+PNM vs SIMPLE | 9180.844^*^ | 1564.241 | 0.000 | 4372.240 | 13989.448 |
|  |  | ICA + PNM vs SIMPLE | 92525.386^*^ | 9605.702 | 0.000 | 62996.671 | 122054.101 |
|  |  | ICA-only vs SIMPLE | 76469.433^*^ | 8715.373 | 0.000 | 49677.664 | 103261.203 |
|  |  | SIMPLE + ICA vs SIMPLE | 53273.817^*^ | 7088.070 | 0.000 | 31484.510 | 75063.124 |
|  |  | Phase-Randomised vs SIMPLE | 1015.747 | 1604.501 | 1.000 | -3916.621 | 5948.115 |
|  | SIMPLE+PNM vs SIMPLE | PNM-only vs SIMPLE | -9180.844^*^ | 1564.241 | 0.000 | -13989.448 | -4372.240 |
|  |  | ICA + PNM vs SIMPLE | 83344.542^*^ | 9047.376 | 0.000 | 55532.168 | 111156.916 |
|  |  | ICA-only vs SIMPLE | 67288.589^*^ | 8170.508 | 0.000 | 42171.778 | 92405.400 |
|  |  | SIMPLE + ICA vs SIMPLE | 44092.973^*^ | 6506.912 | 0.000 | 24090.195 | 64095.752 |
|  |  | Phase-Randomised vs SIMPLE | -8165.097^*^ | 2161.085 | 0.006 | -14808.450 | -1521.744 |
|  | ICA + PNM vs SIMPLE | PNM-only vs SIMPLE | -92525.386^*^ | 9605.702 | 0.000 | -122054.101 | -62996.671 |
|  |  | SIMPLE+PNM vs SIMPLE | -83344.542^*^ | 9047.376 | 0.000 | -111156.916 | -55532.168 |
|  |  | ICA-only vs SIMPLE | -16055.953^*^ | 1660.229 | 0.000 | -21159.634 | -10952.272 |
|  |  | SIMPLE + ICA vs SIMPLE | -39251.569^*^ | 3066.630 | 0.000 | -48678.641 | -29824.497 |
|  |  | Phase-Randomised vs SIMPLE | -91509.639^*^ | 9501.852 | 0.000 | -120719.110 | -62300.168 |
|  | ICA-only vs SIMPLE | PNM-only vs SIMPLE | -76469.433^*^ | 8715.373 | 0.000 | -103261.203 | -49677.664 |
|  |  | SIMPLE+PNM vs SIMPLE | -67288.589^*^ | 8170.508 | 0.000 | -92405.400 | -42171.778 |
|  |  | ICA + PNM vs SIMPLE | 16055.953^*^ | 1660.229 | 0.000 | 10952.272 | 21159.634 |
|  |  | SIMPLE + ICA vs SIMPLE | -23195.616^*^ | 2296.361 | 0.000 | -30254.817 | -16136.414 |
|  |  | Phase-Randomised vs SIMPLE | -75453.686^*^ | 8623.492 | 0.000 | -101963.007 | -48944.365 |
|  | SIMPLE + ICA vs SIMPLE | PNM-only vs SIMPLE | -53273.817^*^ | 7088.070 | 0.000 | -75063.124 | -31484.510 |
|  |  | SIMPLE+PNM vs SIMPLE | -44092.973^*^ | 6506.912 | 0.000 | -64095.752 | -24090.195 |
|  |  | ICA + PNM vs SIMPLE | 39251.569^*^ | 3066.630 | 0.000 | 29824.497 | 48678.641 |
|  |  | ICA-only vs SIMPLE | 23195.616^*^ | 2296.361 | 0.000 | 16136.414 | 30254.817 |
|  |  | Phase-Randomised vs SIMPLE | -52258.070^*^ | 6931.383 | 0.000 | -73565.710 | -30950.431 |
|  | Phase-Randomised vs SIMPLE | PNM-only vs SIMPLE | -1015.747 | 1604.501 | 1.000 | -5948.115 | 3916.621 |
|  |  | SIMPLE+PNM vs SIMPLE | 8165.097^*^ | 2161.085 | 0.006 | 1521.744 | 14808.450 |
|  |  | ICA + PNM vs SIMPLE | 91509.639^*^ | 9501.852 | 0.000 | 62300.168 | 120719.110 |
|  |  | ICA-only vs SIMPLE | 75453.686^*^ | 8623.492 | 0.000 | 48944.365 | 101963.007 |
|  |  | SIMPLE + ICA vs SIMPLE | 52258.070^*^ | 6931.383 | 0.000 | 30950.431 | 73565.710 |
| lFPN | PNM-only vs SIMPLE | SIMPLE+PNM vs SIMPLE | -116881.436^*^ | 12328.687 | 0.000 | -154780.829 | -78982.042 |
|  |  | ICA + PNM vs SIMPLE | 56326.905^*^ | 9473.098 | 0.000 | 27205.826 | 85447.983 |
|  |  | ICA-only vs SIMPLE | 54067.554^*^ | 9902.819 | 0.000 | 23625.478 | 84509.630 |
|  |  | SIMPLE + ICA vs SIMPLE | 24387.579 | 9507.791 | 0.198 | -4840.148 | 53615.305 |
|  |  | Phase-Randomised vs SIMPLE | -106383.428^*^ | 12156.406 | 0.000 | -143753.215 | -69013.641 |
|  | SIMPLE+PNM vs SIMPLE | PNM-only vs SIMPLE | 116881.436^*^ | 12328.687 | 0.000 | 78982.042 | 154780.829 |
|  |  | ICA + PNM vs SIMPLE | 173208.341^*^ | 12449.425 | 0.000 | 134937.790 | 211478.892 |
|  |  | ICA-only vs SIMPLE | 170948.990^*^ | 13633.443 | 0.000 | 129038.670 | 212859.310 |
|  |  | SIMPLE + ICA vs SIMPLE | 141269.014^*^ | 11638.477 | 0.000 | 105491.385 | 177046.644 |
|  |  | Phase-Randomised vs SIMPLE | 10498.008^*^ | 1020.224 | 0.000 | 7361.755 | 13634.260 |
|  | ICA + PNM vs SIMPLE | PNM-only vs SIMPLE | -56326.905^*^ | 9473.098 | 0.000 | -85447.983 | -27205.826 |
|  |  | SIMPLE+PNM vs SIMPLE | -173208.341^*^ | 12449.425 | 0.000 | -211478.892 | -134937.790 |
|  |  | ICA-only vs SIMPLE | -2259.351 | 2604.123 | 1.000 | -10264.637 | 5745.935 |
|  |  | SIMPLE + ICA vs SIMPLE | -31939.326^*^ | 3160.128 | 0.000 | -41653.820 | -22224.833 |
|  |  | Phase-Randomised vs SIMPLE | -162710.333^*^ | 12020.007 | 0.000 | -199660.818 | -125759.848 |
|  | ICA-only vs SIMPLE | PNM-only vs SIMPLE | -54067.554^*^ | 9902.819 | 0.000 | -84509.630 | -23625.478 |
|  |  | SIMPLE+PNM vs SIMPLE | -170948.990^*^ | 13633.443 | 0.000 | -212859.310 | -129038.670 |
|  |  | ICA + PNM vs SIMPLE | 2259.351 | 2604.123 | 1.000 | -5745.935 | 10264.637 |
|  |  | SIMPLE + ICA vs SIMPLE | -29679.975^*^ | 4040.727 | 0.000 | -42101.500 | -17258.450 |
|  |  | Phase-Randomised vs SIMPLE | -160450.982^*^ | 13216.245 | 0.000 | -201078.800 | -119823.164 |
|  | SIMPLE + ICA vs SIMPLE | PNM-only vs SIMPLE | -24387.579 | 9507.791 | 0.198 | -53615.305 | 4840.148 |
|  |  | SIMPLE+PNM vs SIMPLE | -141269.014^*^ | 11638.477 | 0.000 | -177046.644 | -105491.385 |
|  |  | ICA + PNM vs SIMPLE | 31939.326^*^ | 3160.128 | 0.000 | 22224.833 | 41653.820 |
|  |  | ICA-only vs SIMPLE | 29679.975^*^ | 4040.727 | 0.000 | 17258.450 | 42101.500 |
|  |  | Phase-Randomised vs SIMPLE | -130771.007^*^ | 11143.927 | 0.000 | -165028.350 | -96513.664 |
|  | Phase-Randomised vs SIMPLE | PNM-only vs SIMPLE | 106383.428^*^ | 12156.406 | 0.000 | 69013.641 | 143753.215 |
|  |  | SIMPLE+PNM vs SIMPLE | -10498.008^*^ | 1020.224 | 0.000 | -13634.260 | -7361.755 |
|  |  | ICA + PNM vs SIMPLE | 162710.333^*^ | 12020.007 | 0.000 | 125759.848 | 199660.818 |
|  |  | ICA-only vs SIMPLE | 160450.982^*^ | 13216.245 | 0.000 | 119823.164 | 201078.800 |
|  |  | SIMPLE + ICA vs SIMPLE | 130771.007^*^ | 11143.927 | 0.000 | 96513.664 | 165028.350 |
| SMN | PNM-only vs SIMPLE | SIMPLE+PNM vs SIMPLE | 40878.881^*^ | 6093.624 | 0.000 | 22146.583 | 59611.179 |
|  |  | ICA + PNM vs SIMPLE | 157316.646^*^ | 8804.222 | 0.000 | 130251.747 | 184381.546 |
|  |  | ICA-only vs SIMPLE | 141405.035^*^ | 8044.366 | 0.000 | 116675.995 | 166134.075 |
|  |  | SIMPLE + ICA vs SIMPLE | 102776.692^*^ | 7702.760 | 0.000 | 79097.778 | 126455.607 |
|  |  | Phase-Randomised vs SIMPLE | 48196.346^*^ | 6066.424 | 0.000 | 29547.661 | 66845.031 |
|  | SIMPLE+PNM vs SIMPLE | PNM-only vs SIMPLE | -40878.881^*^ | 6093.624 | 0.000 | -59611.179 | -22146.583 |
|  |  | ICA + PNM vs SIMPLE | 116437.765^*^ | 6243.212 | 0.000 | 97245.621 | 135629.910 |
|  |  | ICA-only vs SIMPLE | 100526.154^*^ | 5631.585 | 0.000 | 83214.201 | 117838.108 |
|  |  | SIMPLE + ICA vs SIMPLE | 61897.811^*^ | 4471.656 | 0.000 | 48151.574 | 75644.048 |
|  |  | Phase-Randomised vs SIMPLE | 7317.465 | 2426.708 | 0.059 | -142.435 | 14777.365 |
|  | ICA + PNM vs SIMPLE | PNM-only vs SIMPLE | -157316.646^*^ | 8804.222 | 0.000 | -184381.546 | -130251.747 |
|  |  | SIMPLE+PNM vs SIMPLE | -116437.765^*^ | 6243.212 | 0.000 | -135629.910 | -97245.621 |
|  |  | ICA-only vs SIMPLE | -15911.611^*^ | 2043.994 | 0.000 | -22195.016 | -9628.205 |
|  |  | SIMPLE + ICA vs SIMPLE | -54539.954^*^ | 2571.875 | 0.000 | -62446.108 | -46633.801 |
|  |  | Phase-Randomised vs SIMPLE | -109120.301^*^ | 6774.546 | 0.000 | -129945.809 | -88294.793 |
|  | ICA-only vs SIMPLE | PNM-only vs SIMPLE | -141405.035^*^ | 8044.366 | 0.000 | -166134.075 | -116675.995 |
|  |  | SIMPLE+PNM vs SIMPLE | -100526.154^*^ | 5631.585 | 0.000 | -117838.108 | -83214.201 |
|  |  | ICA + PNM vs SIMPLE | 15911.611^*^ | 2043.994 | 0.000 | 9628.205 | 22195.016 |
|  |  | SIMPLE + ICA vs SIMPLE | -38628.343^*^ | 2077.953 | 0.000 | -45016.139 | -32240.547 |
|  |  | Phase-Randomised vs SIMPLE | -93208.690^*^ | 6127.277 | 0.000 | -112044.441 | -74372.939 |
|  | SIMPLE + ICA vs SIMPLE | PNM-only vs SIMPLE | -102776.692^*^ | 7702.760 | 0.000 | -126455.607 | -79097.778 |
|  |  | SIMPLE+PNM vs SIMPLE | -61897.811^*^ | 4471.656 | 0.000 | -75644.048 | -48151.574 |
|  |  | ICA + PNM vs SIMPLE | 54539.954^*^ | 2571.875 | 0.000 | 46633.801 | 62446.108 |
|  |  | ICA-only vs SIMPLE | 38628.343^*^ | 2077.953 | 0.000 | 32240.547 | 45016.139 |
|  |  | Phase-Randomised vs SIMPLE | -54580.346^*^ | 4830.801 | 0.000 | -69430.623 | -39730.069 |
|  | Phase-Randomised vs SIMPLE | PNM-only vs SIMPLE | -48196.346^*^ | 6066.424 | 0.000 | -66845.031 | -29547.661 |
|  |  | SIMPLE+PNM vs SIMPLE | -7317.465 | 2426.708 | 0.059 | -14777.365 | 142.435 |
|  |  | ICA + PNM vs SIMPLE | 109120.301^*^ | 6774.546 | 0.000 | 88294.793 | 129945.809 |
|  |  | ICA-only vs SIMPLE | 93208.690^*^ | 6127.277 | 0.000 | 74372.939 | 112044.441 |
|  |  | SIMPLE + ICA vs SIMPLE | 54580.346^*^ | 4830.801 | 0.000 | 39730.069 | 69430.623 |
| VN | PNM-only vs SIMPLE | SIMPLE+PNM vs SIMPLE | -2717.480 | 1667.170 | 1.000 | -7842.497 | 2407.538 |
|  |  | ICA + PNM vs SIMPLE | 49486.960^*^ | 5055.349 | 0.000 | 33946.405 | 65027.515 |
|  |  | ICA + PNM vs SIMPLE | 41487.442^*^ | 5017.269 | 0.000 | 26063.946 | 56910.938 |
|  |  | SIMPLE + ICA vs SIMPLE | 7756.655 | 3932.529 | 0.807 | -4332.259 | 19845.570 |
|  |  | Phase-Randomised vs SIMPLE | 23304.720^*^ | 1553.457 | 0.000 | 18529.267 | 28080.173 |
|  | SIMPLE+PNM vs SIMPLE | PNM-only vs SIMPLE | 2717.480 | 1667.170 | 1.000 | -2407.538 | 7842.497 |
|  |  | ICA + PNM vs SIMPLE | 52204.440^*^ | 5252.562 | 0.000 | 36057.634 | 68351.246 |
|  |  | ICA + PNM vs SIMPLE | 44204.922^*^ | 5353.989 | 0.000 | 27746.322 | 60663.521 |
|  |  | SIMPLE + ICA vs SIMPLE | 10474.135 | 3476.349 | 0.059 | -212.446 | 21160.716 |
|  |  | Phase-Randomised vs SIMPLE | 26022.200^*^ | 2423.455 | 0.000 | 18572.301 | 33472.099 |
|  | ICA + PNM vs SIMPLE | PNM-only vs SIMPLE | -49486.960^*^ | 5055.349 | 0.000 | -65027.515 | -33946.405 |
|  |  | SIMPLE+PNM vs SIMPLE | -52204.440^*^ | 5252.562 | 0.000 | -68351.246 | -36057.634 |
|  |  | ICA + PNM vs SIMPLE | -7999.518^*^ | 1270.763 | 0.000 | -11905.948 | -4093.088 |
|  |  | SIMPLE + ICA vs SIMPLE | -41730.305^*^ | 3683.661 | 0.000 | -53054.181 | -30406.428 |
|  |  | Phase-Randomised vs SIMPLE | -26182.240^*^ | 4598.220 | 0.000 | -40317.545 | -12046.935 |
|  | ICA + PNM vs SIMPLE | PNM-only vs SIMPLE | -41487.442^*^ | 5017.269 | 0.000 | -56910.938 | -26063.946 |
|  |  | SIMPLE+PNM vs SIMPLE | -44204.922^*^ | 5353.989 | 0.000 | -60663.521 | -27746.322 |
|  |  | ICA + PNM vs SIMPLE | 7999.518^*^ | 1270.763 | 0.000 | 4093.088 | 11905.948 |
|  |  | SIMPLE + ICA vs SIMPLE | -33730.787^*^ | 3778.298 | 0.000 | -45345.583 | -22115.990 |
|  |  | Phase-Randomised vs SIMPLE | -18182.722^*^ | 4570.359 | 0.003 | -32232.380 | -4133.063 |
|  | SIMPLE + ICA vs SIMPLE | PNM-only vs SIMPLE | -7756.655 | 3932.529 | 0.807 | -19845.570 | 4332.259 |
|  |  | SIMPLE+PNM vs SIMPLE | -10474.135 | 3476.349 | 0.059 | -21160.716 | 212.446 |
|  |  | ICA + PNM vs SIMPLE | 41730.305^*^ | 3683.661 | 0.000 | 30406.428 | 53054.181 |
|  |  | ICA + PNM vs SIMPLE | 33730.787^*^ | 3778.298 | 0.000 | 22115.990 | 45345.583 |
|  |  | Phase-Randomised vs SIMPLE | 15548.065^*^ | 4334.074 | 0.011 | 2224.766 | 28871.364 |
|  | Phase-Randomised vs SIMPLE | PNM-only vs SIMPLE | -23304.720^*^ | 1553.457 | 0.000 | -28080.173 | -18529.267 |
|  |  | SIMPLE+PNM vs SIMPLE | -26022.200^*^ | 2423.455 | 0.000 | -33472.099 | -18572.301 |
|  |  | ICA + PNM vs SIMPLE | 26182.240^*^ | 4598.220 | 0.000 | 12046.935 | 40317.545 |
|  |  | ICA + PNM vs SIMPLE | 18182.722^*^ | 4570.359 | 0.003 | 4133.063 | 32232.380 |
|  |  | SIMPLE + ICA vs SIMPLE | -15548.065^*^ | 4334.074 | 0.011 | -28871.364 | -2224.766 |
| pDMN | PNM-only vs SIMPLE | SIMPLE+PNM vs SIMPLE | 41896.844^*^ | 3690.957 | 0.000 | 30550.540 | 53243.149 |
|  |  | ICA + PNM vs SIMPLE | 247029.498^*^ | 15470.188 | 0.000 | 199472.875 | 294586.121 |
|  |  | ICA + PNM vs SIMPLE | 210036.414^*^ | 15746.447 | 0.000 | 161630.548 | 258442.281 |
|  |  | SIMPLE + ICA vs SIMPLE | 175004.827^*^ | 15248.534 | 0.000 | 128129.586 | 221880.068 |
|  |  | Phase-Randomised vs SIMPLE | 42437.631^*^ | 4050.309 | 0.000 | 29986.649 | 54888.612 |
|  | SIMPLE+PNM vs SIMPLE | PNM-only vs SIMPLE | -41896.844^*^ | 3690.957 | 0.000 | -53243.149 | -30550.540 |
|  |  | ICA + PNM vs SIMPLE | 205132.653^*^ | 13450.076 | 0.000 | 163786.019 | 246479.288 |
|  |  | ICA + PNM vs SIMPLE | 168139.570^*^ | 13988.679 | 0.000 | 125137.225 | 211141.915 |
|  |  | SIMPLE + ICA vs SIMPLE | 133107.982^*^ | 13180.232 | 0.000 | 92590.872 | 173625.093 |
|  |  | Phase-Randomised vs SIMPLE | 540.786 | 4538.685 | 1.000 | -13411.503 | 14493.076 |
|  | ICA + PNM vs SIMPLE | PNM-only vs SIMPLE | -247029.498^*^ | 15470.188 | 0.000 | -294586.121 | -199472.875 |
|  |  | SIMPLE+PNM vs SIMPLE | -205132.653^*^ | 13450.076 | 0.000 | -246479.288 | -163786.019 |
|  |  | ICA + PNM vs SIMPLE | -36993.083^*^ | 3582.118 | 0.000 | -48004.806 | -25981.361 |
|  |  | SIMPLE + ICA vs SIMPLE | -72024.671^*^ | 3827.940 | 0.000 | -83792.071 | -60257.271 |
|  |  | Phase-Randomised vs SIMPLE | -204591.867^*^ | 13687.474 | 0.000 | -246668.282 | -162515.452 |
|  | ICA + PNM vs SIMPLE | PNM-only vs SIMPLE | -210036.414^*^ | 15746.447 | 0.000 | -258442.281 | -161630.548 |
|  |  | SIMPLE+PNM vs SIMPLE | -168139.570^*^ | 13988.679 | 0.000 | -211141.915 | -125137.225 |
|  |  | ICA + PNM vs SIMPLE | 36993.083^*^ | 3582.118 | 0.000 | 25981.361 | 48004.806 |
|  |  | SIMPLE + ICA vs SIMPLE | -35031.588^*^ | 4311.110 | 0.000 | -48284.292 | -21778.883 |
|  |  | Phase-Randomised vs SIMPLE | -167598.784^*^ | 14211.772 | 0.000 | -211286.932 | -123910.635 |
|  | SIMPLE + ICA vs SIMPLE | PNM-only vs SIMPLE | -175004.827^*^ | 15248.534 | 0.000 | -221880.068 | -128129.586 |
|  |  | SIMPLE+PNM vs SIMPLE | -133107.982^*^ | 13180.232 | 0.000 | -173625.093 | -92590.872 |
|  |  | ICA + PNM vs SIMPLE | 72024.671^*^ | 3827.940 | 0.000 | 60257.271 | 83792.071 |
|  |  | ICA + PNM vs SIMPLE | 35031.588^*^ | 4311.110 | 0.000 | 21778.883 | 48284.292 |
|  |  | Phase-Randomised vs SIMPLE | -132567.196^*^ | 13452.097 | 0.000 | -173920.041 | -91214.351 |
|  | Phase-Randomised vs SIMPLE | PNM-only vs SIMPLE | -42437.631^*^ | 4050.309 | 0.000 | -54888.612 | -29986.649 |
|  |  | SIMPLE+PNM vs SIMPLE | -540.786 | 4538.685 | 1.000 | -14493.076 | 13411.503 |
|  |  | ICA + PNM vs SIMPLE | 204591.867^*^ | 13687.474 | 0.000 | 162515.452 | 246668.282 |
|  |  | ICA + PNM vs SIMPLE | 167598.784^*^ | 14211.772 | 0.000 | 123910.635 | 211286.932 |
|  |  | SIMPLE + ICA vs SIMPLE | 132567.196^*^ | 13452.097 | 0.000 | 91214.351 | 173920.041 |
| aDMN | PNM-only vs SIMPLE | SIMPLE+PNM vs SIMPLE | 123463.297^*^ | 8871.263 | 0.000 | 96192.308 | 150734.286 |
|  |  | ICA + PNM vs SIMPLE | 206576.478^*^ | 12596.097 | 0.000 | 167855.045 | 245297.911 |
|  |  | ICA + PNM vs SIMPLE | 196608.228^*^ | 12689.129 | 0.000 | 157600.807 | 235615.648 |
|  |  | SIMPLE + ICA vs SIMPLE | 162511.375^*^ | 10456.308 | 0.000 | 130367.830 | 194654.920 |
|  |  | Phase-Randomised vs SIMPLE | 122152.919^*^ | 9130.457 | 0.000 | 94085.147 | 150220.691 |
|  | SIMPLE+PNM vs SIMPLE | PNM-only vs SIMPLE | -123463.297^*^ | 8871.263 | 0.000 | -150734.286 | -96192.308 |
|  |  | ICA + PNM vs SIMPLE | 83113.181^*^ | 9181.882 | 0.000 | 54887.325 | 111339.037 |
|  |  | ICA + PNM vs SIMPLE | 73144.931^*^ | 9325.737 | 0.000 | 44476.852 | 101813.010 |
|  |  | SIMPLE + ICA vs SIMPLE | 39048.078^*^ | 6744.311 | 0.000 | 18315.515 | 59780.641 |
|  |  | Phase-Randomised vs SIMPLE | -1310.378 | 2692.789 | 1.000 | -9588.231 | 6967.476 |
|  | ICA + PNM vs SIMPLE | PNM-only vs SIMPLE | -206576.478^*^ | 12596.097 | 0.000 | -245297.911 | -167855.045 |
|  |  | SIMPLE+PNM vs SIMPLE | -83113.181^*^ | 9181.882 | 0.000 | -111339.037 | -54887.325 |
|  |  | ICA + PNM vs SIMPLE | -9968.251^*^ | 2019.345 | 0.000 | -16175.881 | -3760.620 |
|  |  | SIMPLE + ICA vs SIMPLE | -44065.103^*^ | 3533.936 | 0.000 | -54928.712 | -33201.494 |
|  |  | Phase-Randomised vs SIMPLE | -84423.559^*^ | 8770.415 | 0.000 | -111384.532 | -57462.586 |
|  | ICA + PNM vs SIMPLE | PNM-only vs SIMPLE | -196608.228^*^ | 12689.129 | 0.000 | -235615.648 | -157600.807 |
|  |  | SIMPLE+PNM vs SIMPLE | -73144.931^*^ | 9325.737 | 0.000 | -101813.010 | -44476.852 |
|  |  | ICA + PNM vs SIMPLE | 9968.251^*^ | 2019.345 | 0.000 | 3760.620 | 16175.881 |
|  |  | SIMPLE + ICA vs SIMPLE | -34096.853^*^ | 3298.098 | 0.000 | -44235.477 | -23958.228 |
|  |  | Phase-Randomised vs SIMPLE | -74455.309^*^ | 8714.267 | 0.000 | -101243.679 | -47666.938 |
|  | SIMPLE + ICA vs SIMPLE | PNM-only vs SIMPLE | -162511.375^*^ | 10456.308 | 0.000 | -194654.920 | -130367.830 |
|  |  | SIMPLE+PNM vs SIMPLE | -39048.078^*^ | 6744.311 | 0.000 | -59780.641 | -18315.515 |
|  |  | ICA + PNM vs SIMPLE | 44065.103^*^ | 3533.936 | 0.000 | 33201.494 | 54928.712 |
|  |  | ICA + PNM vs SIMPLE | 34096.853^*^ | 3298.098 | 0.000 | 23958.228 | 44235.477 |
|  |  | Phase-Randomised vs SIMPLE | -40358.456^*^ | 6217.293 | 0.000 | -59470.925 | -21245.987 |
|  | Phase-Randomised vs SIMPLE | PNM-only vs SIMPLE | -122152.919^*^ | 9130.457 | 0.000 | -150220.691 | -94085.147 |
|  |  | SIMPLE+PNM vs SIMPLE | 1310.378 | 2692.789 | 1.000 | -6967.476 | 9588.231 |
|  |  | ICA + PNM vs SIMPLE | 84423.559^*^ | 8770.415 | 0.000 | 57462.586 | 111384.532 |
|  |  | ICA + PNM vs SIMPLE | 74455.309^*^ | 8714.267 | 0.000 | 47666.938 | 101243.679 |
|  |  | SIMPLE + ICA vs SIMPLE | 40358.456^*^ | 6217.293 | 0.000 | 21245.987 | 59470.925 |
| Based on estimated marginal means | | | | | | | |
| *. The mean difference is significant at the .05 level. | | | | | | | |
| b. Adjustment for multiple comparisons: Bonferroni. | | | | | | | |

**Supplementary Table 1F.)** Post-hoc paired *t*-tests between contrasts of pipelines vs the SIMPLE pipeline, for each RSN separately, for connectivity changes between RSNs and the rest of the brain

# Supplementary Table 2: KETAMINE - Results from ANOVA on Dual Regression Quantification

| (I) Pipeline | | Mean Difference (I-J) | Std. Error | Sig.^b^ | 95% Confidence Interval for Difference^b^ | |
| --- | --- | --- | --- | --- | --- | --- |
|  |  |  |  |  | Lower Bound | Upper Bound |
| SIMPLE | PNM-only | 694.918 | 631.353 | 1.000 | -1449.150 | 2838.987 |
|  | SIMPLE + PNM | 523.808 | 347.284 | 1.000 | -655.565 | 1703.180 |
|  | ICA + PNM | 13978.009^*^ | 1655.365 | 0.000 | 8356.409 | 19599.609 |
|  | ICA-only | 18541.378^*^ | 2023.810 | 0.000 | 11668.542 | 25414.214 |
|  | SIMPLE + ICA | 18084.277^*^ | 1910.685 | 0.000 | 11595.613 | 24572.940 |
|  | Phase-Randomised | -12213.067^*^ | 924.325 | 0.000 | -15352.063 | -9074.071 |
| PNM-only | SIMPLE | -694.918 | 631.353 | 1.000 | -2838.987 | 1449.150 |
|  | PNM-only | -171.110 | 650.844 | 1.000 | -2381.371 | 2039.150 |
|  | ICA + PNM | 13283.091^*^ | 1851.015 | 0.000 | 6997.065 | 19569.116 |
|  | ICA-only | 17846.460^*^ | 2215.896 | 0.000 | 10321.304 | 25371.616 |
|  | SIMPLE + ICA | 17389.358^*^ | 2085.595 | 0.000 | 10306.704 | 24472.013 |
|  | Phase-Randomised | -12907.985^*^ | 1068.412 | 0.000 | -16536.299 | -9279.671 |
| SIMPLE + PNM | SIMPLE | -523.808 | 347.284 | 1.000 | -1703.180 | 655.565 |
|  | PNM-only | 171.110 | 650.844 | 1.000 | -2039.150 | 2381.371 |
|  | ICA + PNM | 13454.201^*^ | 1649.971 | 0.000 | 7850.920 | 19057.483 |
|  | ICA-only | 18017.570^*^ | 2019.527 | 0.000 | 11159.280 | 24875.861 |
|  | SIMPLE + ICA | 17560.469^*^ | 1890.159 | 0.000 | 11141.511 | 23979.426 |
|  | Phase-Randomised | -12736.875^*^ | 990.336 | 0.000 | -16100.045 | -9373.705 |
| ICA + PNM | SIMPLE | -13978.009^*^ | 1655.365 | 0.000 | -19599.609 | -8356.409 |
|  | PNM-only | -13283.091^*^ | 1851.015 | 0.000 | -19569.116 | -6997.065 |
|  | SIMPLE + PNM | -13454.201^*^ | 1649.971 | 0.000 | -19057.483 | -7850.920 |
|  | ICA-only | 4563.369^*^ | 651.975 | 0.000 | 2349.269 | 6777.469 |
|  | SIMPLE + ICA | 4106.267^*^ | 649.536 | 0.000 | 1900.450 | 6312.085 |
|  | Phase-Randomised | -26191.076^*^ | 1959.270 | 0.000 | -32844.734 | -19537.418 |
| ICA-only | SIMPLE | -18541.378^*^ | 2023.810 | 0.000 | -25414.214 | -11668.542 |
|  | PNM-only | -17846.460^*^ | 2215.896 | 0.000 | -25371.616 | -10321.304 |
|  | SIMPLE + PNM | -18017.570^*^ | 2019.527 | 0.000 | -24875.861 | -11159.280 |
|  | ICA + PNM | -4563.369^*^ | 651.975 | 0.000 | -6777.469 | -2349.269 |
|  | SIMPLE + ICA | -457.102 | 562.979 | 1.000 | -2368.972 | 1454.769 |
|  | Phase-Randomised | -30754.445^*^ | 2396.014 | 0.000 | -38891.280 | -22617.610 |
| SIMPLE + ICA | SIMPLE | -18084.277^*^ | 1910.685 | 0.000 | -24572.940 | -11595.613 |
|  | PNM-only | -17389.358^*^ | 2085.595 | 0.000 | -24472.013 | -10306.704 |
|  | SIMPLE + PNM | -17560.469^*^ | 1890.159 | 0.000 | -23979.426 | -11141.511 |
|  | ICA + PNM | -4106.267^*^ | 649.536 | 0.000 | -6312.085 | -1900.450 |
|  | ICA-only | 457.102 | 562.979 | 1.000 | -1454.769 | 2368.972 |
|  | Phase-Randomised | -30297.344^*^ | 2291.898 | 0.000 | -38080.603 | -22514.084 |
| 7 | SIMPLE | 12213.067^*^ | 924.325 | 0.000 | 9074.071 | 15352.063 |
|  | PNM-only | 12907.985^*^ | 1068.412 | 0.000 | 9279.671 | 16536.299 |
|  | SIMPLE + PNM | 12736.875^*^ | 990.336 | 0.000 | 9373.705 | 16100.045 |
|  | ICA + PNM | 26191.076^*^ | 1959.270 | 0.000 | 19537.418 | 32844.734 |
|  | ICA-only | 30754.445^*^ | 2396.014 | 0.000 | 22617.610 | 38891.280 |
|  | SIMPLE + ICA | 30297.344^*^ | 2291.898 | 0.000 | 22514.084 | 38080.603 |
| Based on estimated marginal means | | | | | | |
| *. The mean difference is significant at the .05 level. | | | | | | |
| b. Adjustment for multiple comparisons: Bonferroni. | | | | | | |

**Supplementary Table 2A.)** Post-hoc paired *t*-tests between drug modulations in connectivity between pre-processing pipelines, across RSNs, for connectivity changes within RSNs

| (I) Pipeline | | Mean Difference (I-J) | Std. Error | Sig.^b^ | 95% Confidence Interval for Difference^b^ | |
| --- | --- | --- | --- | --- | --- | --- |
|  |  |  |  |  | Lower Bound | Upper Bound |
| SIMPLE | PNM-only | 2798.782^*^ | 349.739 | 0.000 | 1611.072 | 3986.492 |
|  | SIMPLE + PNM | 328.669^*^ | 59.517 | 0.000 | 126.551 | 530.786 |
|  | ICA + PNM | 3385.028^*^ | 301.501 | 0.000 | 2361.134 | 4408.922 |
|  | ICA-only | 5897.667^*^ | 476.570 | 0.000 | 4279.241 | 7516.093 |
|  | SIMPLE + ICA | 4811.692^*^ | 383.236 | 0.000 | 3510.227 | 6113.157 |
|  | Phase-Randomised | -3384.515^*^ | 181.803 | 0.000 | -4001.917 | -2767.114 |
| PNM-only | SIMPLE | -2798.782^*^ | 349.739 | 0.000 | -3986.492 | -1611.072 |
|  | PNM-only | -2470.114^*^ | 342.968 | 0.000 | -3634.830 | -1305.398 |
|  | ICA + PNM | 586.245 | 528.324 | 1.000 | -1207.935 | 2380.426 |
|  | ICA-only | 3098.885^*^ | 648.566 | 0.002 | 896.361 | 5301.408 |
|  | SIMPLE + ICA | 2012.909^*^ | 591.119 | 0.049 | 5.475 | 4020.344 |
|  | Phase-Randomised | -6183.298^*^ | 459.304 | 0.000 | -7743.089 | -4623.507 |
| SIMPLE + PNM | SIMPLE | -328.669^*^ | 59.517 | 0.000 | -530.786 | -126.551 |
|  | PNM-only | 2470.114^*^ | 342.968 | 0.000 | 1305.398 | 3634.830 |
|  | ICA + PNM | 3056.359^*^ | 287.992 | 0.000 | 2078.342 | 4034.376 |
|  | ICA-only | 5568.998^*^ | 465.116 | 0.000 | 3989.470 | 7148.526 |
|  | SIMPLE + ICA | 4483.023^*^ | 371.941 | 0.000 | 3219.916 | 5746.130 |
|  | Phase-Randomised | -3713.184^*^ | 196.133 | 0.000 | -4379.249 | -3047.119 |
| ICA + PNM | SIMPLE | -3385.028^*^ | 301.501 | 0.000 | -4408.922 | -2361.134 |
|  | PNM-only | -586.245 | 528.324 | 1.000 | -2380.426 | 1207.935 |
|  | SIMPLE + PNM | -3056.359^*^ | 287.992 | 0.000 | -4034.376 | -2078.342 |
|  | ICA-only | 2512.639^*^ | 222.852 | 0.000 | 1755.836 | 3269.442 |
|  | SIMPLE + ICA | 1426.664^*^ | 198.467 | 0.000 | 752.674 | 2100.654 |
|  | Phase-Randomised | -6769.543^*^ | 341.149 | 0.000 | -7928.080 | -5611.006 |
| ICA-only | SIMPLE | -5897.667^*^ | 476.570 | 0.000 | -7516.093 | -4279.241 |
|  | PNM-only | -3098.885^*^ | 648.566 | 0.002 | -5301.408 | -896.361 |
|  | SIMPLE + PNM | -5568.998^*^ | 465.116 | 0.000 | -7148.526 | -3989.470 |
|  | ICA + PNM | -2512.639^*^ | 222.852 | 0.000 | -3269.442 | -1755.836 |
|  | SIMPLE + ICA | -1085.975^*^ | 208.632 | 0.001 | -1794.485 | -377.465 |
|  | Phase-Randomised | -9282.182^*^ | 523.385 | 0.000 | -11059.592 | -7504.773 |
| SIMPLE + ICA | SIMPLE | -4811.692^*^ | 383.236 | 0.000 | -6113.157 | -3510.227 |
|  | PNM-only | -2012.909^*^ | 591.119 | 0.049 | -4020.344 | -5.475 |
|  | SIMPLE + PNM | -4483.023^*^ | 371.941 | 0.000 | -5746.130 | -3219.916 |
|  | ICA + PNM | -1426.664^*^ | 198.467 | 0.000 | -2100.654 | -752.674 |
|  | ICA-only | 1085.975^*^ | 208.632 | 0.001 | 377.465 | 1794.485 |
|  | Phase-Randomised | -8196.207^*^ | 419.114 | 0.000 | -9619.513 | -6772.901 |
| 7 | SIMPLE | 3384.515^*^ | 181.803 | 0.000 | 2767.114 | 4001.917 |
|  | PNM-only | 6183.298^*^ | 459.304 | 0.000 | 4623.507 | 7743.089 |
|  | SIMPLE + PNM | 3713.184^*^ | 196.133 | 0.000 | 3047.119 | 4379.249 |
|  | ICA + PNM | 6769.543^*^ | 341.149 | 0.000 | 5611.006 | 7928.080 |
|  | ICA-only | 9282.182^*^ | 523.385 | 0.000 | 7504.773 | 11059.592 |
|  | SIMPLE + ICA | 8196.207^*^ | 419.114 | 0.000 | 6772.901 | 9619.513 |
| Based on estimated marginal means | | | | | | |
| *. The mean difference is significant at the .05 level. | | | | | | |
| b. Adjustment for multiple comparisons: Bonferroni. | | | | | | |

**Supplementary Table 2B.)** Post-hoc paired *t*-tests between drug modulations in connectivity between pre-processing pipelines, across RSNs, for connectivity changes between RSNs and the rest of the brain

| Pipeline | Mean | Std. Error | 95% Confidence Interval | |
| --- | --- | --- | --- | --- |
|  |  |  | Lower Bound | Upper Bound |
| SIMPLE | -14930.824 | 1106.254 | -17214.019 | -12647.629 |
| PNM-only | -15625.742 | 1210.479 | -18124.047 | -13127.436 |
| SIMPLE + PNM | -15454.631 | 1162.939 | -17854.820 | -13054.442 |
| ICA + PNM | -28908.833 | 2079.302 | -33200.301 | -24617.364 |
| ICA-only | -33472.202 | 2515.476 | -38663.889 | -28280.514 |
| SIMPLE + ICA | -33015.100 | 2416.758 | -38003.043 | -28027.157 |
| Phase-Randomised | -2717.757 | 219.211 | -3170.186 | -2265.327 |

**Supplementary Table 2C.)** Average changes in connectivity within RSNS after drug administration, across RSNs, for each pipeline

| Pipeline | Mean | Std. Error | 95% Confidence Interval | |
| --- | --- | --- | --- | --- |
|  |  |  | Lower Bound | Upper Bound |
| SIMPLE | -3898.721 | 203.990 | -4319.735 | -3477.706 |
| PNM-only | -6697.503 | 477.698 | -7683.423 | -5711.582 |
| SIMPLE + PNM | -4227.389 | 219.159 | -4679.712 | -3775.067 |
| ICA + PNM | -7283.748 | 357.808 | -8022.229 | -6545.268 |
| ICA-only | -9796.388 | 539.848 | -10910.580 | -8682.195 |
| SIMPLE + ICA | -8710.412 | 436.476 | -9611.255 | -7809.570 |
| Phase-Randomised | -514.205 | 38.577 | -593.824 | -434.587 |

**Supplementary Table 2D.)** Average changes in connectivity between RSNS and the rest of the brain after drug administration, across RSNs, for each pipeline

| RSN | | | Mean Difference (I-J) | Std. Error | Sig.^b^ | 95% Confidence Interval for Difference^b^ | |
| --- | --- | --- | --- | --- | --- | --- | --- |
|  |  |  |  |  |  | Lower Bound | Upper Bound |
| rFPN | SIMPLE | PNM-only | 2949.630 | 1123.247 | 0.311 | -864.905 | 6764.166 |
|  |  | SIMPLE + PNM | -415.365 | 716.307 | 1.000 | -2847.934 | 2017.204 |
|  |  | ICA + PNM | 18642.202^*^ | 3334.244 | 0.000 | 7319.148 | 29965.256 |
|  |  | ICA-only | 23180.250^*^ | 4004.439 | 0.000 | 9581.223 | 36779.277 |
|  |  | SIMPLE + ICA | 27205.161^*^ | 4135.093 | 0.000 | 13162.434 | 41247.889 |
|  |  | Phase-Randomised | -18882.708^*^ | 1829.059 | 0.000 | -25094.172 | -12671.244 |
|  | PNM-only | SIMPLE | -2949.630 | 1123.247 | 0.311 | -6764.166 | 864.905 |
|  |  | SIMPLE + PNM | -3364.995 | 1315.781 | 0.363 | -7833.371 | 1103.381 |
|  |  | ICA + PNM | 15692.572^*^ | 3334.308 | 0.002 | 4369.301 | 27015.842 |
|  |  | ICA-only | 20230.620^*^ | 4000.986 | 0.001 | 6643.319 | 33817.921 |
|  |  | SIMPLE + ICA | 24255.531^*^ | 4191.287 | 0.000 | 10021.970 | 38489.092 |
|  |  | Phase-Randomised | -21832.338^*^ | 1991.448 | 0.000 | -28595.274 | -15069.403 |
|  | SIMPLE + PNM | SIMPLE | 415.365 | 716.307 | 1.000 | -2017.204 | 2847.934 |
|  |  | PNM-only | 3364.995 | 1315.781 | 0.363 | -1103.381 | 7833.371 |
|  |  | ICA + PNM | 19057.567^*^ | 3419.654 | 0.000 | 7444.462 | 30670.671 |
|  |  | ICA-only | 23595.615^*^ | 4147.401 | 0.000 | 9511.089 | 37680.140 |
|  |  | SIMPLE + ICA | 27620.526^*^ | 4211.578 | 0.000 | 13318.056 | 41922.996 |
|  |  | Phase-Randomised | -18467.344^*^ | 1717.409 | 0.000 | -24299.645 | -12635.042 |
|  | ICA + PNM | SIMPLE | -18642.202^*^ | 3334.244 | 0.000 | -29965.256 | -7319.148 |
|  |  | PNM-only | -15692.572^*^ | 3334.308 | 0.002 | -27015.842 | -4369.301 |
|  |  | SIMPLE + PNM | -19057.567^*^ | 3419.654 | 0.000 | -30670.671 | -7444.462 |
|  |  | ICA-only | 4538.048^*^ | 1225.417 | 0.023 | 376.546 | 8699.550 |
|  |  | SIMPLE + ICA | 8562.959^*^ | 1513.372 | 0.000 | 3423.566 | 13702.352 |
|  |  | Phase-Randomised | -37524.910^*^ | 3955.112 | 0.000 | -50956.423 | -24093.397 |
|  | ICA-only | SIMPLE | -23180.250^*^ | 4004.439 | 0.000 | -36779.277 | -9581.223 |
|  |  | PNM-only | -20230.620^*^ | 4000.986 | 0.001 | -33817.921 | -6643.319 |
|  |  | SIMPLE + PNM | -23595.615^*^ | 4147.401 | 0.000 | -37680.140 | -9511.089 |
|  |  | ICA + PNM | -4538.048^*^ | 1225.417 | 0.023 | -8699.550 | -376.546 |
|  |  | SIMPLE + ICA | 4024.911 | 1475.707 | 0.247 | -986.574 | 9036.396 |
|  |  | Phase-Randomised | -42062.958^*^ | 4723.230 | 0.000 | -58102.991 | -26022.925 |
|  | SIMPLE + ICA | SIMPLE | -27205.161^*^ | 4135.093 | 0.000 | -41247.889 | -13162.434 |
|  |  | PNM-only | -24255.531^*^ | 4191.287 | 0.000 | -38489.092 | -10021.970 |
|  |  | SIMPLE + PNM | -27620.526^*^ | 4211.578 | 0.000 | -41922.996 | -13318.056 |
|  |  | ICA + PNM | -8562.959^*^ | 1513.372 | 0.000 | -13702.352 | -3423.566 |
|  |  | ICA-only | -4024.911 | 1475.707 | 0.247 | -9036.396 | 986.574 |
|  |  | Phase-Randomised | -46087.869^*^ | 4945.101 | 0.000 | -62881.376 | -29294.363 |
|  | Phase-Randomised | SIMPLE | 18882.708^*^ | 1829.059 | 0.000 | 12671.244 | 25094.172 |
|  |  | PNM-only | 21832.338^*^ | 1991.448 | 0.000 | 15069.403 | 28595.274 |
|  |  | SIMPLE + PNM | 18467.344^*^ | 1717.409 | 0.000 | 12635.042 | 24299.645 |
|  |  | ICA + PNM | 37524.910^*^ | 3955.112 | 0.000 | 24093.397 | 50956.423 |
|  |  | ICA-only | 42062.958^*^ | 4723.230 | 0.000 | 26022.925 | 58102.991 |
|  |  | SIMPLE + ICA | 46087.869^*^ | 4945.101 | 0.000 | 29294.363 | 62881.376 |
| lFPN | SIMPLE | PNM-only | -12899.184^*^ | 1480.144 | 0.000 | -17925.736 | -7872.631 |
|  |  | SIMPLE + PNM | -3295.618^*^ | 542.016 | 0.000 | -5136.299 | -1454.936 |
|  |  | ICA + PNM | 7401.101^*^ | 2006.574 | 0.024 | 586.799 | 14215.403 |
|  |  | ICA-only | 8531.275^*^ | 2190.834 | 0.014 | 1091.227 | 15971.322 |
|  |  | SIMPLE + ICA | 10115.877^*^ | 2240.758 | 0.003 | 2506.289 | 17725.465 |
|  |  | Phase-Randomised | -13489.430^*^ | 1520.840 | 0.000 | -18654.184 | -8324.677 |
|  | PNM-only | SIMPLE | 12899.184^*^ | 1480.144 | 0.000 | 7872.631 | 17925.736 |
|  |  | SIMPLE + PNM | 9603.566^*^ | 1165.407 | 0.000 | 5645.859 | 13561.273 |
|  |  | ICA + PNM | 20300.285^*^ | 2129.024 | 0.000 | 13070.143 | 27530.426 |
|  |  | ICA-only | 21430.458^*^ | 2445.831 | 0.000 | 13124.444 | 29736.473 |
|  |  | SIMPLE + ICA | 23015.061^*^ | 2562.968 | 0.000 | 14311.252 | 31718.870 |
|  |  | Phase-Randomised | -590.247 | 398.349 | 1.000 | -1943.035 | 762.542 |
|  | SIMPLE + PNM | SIMPLE | 3295.618^*^ | 542.016 | 0.000 | 1454.936 | 5136.299 |
|  |  | PNM-only | -9603.566^*^ | 1165.407 | 0.000 | -13561.273 | -5645.859 |
|  |  | ICA + PNM | 10696.719^*^ | 1804.071 | 0.000 | 4570.117 | 16823.321 |
|  |  | ICA-only | 11826.893^*^ | 2061.689 | 0.000 | 4825.420 | 18828.365 |
|  |  | SIMPLE + ICA | 13411.495^*^ | 2190.976 | 0.000 | 5970.967 | 20852.023 |
|  |  | Phase-Randomised | -10193.812^*^ | 1259.036 | 0.000 | -14469.485 | -5918.140 |
|  | ICA + PNM | SIMPLE | -7401.101^*^ | 2006.574 | 0.024 | -14215.403 | -586.799 |
|  |  | PNM-only | -20300.285^*^ | 2129.024 | 0.000 | -27530.426 | -13070.143 |
|  |  | SIMPLE + PNM | -10696.719^*^ | 1804.071 | 0.000 | -16823.321 | -4570.117 |
|  |  | ICA-only | 1130.174 | 818.654 | 1.000 | -1649.965 | 3910.313 |
|  |  | SIMPLE + ICA | 2714.776 | 1024.786 | 0.295 | -765.386 | 6194.938 |
|  |  | Phase-Randomised | -20890.531^*^ | 2266.348 | 0.000 | -28587.021 | -13194.041 |
|  | ICA-only | SIMPLE | -8531.275^*^ | 2190.834 | 0.014 | -15971.322 | -1091.227 |
|  |  | PNM-only | -21430.458^*^ | 2445.831 | 0.000 | -29736.473 | -13124.444 |
|  |  | SIMPLE + PNM | -11826.893^*^ | 2061.689 | 0.000 | -18828.365 | -4825.420 |
|  |  | ICA + PNM | -1130.174 | 818.654 | 1.000 | -3910.313 | 1649.965 |
|  |  | SIMPLE + ICA | 1584.602 | 907.501 | 1.000 | -1497.260 | 4666.465 |
|  |  | Phase-Randomised | -22020.705^*^ | 2560.686 | 0.000 | -30716.765 | -13324.645 |
|  | SIMPLE + ICA | SIMPLE | -10115.877^*^ | 2240.758 | 0.003 | -17725.465 | -2506.289 |
|  |  | PNM-only | -23015.061^*^ | 2562.968 | 0.000 | -31718.870 | -14311.252 |
|  |  | SIMPLE + PNM | -13411.495^*^ | 2190.976 | 0.000 | -20852.023 | -5970.967 |
|  |  | ICA + PNM | -2714.776 | 1024.786 | 0.295 | -6194.938 | 765.386 |
|  |  | ICA-only | -1584.602 | 907.501 | 1.000 | -4666.465 | 1497.260 |
|  |  | Phase-Randomised | -23605.307^*^ | 2638.836 | 0.000 | -32566.762 | -14643.852 |
|  | Phase-Randomised | SIMPLE | 13489.430^*^ | 1520.840 | 0.000 | 8324.677 | 18654.184 |
|  |  | PNM-only | 590.247 | 398.349 | 1.000 | -762.542 | 1943.035 |
|  |  | SIMPLE + PNM | 10193.812^*^ | 1259.036 | 0.000 | 5918.140 | 14469.485 |
|  |  | ICA + PNM | 20890.531^*^ | 2266.348 | 0.000 | 13194.041 | 28587.021 |
|  |  | ICA-only | 22020.705^*^ | 2560.686 | 0.000 | 13324.645 | 30716.765 |
|  |  | SIMPLE + ICA | 23605.307^*^ | 2638.836 | 0.000 | 14643.852 | 32566.762 |
| SMN | SIMPLE | PNM-only | 16009.028^*^ | 3232.906 | 0.001 | 5030.118 | 26987.938 |
|  |  | SIMPLE + PNM | 6209.396^*^ | 1182.075 | 0.000 | 2195.084 | 10223.708 |
|  |  | ICA + PNM | 17830.377^*^ | 3228.422 | 0.000 | 6866.694 | 28794.060 |
|  |  | ICA-only | 20715.195^*^ | 3837.925 | 0.000 | 7681.647 | 33748.742 |
|  |  | SIMPLE + ICA | 20239.762^*^ | 3221.667 | 0.000 | 9299.018 | 31180.506 |
|  |  | Phase-Randomised | -2670.349^*^ | 394.000 | 0.000 | -4008.369 | -1332.328 |
|  | PNM-only | SIMPLE | -16009.028^*^ | 3232.906 | 0.001 | -26987.938 | -5030.118 |
|  |  | SIMPLE + PNM | -9799.632^*^ | 2234.646 | 0.004 | -17388.465 | -2210.799 |
|  |  | ICA + PNM | 1821.349 | 3404.452 | 1.000 | -9740.131 | 13382.829 |
|  |  | ICA-only | 4706.167 | 3578.274 | 1.000 | -7445.609 | 16857.943 |
|  |  | SIMPLE + ICA | 4230.735 | 3280.967 | 1.000 | -6911.390 | 15372.859 |
|  |  | Phase-Randomised | -18679.376^*^ | 3548.835 | 0.000 | -30731.178 | -6627.575 |
|  | SIMPLE + PNM | SIMPLE | -6209.396^*^ | 1182.075 | 0.000 | -10223.708 | -2195.084 |
|  |  | PNM-only | 9799.632^*^ | 2234.646 | 0.004 | 2210.799 | 17388.465 |
|  |  | ICA + PNM | 11620.981^*^ | 2729.115 | 0.006 | 2352.940 | 20889.022 |
|  |  | ICA-only | 14505.799^*^ | 3226.707 | 0.003 | 3547.940 | 25463.658 |
|  |  | SIMPLE + ICA | 14030.366^*^ | 2662.362 | 0.000 | 4989.017 | 23071.716 |
|  |  | Phase-Randomised | -8879.745^*^ | 1547.050 | 0.000 | -14133.508 | -3625.981 |
|  | ICA + PNM | SIMPLE | -17830.377^*^ | 3228.422 | 0.000 | -28794.060 | -6866.694 |
|  |  | PNM-only | -1821.349 | 3404.452 | 1.000 | -13382.829 | 9740.131 |
|  |  | SIMPLE + PNM | -11620.981^*^ | 2729.115 | 0.006 | -20889.022 | -2352.940 |
|  |  | ICA-only | 2884.818 | 1158.519 | 0.422 | -1049.498 | 6819.134 |
|  |  | SIMPLE + ICA | 2409.385 | 1001.454 | 0.508 | -991.542 | 5810.313 |
|  |  | Phase-Randomised | -20500.725^*^ | 3462.043 | 0.000 | -32257.784 | -8743.667 |
|  | ICA-only | SIMPLE | -20715.195^*^ | 3837.925 | 0.000 | -33748.742 | -7681.647 |
|  |  | PNM-only | -4706.167 | 3578.274 | 1.000 | -16857.943 | 7445.609 |
|  |  | SIMPLE + PNM | -14505.799^*^ | 3226.707 | 0.003 | -25463.658 | -3547.940 |
|  |  | ICA + PNM | -2884.818 | 1158.519 | 0.422 | -6819.134 | 1049.498 |
|  |  | SIMPLE + ICA | -475.432 | 988.374 | 1.000 | -3831.939 | 2881.075 |
|  |  | Phase-Randomised | -23385.543^*^ | 4094.768 | 0.000 | -37291.328 | -9479.758 |
|  | SIMPLE + ICA | SIMPLE | -20239.762^*^ | 3221.667 | 0.000 | -31180.506 | -9299.018 |
|  |  | PNM-only | -4230.735 | 3280.967 | 1.000 | -15372.859 | 6911.390 |
|  |  | SIMPLE + PNM | -14030.366^*^ | 2662.362 | 0.000 | -23071.716 | -4989.017 |
|  |  | ICA + PNM | -2409.385 | 1001.454 | 0.508 | -5810.313 | 991.542 |
|  |  | ICA-only | 475.432 | 988.374 | 1.000 | -2881.075 | 3831.939 |
|  |  | Phase-Randomised | -22910.111^*^ | 3477.595 | 0.000 | -34719.984 | -11100.238 |
|  | Phase-Randomised | SIMPLE | 2670.349^*^ | 394.000 | 0.000 | 1332.328 | 4008.369 |
|  |  | PNM-only | 18679.376^*^ | 3548.835 | 0.000 | 6627.575 | 30731.178 |
|  |  | SIMPLE + PNM | 8879.745^*^ | 1547.050 | 0.000 | 3625.981 | 14133.508 |
|  |  | ICA + PNM | 20500.725^*^ | 3462.043 | 0.000 | 8743.667 | 32257.784 |
|  |  | ICA-only | 23385.543^*^ | 4094.768 | 0.000 | 9479.758 | 37291.328 |
|  |  | SIMPLE + ICA | 22910.111^*^ | 3477.595 | 0.000 | 11100.238 | 34719.984 |
| VN | SIMPLE | PNM-only | -7254.357^*^ | 1131.866 | 0.000 | -11098.162 | -3410.553 |
|  |  | SIMPLE + PNM | -7706.465^*^ | 1070.248 | 0.000 | -11341.013 | -4071.917 |
|  |  | ICA + PNM | 3239.495 | 1527.735 | 0.934 | -1948.673 | 8427.664 |
|  |  | ICA-only | 7121.924^*^ | 1888.296 | 0.020 | 709.294 | 13534.553 |
|  |  | SIMPLE + ICA | 4939.846 | 1681.993 | 0.151 | -772.181 | 10651.874 |
|  |  | Phase-Randomised | -8684.679^*^ | 1105.959 | 0.000 | -12440.504 | -4928.854 |
|  | PNM-only | SIMPLE | 7254.357^*^ | 1131.866 | 0.000 | 3410.553 | 11098.162 |
|  |  | SIMPLE + PNM | -452.108 | 337.329 | 1.000 | -1597.674 | 693.459 |
|  |  | ICA + PNM | 10493.852^*^ | 1918.210 | 0.000 | 3979.632 | 17008.073 |
|  |  | ICA-only | 14376.281^*^ | 2452.247 | 0.000 | 6048.477 | 22704.084 |
|  |  | SIMPLE + ICA | 12194.204^*^ | 2223.614 | 0.000 | 4642.838 | 19745.569 |
|  |  | Phase-Randomised | -1430.322 | 443.630 | 0.076 | -2936.884 | 76.241 |
|  | SIMPLE + PNM | SIMPLE | 7706.465^*^ | 1070.248 | 0.000 | 4071.917 | 11341.013 |
|  |  | PNM-only | 452.108 | 337.329 | 1.000 | -693.459 | 1597.674 |
|  |  | ICA + PNM | 10945.960^*^ | 1800.041 | 0.000 | 4833.042 | 17058.878 |
|  |  | ICA-only | 14828.388^*^ | 2347.236 | 0.000 | 6857.203 | 22799.574 |
|  |  | SIMPLE + ICA | 12646.311^*^ | 2095.658 | 0.000 | 5529.480 | 19763.143 |
|  |  | Phase-Randomised | -978.214 | 337.819 | 0.167 | -2125.442 | 169.014 |
|  | ICA + PNM | SIMPLE | -3239.495 | 1527.735 | 0.934 | -8427.664 | 1948.673 |
|  |  | PNM-only | -10493.852^*^ | 1918.210 | 0.000 | -17008.073 | -3979.632 |
|  |  | SIMPLE + PNM | -10945.960^*^ | 1800.041 | 0.000 | -17058.878 | -4833.042 |
|  |  | ICA-only | 3882.428^*^ | 771.329 | 0.001 | 1263.006 | 6501.851 |
|  |  | SIMPLE + ICA | 1700.351 | 725.923 | 0.584 | -764.876 | 4165.579 |
|  |  | Phase-Randomised | -11924.174^*^ | 1865.089 | 0.000 | -18257.996 | -5590.352 |
|  | ICA-only | SIMPLE | -7121.924^*^ | 1888.296 | 0.020 | -13534.553 | -709.294 |
|  |  | PNM-only | -14376.281^*^ | 2452.247 | 0.000 | -22704.084 | -6048.477 |
|  |  | SIMPLE + PNM | -14828.388^*^ | 2347.236 | 0.000 | -22799.574 | -6857.203 |
|  |  | ICA + PNM | -3882.428^*^ | 771.329 | 0.001 | -6501.851 | -1263.006 |
|  |  | SIMPLE + ICA | -2182.077 | 739.466 | 0.146 | -4693.295 | 329.141 |
|  |  | Phase-Randomised | -15806.602^*^ | 2373.498 | 0.000 | -23866.974 | -7746.231 |
|  | SIMPLE + ICA | SIMPLE | -4939.846 | 1681.993 | 0.151 | -10651.874 | 772.181 |
|  |  | PNM-only | -12194.204^*^ | 2223.614 | 0.000 | -19745.569 | -4642.838 |
|  |  | SIMPLE + PNM | -12646.311^*^ | 2095.658 | 0.000 | -19763.143 | -5529.480 |
|  |  | ICA + PNM | -1700.351 | 725.923 | 0.584 | -4165.579 | 764.876 |
|  |  | ICA-only | 2182.077 | 739.466 | 0.146 | -329.141 | 4693.295 |
|  |  | Phase-Randomised | -13624.525^*^ | 2135.672 | 0.000 | -20877.243 | -6371.808 |
|  | Phase-Randomised | SIMPLE | 8684.679^*^ | 1105.959 | 0.000 | 4928.854 | 12440.504 |
|  |  | PNM-only | 1430.322 | 443.630 | 0.076 | -76.241 | 2936.884 |
|  |  | SIMPLE + PNM | 978.214 | 337.819 | 0.167 | -169.014 | 2125.442 |
|  |  | ICA + PNM | 11924.174^*^ | 1865.089 | 0.000 | 5590.352 | 18257.996 |
|  |  | ICA-only | 15806.602^*^ | 2373.498 | 0.000 | 7746.231 | 23866.974 |
|  |  | SIMPLE + ICA | 13624.525^*^ | 2135.672 | 0.000 | 6371.808 | 20877.243 |
| pDMN | SIMPLE | PNM-only | -6908.151^*^ | 754.192 | 0.000 | -9469.379 | -4346.922 |
|  |  | SIMPLE + PNM | 6041.102^*^ | 503.759 | 0.000 | 4330.342 | 7751.863 |
|  |  | ICA + PNM | 27072.787^*^ | 2807.959 | 0.000 | 17536.991 | 36608.582 |
|  |  | ICA-only | 33543.009^*^ | 3519.100 | 0.000 | 21592.188 | 45493.831 |
|  |  | SIMPLE + ICA | 32977.180^*^ | 3490.597 | 0.000 | 21123.153 | 44831.206 |
|  |  | Phase-Randomised | -7649.314^*^ | 745.687 | 0.000 | -10181.657 | -5116.970 |
|  | PNM-only | SIMPLE | 6908.151^*^ | 754.192 | 0.000 | 4346.922 | 9469.379 |
|  |  | SIMPLE + PNM | 12949.253^*^ | 979.985 | 0.000 | 9621.235 | 16277.271 |
|  |  | ICA + PNM | 33980.937^*^ | 3087.787 | 0.000 | 23494.849 | 44467.026 |
|  |  | ICA-only | 40451.160^*^ | 3810.766 | 0.000 | 27509.842 | 53392.478 |
|  |  | SIMPLE + ICA | 39885.330^*^ | 3784.034 | 0.000 | 27034.796 | 52735.864 |
|  |  | Phase-Randomised | -741.163^*^ | 104.373 | 0.000 | -1095.611 | -386.715 |
|  | SIMPLE + PNM | SIMPLE | -6041.102^*^ | 503.759 | 0.000 | -7751.863 | -4330.342 |
|  |  | PNM-only | -12949.253^*^ | 979.985 | 0.000 | -16277.271 | -9621.235 |
|  |  | ICA + PNM | 21031.684^*^ | 2878.681 | 0.000 | 11255.719 | 30807.650 |
|  |  | ICA-only | 27501.907^*^ | 3557.441 | 0.000 | 15420.878 | 39582.935 |
|  |  | SIMPLE + ICA | 26936.077^*^ | 3537.847 | 0.000 | 14921.590 | 38950.564 |
|  |  | Phase-Randomised | -13690.416^*^ | 992.648 | 0.000 | -17061.437 | -10319.395 |
|  | ICA + PNM | SIMPLE | -27072.787^*^ | 2807.959 | 0.000 | -36608.582 | -17536.991 |
|  |  | PNM-only | -33980.937^*^ | 3087.787 | 0.000 | -44467.026 | -23494.849 |
|  |  | SIMPLE + PNM | -21031.684^*^ | 2878.681 | 0.000 | -30807.650 | -11255.719 |
|  |  | ICA-only | 6470.222^*^ | 1159.221 | 0.000 | 2533.522 | 10406.923 |
|  |  | SIMPLE + ICA | 5904.393^*^ | 1077.873 | 0.000 | 2243.950 | 9564.836 |
|  |  | Phase-Randomised | -34722.101^*^ | 3112.956 | 0.000 | -45293.663 | -24150.538 |
|  | ICA-only | SIMPLE | -33543.009^*^ | 3519.100 | 0.000 | -45493.831 | -21592.188 |
|  |  | PNM-only | -40451.160^*^ | 3810.766 | 0.000 | -53392.478 | -27509.842 |
|  |  | SIMPLE + PNM | -27501.907^*^ | 3557.441 | 0.000 | -39582.935 | -15420.878 |
|  |  | ICA + PNM | -6470.222^*^ | 1159.221 | 0.000 | -10406.923 | -2533.522 |
|  |  | SIMPLE + ICA | -565.830 | 1107.707 | 1.000 | -4327.591 | 3195.931 |
|  |  | Phase-Randomised | -41192.323^*^ | 3846.964 | 0.000 | -54256.567 | -28128.079 |
|  | SIMPLE + ICA | SIMPLE | -32977.180^*^ | 3490.597 | 0.000 | -44831.206 | -21123.153 |
|  |  | PNM-only | -39885.330^*^ | 3784.034 | 0.000 | -52735.864 | -27034.796 |
|  |  | SIMPLE + PNM | -26936.077^*^ | 3537.847 | 0.000 | -38950.564 | -14921.590 |
|  |  | ICA + PNM | -5904.393^*^ | 1077.873 | 0.000 | -9564.836 | -2243.950 |
|  |  | ICA-only | 565.830 | 1107.707 | 1.000 | -3195.931 | 4327.591 |
|  |  | Phase-Randomised | -40626.493^*^ | 3816.555 | 0.000 | -53587.468 | -27665.518 |
|  | Phase-Randomised | SIMPLE | 7649.314^*^ | 745.687 | 0.000 | 5116.970 | 10181.657 |
|  |  | PNM-only | 741.163^*^ | 104.373 | 0.000 | 386.715 | 1095.611 |
|  |  | SIMPLE + PNM | 13690.416^*^ | 992.648 | 0.000 | 10319.395 | 17061.437 |
|  |  | ICA + PNM | 34722.101^*^ | 3112.956 | 0.000 | 24150.538 | 45293.663 |
|  |  | ICA-only | 41192.323^*^ | 3846.964 | 0.000 | 28128.079 | 54256.567 |
|  |  | SIMPLE + ICA | 40626.493^*^ | 3816.555 | 0.000 | 27665.518 | 53587.468 |
| aDMN | SIMPLE | PNM-only | 12272.543^*^ | 2355.796 | 0.001 | 4272.287 | 20272.798 |
|  |  | SIMPLE + PNM | 2309.796 | 1068.898 | 0.859 | -1320.171 | 5939.762 |
|  |  | ICA + PNM | 9682.093 | 3035.799 | 0.083 | -627.445 | 19991.631 |
|  |  | ICA-only | 18156.616^*^ | 3872.106 | 0.002 | 5006.988 | 31306.244 |
|  |  | SIMPLE + ICA | 13027.832^*^ | 3640.619 | 0.032 | 664.331 | 25391.333 |
|  |  | Phase-Randomised | -21901.923^*^ | 2361.340 | 0.000 | -29921.006 | -13882.839 |
|  | PNM-only | SIMPLE | -12272.543^*^ | 2355.796 | 0.001 | -20272.798 | -4272.287 |
|  |  | SIMPLE + PNM | -9962.747^*^ | 1926.122 | 0.001 | -16503.835 | -3421.659 |
|  |  | ICA + PNM | -2590.450 | 3536.300 | 1.000 | -14599.684 | 9418.784 |
|  |  | ICA-only | 5884.073 | 4342.458 | 1.000 | -8862.863 | 20631.010 |
|  |  | SIMPLE + ICA | 755.290 | 4054.349 | 1.000 | -13013.232 | 14523.812 |
|  |  | Phase-Randomised | -34174.465^*^ | 3414.066 | 0.000 | -45768.593 | -22580.338 |
|  | SIMPLE + PNM | SIMPLE | -2309.796 | 1068.898 | 0.859 | -5939.762 | 1320.171 |
|  |  | PNM-only | 9962.747^*^ | 1926.122 | 0.001 | 3421.659 | 16503.835 |
|  |  | ICA + PNM | 7372.297 | 2807.762 | 0.311 | -2162.831 | 16907.425 |
|  |  | ICA-only | 15846.820^*^ | 3726.604 | 0.006 | 3191.317 | 28502.323 |
|  |  | SIMPLE + ICA | 10718.036 | 3481.774 | 0.108 | -1106.028 | 22542.101 |
|  |  | Phase-Randomised | -24211.719^*^ | 2545.854 | 0.000 | -32857.411 | -15566.027 |
|  | ICA + PNM | SIMPLE | -9682.093 | 3035.799 | 0.083 | -19991.631 | 627.445 |
|  |  | PNM-only | 2590.450 | 3536.300 | 1.000 | -9418.784 | 14599.684 |
|  |  | SIMPLE + PNM | -7372.297 | 2807.762 | 0.311 | -16907.425 | 2162.831 |
|  |  | ICA-only | 8474.523^*^ | 1728.142 | 0.001 | 2605.773 | 14343.273 |
|  |  | SIMPLE + ICA | 3345.739 | 1750.965 | 1.000 | -2600.518 | 9291.997 |
|  |  | Phase-Randomised | -31584.016^*^ | 3139.704 | 0.000 | -42246.415 | -20921.616 |
|  | ICA-only | SIMPLE | -18156.616^*^ | 3872.106 | 0.002 | -31306.244 | -5006.988 |
|  |  | PNM-only | -5884.073 | 4342.458 | 1.000 | -20631.010 | 8862.863 |
|  |  | SIMPLE + PNM | -15846.820^*^ | 3726.604 | 0.006 | -28502.323 | -3191.317 |
|  |  | ICA + PNM | -8474.523^*^ | 1728.142 | 0.001 | -14343.273 | -2605.773 |
|  |  | SIMPLE + ICA | -5128.784^*^ | 1165.112 | 0.004 | -9085.490 | -1172.077 |
|  |  | Phase-Randomised | -40058.539^*^ | 4275.142 | 0.000 | -54576.870 | -25540.208 |
|  | SIMPLE + ICA | SIMPLE | -13027.832^*^ | 3640.619 | 0.032 | -25391.333 | -664.331 |
|  |  | PNM-only | -755.290 | 4054.349 | 1.000 | -14523.812 | 13013.232 |
|  |  | SIMPLE + PNM | -10718.036 | 3481.774 | 0.108 | -22542.101 | 1106.028 |
|  |  | ICA + PNM | -3345.739 | 1750.965 | 1.000 | -9291.997 | 2600.518 |
|  |  | ICA-only | 5128.784^*^ | 1165.112 | 0.004 | 1172.077 | 9085.490 |
|  |  | Phase-Randomised | -34929.755^*^ | 3992.473 | 0.000 | -48488.146 | -21371.364 |
|  | Phase-Randomised | SIMPLE | 21901.923^*^ | 2361.340 | 0.000 | 13882.839 | 29921.006 |
|  |  | PNM-only | 34174.465^*^ | 3414.066 | 0.000 | 22580.338 | 45768.593 |
|  |  | SIMPLE + PNM | 24211.719^*^ | 2545.854 | 0.000 | 15566.027 | 32857.411 |
|  |  | ICA + PNM | 31584.016^*^ | 3139.704 | 0.000 | 20921.616 | 42246.415 |
|  |  | ICA-only | 40058.539^*^ | 4275.142 | 0.000 | 25540.208 | 54576.870 |
|  |  | SIMPLE + ICA | 34929.755^*^ | 3992.473 | 0.000 | 21371.364 | 48488.146 |
| Based on estimated marginal means | | | | | | | |
| *. The mean difference is significant at the .05 level. | | | | | | | |
| b. Adjustment for multiple comparisons: Bonferroni. | | | | | | | |

**Supplementary Table 2E.)** Post-hoc paired *t*-tests between all pipelines for each RSN separately, for connectivity changes after drug administration within RSNs

| RSN | | | Mean Difference (I-J) | Std. Error | Sig.^b^ | 95% Confidence Interval for Difference^b^ | |
| --- | --- | --- | --- | --- | --- | --- | --- |
|  |  |  |  |  |  | Lower Bound | Upper Bound |
| rFPN | SIMPLE | PNM-only | 1322.310^*^ | 286.444 | 0.002 | 349.549 | 2295.071 |
|  |  | SIMPLE + PNM | -515.048 | 171.077 | 0.127 | -1096.022 | 65.926 |
|  |  | ICA + PNM | 4970.385^*^ | 571.174 | 0.000 | 3030.686 | 6910.084 |
|  |  | ICA-only | 5817.608^*^ | 637.769 | 0.000 | 3651.752 | 7983.464 |
|  |  | SIMPLE + ICA | 12254.581^*^ | 962.355 | 0.000 | 8986.434 | 15522.728 |
|  |  | Phase-Randomised | -3483.143^*^ | 310.497 | 0.000 | -4537.586 | -2428.700 |
|  | PNM-only | SIMPLE | -1322.310^*^ | 286.444 | 0.002 | -2295.071 | -349.549 |
|  |  | SIMPLE + PNM | -1837.358^*^ | 289.073 | 0.000 | -2819.045 | -855.671 |
|  |  | ICA + PNM | 3648.075^*^ | 646.062 | 0.000 | 1454.056 | 5842.094 |
|  |  | ICA-only | 4495.298^*^ | 716.060 | 0.000 | 2063.566 | 6927.030 |
|  |  | SIMPLE + ICA | 10932.271^*^ | 1032.393 | 0.000 | 7426.276 | 14438.265 |
|  |  | Phase-Randomised | -4805.453^*^ | 406.849 | 0.000 | -6187.108 | -3423.798 |
|  | SIMPLE + PNM | SIMPLE | 515.048 | 171.077 | 0.127 | -65.926 | 1096.022 |
|  |  | PNM-only | 1837.358^*^ | 289.073 | 0.000 | 855.671 | 2819.045 |
|  |  | ICA + PNM | 5485.433^*^ | 593.871 | 0.000 | 3468.655 | 7502.211 |
|  |  | ICA-only | 6332.656^*^ | 674.958 | 0.000 | 4040.506 | 8624.806 |
|  |  | SIMPLE + ICA | 12769.629^*^ | 997.994 | 0.000 | 9380.452 | 16158.805 |
|  |  | Phase-Randomised | -2968.095^*^ | 275.932 | 0.000 | -3905.156 | -2031.034 |
|  | ICA + PNM | SIMPLE | -4970.385^*^ | 571.174 | 0.000 | -6910.084 | -3030.686 |
|  |  | PNM-only | -3648.075^*^ | 646.062 | 0.000 | -5842.094 | -1454.056 |
|  |  | SIMPLE + PNM | -5485.433^*^ | 593.871 | 0.000 | -7502.211 | -3468.655 |
|  |  | ICA-only | 847.223^*^ | 208.401 | 0.009 | 139.497 | 1554.949 |
|  |  | SIMPLE + ICA | 7284.196^*^ | 476.557 | 0.000 | 5665.813 | 8902.579 |
|  |  | Phase-Randomised | -8453.528^*^ | 614.200 | 0.000 | -10539.343 | -6367.713 |
|  | ICA-only | SIMPLE | -5817.608^*^ | 637.769 | 0.000 | -7983.464 | -3651.752 |
|  |  | PNM-only | -4495.298^*^ | 716.060 | 0.000 | -6927.030 | -2063.566 |
|  |  | SIMPLE + PNM | -6332.656^*^ | 674.958 | 0.000 | -8624.806 | -4040.506 |
|  |  | ICA + PNM | -847.223^*^ | 208.401 | 0.009 | -1554.949 | -139.497 |
|  |  | SIMPLE + ICA | 6436.973^*^ | 452.061 | 0.000 | 4901.780 | 7972.165 |
|  |  | Phase-Randomised | -9300.751^*^ | 683.410 | 0.000 | -11621.603 | -6979.899 |
|  | SIMPLE + ICA | SIMPLE | -12254.581^*^ | 962.355 | 0.000 | -15522.728 | -8986.434 |
|  |  | PNM-only | -10932.271^*^ | 1032.393 | 0.000 | -14438.265 | -7426.276 |
|  |  | SIMPLE + PNM | -12769.629^*^ | 997.994 | 0.000 | -16158.805 | -9380.452 |
|  |  | ICA + PNM | -7284.196^*^ | 476.557 | 0.000 | -8902.579 | -5665.813 |
|  |  | ICA-only | -6436.973^*^ | 452.061 | 0.000 | -7972.165 | -4901.780 |
|  |  | Phase-Randomised | -15737.724^*^ | 1005.157 | 0.000 | -19151.226 | -12324.222 |
|  | Phase-Randomised | SIMPLE | 3483.143^*^ | 310.497 | 0.000 | 2428.700 | 4537.586 |
|  |  | PNM-only | 4805.453^*^ | 406.849 | 0.000 | 3423.798 | 6187.108 |
|  |  | SIMPLE + PNM | 2968.095^*^ | 275.932 | 0.000 | 2031.034 | 3905.156 |
|  |  | ICA + PNM | 8453.528^*^ | 614.200 | 0.000 | 6367.713 | 10539.343 |
|  |  | ICA-only | 9300.751^*^ | 683.410 | 0.000 | 6979.899 | 11621.603 |
|  |  | SIMPLE + ICA | 15737.724^*^ | 1005.157 | 0.000 | 12324.222 | 19151.226 |
| lFPN | SIMPLE | PNM-only | -2959.830^*^ | 298.873 | 0.000 | -3974.798 | -1944.861 |
|  |  | SIMPLE + PNM | -243.552 | 177.345 | 1.000 | -845.814 | 358.709 |
|  |  | ICA + PNM | 653.083 | 345.927 | 1.000 | -521.682 | 1827.849 |
|  |  | ICA-only | 1537.058^*^ | 419.464 | 0.026 | 112.563 | 2961.554 |
|  |  | SIMPLE + ICA | 2069.604^*^ | 411.846 | 0.001 | 670.982 | 3468.227 |
|  |  | Phase-Randomised | -2811.731^*^ | 281.915 | 0.000 | -3769.112 | -1854.349 |
|  | PNM-only | SIMPLE | 2959.830^*^ | 298.873 | 0.000 | 1944.861 | 3974.798 |
|  |  | SIMPLE + PNM | 2716.277^*^ | 284.325 | 0.000 | 1750.712 | 3681.843 |
|  |  | ICA + PNM | 3612.913^*^ | 253.928 | 0.000 | 2750.576 | 4475.250 |
|  |  | ICA-only | 4496.888^*^ | 356.277 | 0.000 | 3286.975 | 5706.801 |
|  |  | SIMPLE + ICA | 5029.434^*^ | 379.922 | 0.000 | 3739.222 | 6319.646 |
|  |  | Phase-Randomised | 148.099 | 45.776 | 0.074 | -7.357 | 303.555 |
|  | SIMPLE + PNM | SIMPLE | 243.552 | 177.345 | 1.000 | -358.709 | 845.814 |
|  |  | PNM-only | -2716.277^*^ | 284.325 | 0.000 | -3681.843 | -1750.712 |
|  |  | ICA + PNM | 896.636 | 320.211 | 0.208 | -190.796 | 1984.067 |
|  |  | ICA-only | 1780.610^*^ | 405.405 | 0.004 | 403.859 | 3157.362 |
|  |  | SIMPLE + ICA | 2313.157^*^ | 402.287 | 0.000 | 946.995 | 3679.318 |
|  |  | Phase-Randomised | -2568.179^*^ | 268.756 | 0.000 | -3480.872 | -1655.485 |
|  | ICA + PNM | SIMPLE | -653.083 | 345.927 | 1.000 | -1827.849 | 521.682 |
|  |  | PNM-only | -3612.913^*^ | 253.928 | 0.000 | -4475.250 | -2750.576 |
|  |  | SIMPLE + PNM | -896.636 | 320.211 | 0.208 | -1984.067 | 190.796 |
|  |  | ICA-only | 883.975^*^ | 196.056 | 0.003 | 218.172 | 1549.777 |
|  |  | SIMPLE + ICA | 1416.521^*^ | 216.421 | 0.000 | 681.557 | 2151.485 |
|  |  | Phase-Randomised | -3464.814^*^ | 273.390 | 0.000 | -4393.244 | -2536.384 |
|  | ICA-only | SIMPLE | -1537.058^*^ | 419.464 | 0.026 | -2961.554 | -112.563 |
|  |  | PNM-only | -4496.888^*^ | 356.277 | 0.000 | -5706.801 | -3286.975 |
|  |  | SIMPLE + PNM | -1780.610^*^ | 405.405 | 0.004 | -3157.362 | -403.859 |
|  |  | ICA + PNM | -883.975^*^ | 196.056 | 0.003 | -1549.777 | -218.172 |
|  |  | SIMPLE + ICA | 532.546 | 160.785 | 0.061 | -13.477 | 1078.569 |
|  |  | Phase-Randomised | -4348.789^*^ | 374.121 | 0.000 | -5619.300 | -3078.278 |
|  | SIMPLE + ICA | SIMPLE | -2069.604^*^ | 411.846 | 0.001 | -3468.227 | -670.982 |
|  |  | PNM-only | -5029.434^*^ | 379.922 | 0.000 | -6319.646 | -3739.222 |
|  |  | SIMPLE + PNM | -2313.157^*^ | 402.287 | 0.000 | -3679.318 | -946.995 |
|  |  | ICA + PNM | -1416.521^*^ | 216.421 | 0.000 | -2151.485 | -681.557 |
|  |  | ICA-only | -532.546 | 160.785 | 0.061 | -1078.569 | 13.477 |
|  |  | Phase-Randomised | -4881.335^*^ | 393.343 | 0.000 | -6217.125 | -3545.545 |
|  | Phase-Randomised | SIMPLE | 2811.731^*^ | 281.915 | 0.000 | 1854.349 | 3769.112 |
|  |  | PNM-only | -148.099 | 45.776 | 0.074 | -303.555 | 7.357 |
|  |  | SIMPLE + PNM | 2568.179^*^ | 268.756 | 0.000 | 1655.485 | 3480.872 |
|  |  | ICA + PNM | 3464.814^*^ | 273.390 | 0.000 | 2536.384 | 4393.244 |
|  |  | ICA-only | 4348.789^*^ | 374.121 | 0.000 | 3078.278 | 5619.300 |
|  |  | SIMPLE + ICA | 4881.335^*^ | 393.343 | 0.000 | 3545.545 | 6217.125 |
| SMN | SIMPLE | PNM-only | 3693.219^*^ | 626.590 | 0.000 | 1565.327 | 5821.112 |
|  |  | SIMPLE + PNM | -795.981^*^ | 142.365 | 0.000 | -1279.453 | -312.510 |
|  |  | ICA + PNM | -403.814 | 170.479 | 0.551 | -982.760 | 175.131 |
|  |  | ICA-only | 547.404 | 215.495 | 0.377 | -184.415 | 1279.222 |
|  |  | SIMPLE + ICA | 831.352^*^ | 216.923 | 0.017 | 94.683 | 1568.020 |
|  |  | Phase-Randomised | -1498.231^*^ | 162.935 | 0.000 | -2051.555 | -944.907 |
|  | PNM-only | SIMPLE | -3693.219^*^ | 626.590 | 0.000 | -5821.112 | -1565.327 |
|  |  | SIMPLE + PNM | -4489.200^*^ | 626.303 | 0.000 | -6616.119 | -2362.282 |
|  |  | ICA + PNM | -4097.034^*^ | 695.778 | 0.000 | -6459.887 | -1734.181 |
|  |  | ICA-only | -3145.816^*^ | 714.578 | 0.004 | -5572.514 | -719.118 |
|  |  | SIMPLE + ICA | -2861.868^*^ | 703.123 | 0.009 | -5249.666 | -474.070 |
|  |  | Phase-Randomised | -5191.451^*^ | 695.456 | 0.000 | -7553.210 | -2829.691 |
|  | SIMPLE + PNM | SIMPLE | 795.981^*^ | 142.365 | 0.000 | 312.510 | 1279.453 |
|  |  | PNM-only | 4489.200^*^ | 626.303 | 0.000 | 2362.282 | 6616.119 |
|  |  | ICA + PNM | 392.167 | 123.793 | 0.087 | -28.233 | 812.567 |
|  |  | ICA-only | 1343.385^*^ | 185.140 | 0.000 | 714.652 | 1972.118 |
|  |  | SIMPLE + ICA | 1627.333^*^ | 190.781 | 0.000 | 979.444 | 2275.221 |
|  |  | Phase-Randomised | -702.250^*^ | 94.138 | 0.000 | -1021.941 | -382.559 |
|  | ICA + PNM | SIMPLE | 403.814 | 170.479 | 0.551 | -175.131 | 982.760 |
|  |  | PNM-only | 4097.034^*^ | 695.778 | 0.000 | 1734.181 | 6459.887 |
|  |  | SIMPLE + PNM | -392.167 | 123.793 | 0.087 | -812.567 | 28.233 |
|  |  | ICA-only | 951.218^*^ | 86.392 | 0.000 | 657.832 | 1244.604 |
|  |  | SIMPLE + ICA | 1235.166^*^ | 103.177 | 0.000 | 884.777 | 1585.555 |
|  |  | Phase-Randomised | -1094.417^*^ | 105.354 | 0.000 | -1452.198 | -736.635 |
|  | ICA-only | SIMPLE | -547.404 | 215.495 | 0.377 | -1279.222 | 184.415 |
|  |  | PNM-only | 3145.816^*^ | 714.578 | 0.004 | 719.118 | 5572.514 |
|  |  | SIMPLE + PNM | -1343.385^*^ | 185.140 | 0.000 | -1972.118 | -714.652 |
|  |  | ICA + PNM | -951.218^*^ | 86.392 | 0.000 | -1244.604 | -657.832 |
|  |  | SIMPLE + ICA | 283.948^*^ | 73.202 | 0.015 | 35.355 | 532.540 |
|  |  | Phase-Randomised | -2045.635^*^ | 174.762 | 0.000 | -2639.125 | -1452.145 |
|  | SIMPLE + ICA | SIMPLE | -831.352^*^ | 216.923 | 0.017 | -1568.020 | -94.683 |
|  |  | PNM-only | 2861.868^*^ | 703.123 | 0.009 | 474.070 | 5249.666 |
|  |  | SIMPLE + PNM | -1627.333^*^ | 190.781 | 0.000 | -2275.221 | -979.444 |
|  |  | ICA + PNM | -1235.166^*^ | 103.177 | 0.000 | -1585.555 | -884.777 |
|  |  | ICA-only | -283.948^*^ | 73.202 | 0.015 | -532.540 | -35.355 |
|  |  | Phase-Randomised | -2329.583^*^ | 178.460 | 0.000 | -2935.632 | -1723.533 |
|  | Phase-Randomised | SIMPLE | 1498.231^*^ | 162.935 | 0.000 | 944.907 | 2051.555 |
|  |  | PNM-only | 5191.451^*^ | 695.456 | 0.000 | 2829.691 | 7553.210 |
|  |  | SIMPLE + PNM | 702.250^*^ | 94.138 | 0.000 | 382.559 | 1021.941 |
|  |  | ICA + PNM | 1094.417^*^ | 105.354 | 0.000 | 736.635 | 1452.198 |
|  |  | ICA-only | 2045.635^*^ | 174.762 | 0.000 | 1452.145 | 2639.125 |
|  |  | SIMPLE + ICA | 2329.583^*^ | 178.460 | 0.000 | 1723.533 | 2935.632 |
| VN | SIMPLE | PNM-only | -1235.731^*^ | 153.238 | 0.000 | -1756.127 | -715.335 |
|  |  | SIMPLE + PNM | -1409.299^*^ | 144.343 | 0.000 | -1899.487 | -919.110 |
|  |  | ICA + PNM | -1313.650^*^ | 214.236 | 0.000 | -2041.192 | -586.109 |
|  |  | ICA-only | -122.854 | 211.532 | 1.000 | -841.216 | 595.507 |
|  |  | SIMPLE + ICA | -279.609 | 219.034 | 1.000 | -1023.444 | 464.226 |
|  |  | Phase-Randomised | -1695.668^*^ | 168.981 | 0.000 | -2269.527 | -1121.810 |
|  | PNM-only | SIMPLE | 1235.731^*^ | 153.238 | 0.000 | 715.335 | 1756.127 |
|  |  | SIMPLE + PNM | -173.568 | 95.081 | 1.000 | -496.460 | 149.325 |
|  |  | ICA + PNM | -77.920 | 155.090 | 1.000 | -604.603 | 448.764 |
|  |  | ICA-only | 1112.877^*^ | 216.647 | 0.001 | 377.145 | 1848.608 |
|  |  | SIMPLE + ICA | 956.122^*^ | 210.691 | 0.003 | 240.619 | 1671.624 |
|  |  | Phase-Randomised | -459.938^*^ | 131.208 | 0.038 | -905.518 | -14.357 |
|  | SIMPLE + PNM | SIMPLE | 1409.299^*^ | 144.343 | 0.000 | 919.110 | 1899.487 |
|  |  | PNM-only | 173.568 | 95.081 | 1.000 | -149.325 | 496.460 |
|  |  | ICA + PNM | 95.648 | 127.366 | 1.000 | -336.886 | 528.183 |
|  |  | ICA-only | 1286.445^*^ | 201.440 | 0.000 | 602.355 | 1970.534 |
|  |  | SIMPLE + ICA | 1129.690^*^ | 185.984 | 0.000 | 498.089 | 1761.290 |
|  |  | Phase-Randomised | -286.370 | 111.181 | 0.348 | -663.939 | 91.199 |
|  | ICA + PNM | SIMPLE | 1313.650^*^ | 214.236 | 0.000 | 586.109 | 2041.192 |
|  |  | PNM-only | 77.920 | 155.090 | 1.000 | -448.764 | 604.603 |
|  |  | SIMPLE + PNM | -95.648 | 127.366 | 1.000 | -528.183 | 336.886 |
|  |  | ICA-only | 1190.796^*^ | 145.469 | 0.000 | 696.784 | 1684.809 |
|  |  | SIMPLE + ICA | 1034.041^*^ | 127.811 | 0.000 | 599.998 | 1468.084 |
|  |  | Phase-Randomised | -382.018 | 113.398 | 0.053 | -767.117 | 3.081 |
|  | ICA-only | SIMPLE | 122.854 | 211.532 | 1.000 | -595.507 | 841.216 |
|  |  | PNM-only | -1112.877^*^ | 216.647 | 0.001 | -1848.608 | -377.145 |
|  |  | SIMPLE + PNM | -1286.445^*^ | 201.440 | 0.000 | -1970.534 | -602.355 |
|  |  | ICA + PNM | -1190.796^*^ | 145.469 | 0.000 | -1684.809 | -696.784 |
|  |  | SIMPLE + ICA | -156.755 | 65.796 | 0.535 | -380.198 | 66.689 |
|  |  | Phase-Randomised | -1572.814^*^ | 177.401 | 0.000 | -2175.265 | -970.363 |
|  | SIMPLE + ICA | SIMPLE | 279.609 | 219.034 | 1.000 | -464.226 | 1023.444 |
|  |  | PNM-only | -956.122^*^ | 210.691 | 0.003 | -1671.624 | -240.619 |
|  |  | SIMPLE + PNM | -1129.690^*^ | 185.984 | 0.000 | -1761.290 | -498.089 |
|  |  | ICA + PNM | -1034.041^*^ | 127.811 | 0.000 | -1468.084 | -599.998 |
|  |  | ICA-only | 156.755 | 65.796 | 0.535 | -66.689 | 380.198 |
|  |  | Phase-Randomised | -1416.059^*^ | 167.668 | 0.000 | -1985.457 | -846.662 |
|  | Phase-Randomised | SIMPLE | 1695.668^*^ | 168.981 | 0.000 | 1121.810 | 2269.527 |
|  |  | PNM-only | 459.938^*^ | 131.208 | 0.038 | 14.357 | 905.518 |
|  |  | SIMPLE + PNM | 286.370 | 111.181 | 0.348 | -91.199 | 663.939 |
|  |  | ICA + PNM | 382.018 | 113.398 | 0.053 | -3.081 | 767.117 |
|  |  | ICA-only | 1572.814^*^ | 177.401 | 0.000 | 970.363 | 2175.265 |
|  |  | SIMPLE + ICA | 1416.059^*^ | 167.668 | 0.000 | 846.662 | 1985.457 |
| pDMN | SIMPLE | PNM-only | -1010.220^*^ | 117.471 | 0.000 | -1409.150 | -611.290 |
|  |  | SIMPLE + PNM | 1068.752^*^ | 142.813 | 0.000 | 583.761 | 1553.742 |
|  |  | ICA + PNM | 4429.883^*^ | 324.788 | 0.000 | 3326.905 | 5532.861 |
|  |  | ICA-only | 7444.525^*^ | 580.560 | 0.000 | 5472.948 | 9416.101 |
|  |  | SIMPLE + ICA | 6854.317^*^ | 583.622 | 0.000 | 4872.345 | 8836.289 |
|  |  | Phase-Randomised | -723.017^*^ | 97.352 | 0.000 | -1053.622 | -392.412 |
|  | PNM-only | SIMPLE | 1010.220^*^ | 117.471 | 0.000 | 611.290 | 1409.150 |
|  |  | SIMPLE + PNM | 2078.972^*^ | 177.564 | 0.000 | 1475.966 | 2681.978 |
|  |  | ICA + PNM | 5440.103^*^ | 334.789 | 0.000 | 4303.162 | 6577.044 |
|  |  | ICA-only | 8454.745^*^ | 582.709 | 0.000 | 6475.872 | 10433.618 |
|  |  | SIMPLE + ICA | 7864.537^*^ | 575.554 | 0.000 | 5909.961 | 9819.113 |
|  |  | Phase-Randomised | 287.203^*^ | 32.621 | 0.000 | 176.424 | 397.983 |
|  | SIMPLE + PNM | SIMPLE | -1068.752^*^ | 142.813 | 0.000 | -1553.742 | -583.761 |
|  |  | PNM-only | -2078.972^*^ | 177.564 | 0.000 | -2681.978 | -1475.966 |
|  |  | ICA + PNM | 3361.131^*^ | 255.991 | 0.000 | 2491.789 | 4230.473 |
|  |  | ICA-only | 6375.773^*^ | 516.658 | 0.000 | 4621.209 | 8130.337 |
|  |  | SIMPLE + ICA | 5785.565^*^ | 520.225 | 0.000 | 4018.886 | 7552.245 |
|  |  | Phase-Randomised | -1791.769^*^ | 173.069 | 0.000 | -2379.507 | -1204.030 |
|  | ICA + PNM | SIMPLE | -4429.883^*^ | 324.788 | 0.000 | -5532.861 | -3326.905 |
|  |  | PNM-only | -5440.103^*^ | 334.789 | 0.000 | -6577.044 | -4303.162 |
|  |  | SIMPLE + PNM | -3361.131^*^ | 255.991 | 0.000 | -4230.473 | -2491.789 |
|  |  | ICA-only | 3014.642^*^ | 313.452 | 0.000 | 1950.164 | 4079.120 |
|  |  | SIMPLE + ICA | 2424.434^*^ | 336.491 | 0.000 | 1281.716 | 3567.153 |
|  |  | Phase-Randomised | -5152.900^*^ | 335.833 | 0.000 | -6293.386 | -4012.413 |
|  | ICA-only | SIMPLE | -7444.525^*^ | 580.560 | 0.000 | -9416.101 | -5472.948 |
|  |  | PNM-only | -8454.745^*^ | 582.709 | 0.000 | -10433.618 | -6475.872 |
|  |  | SIMPLE + PNM | -6375.773^*^ | 516.658 | 0.000 | -8130.337 | -4621.209 |
|  |  | ICA + PNM | -3014.642^*^ | 313.452 | 0.000 | -4079.120 | -1950.164 |
|  |  | SIMPLE + ICA | -590.208 | 200.327 | 0.148 | -1270.514 | 90.099 |
|  |  | Phase-Randomised | -8167.542^*^ | 582.702 | 0.000 | -10146.392 | -6188.691 |
|  | SIMPLE + ICA | SIMPLE | -6854.317^*^ | 583.622 | 0.000 | -8836.289 | -4872.345 |
|  |  | PNM-only | -7864.537^*^ | 575.554 | 0.000 | -9819.113 | -5909.961 |
|  |  | SIMPLE + PNM | -5785.565^*^ | 520.225 | 0.000 | -7552.245 | -4018.886 |
|  |  | ICA + PNM | -2424.434^*^ | 336.491 | 0.000 | -3567.153 | -1281.716 |
|  |  | ICA-only | 590.208 | 200.327 | 0.148 | -90.099 | 1270.514 |
|  |  | Phase-Randomised | -7577.334^*^ | 578.434 | 0.000 | -9541.688 | -5612.980 |
|  | Phase-Randomised | SIMPLE | 723.017^*^ | 97.352 | 0.000 | 392.412 | 1053.622 |
|  |  | PNM-only | -287.203^*^ | 32.621 | 0.000 | -397.983 | -176.424 |
|  |  | SIMPLE + PNM | 1791.769^*^ | 173.069 | 0.000 | 1204.030 | 2379.507 |
|  |  | ICA + PNM | 5152.900^*^ | 335.833 | 0.000 | 4012.413 | 6293.386 |
|  |  | ICA-only | 8167.542^*^ | 582.702 | 0.000 | 6188.691 | 10146.392 |
|  |  | SIMPLE + ICA | 7577.334^*^ | 578.434 | 0.000 | 5612.980 | 9541.688 |
| aDMN | SIMPLE | PNM-only | 16982.945^*^ | 2060.775 | 0.000 | 9984.578 | 23981.311 |
|  |  | SIMPLE + PNM | 3867.140^*^ | 398.845 | 0.000 | 2512.668 | 5221.611 |
|  |  | ICA + PNM | 11974.279^*^ | 1482.058 | 0.000 | 6941.226 | 17007.333 |
|  |  | ICA-only | 20162.261^*^ | 2385.561 | 0.000 | 12060.924 | 28263.598 |
|  |  | SIMPLE + ICA | 7139.905^*^ | 1416.479 | 0.001 | 2329.558 | 11950.253 |
|  |  | Phase-Randomised | -10095.302^*^ | 598.790 | 0.000 | -12128.787 | -8061.818 |
|  | PNM-only | SIMPLE | -16982.945^*^ | 2060.775 | 0.000 | -23981.311 | -9984.578 |
|  |  | SIMPLE + PNM | -13115.805^*^ | 1904.187 | 0.000 | -19582.400 | -6649.210 |
|  |  | ICA + PNM | -5008.665 | 2545.456 | 1.000 | -13653.004 | 3635.674 |
|  |  | ICA-only | 3179.316 | 3182.679 | 1.000 | -7629.025 | 13987.657 |
|  |  | SIMPLE + ICA | -9843.039^*^ | 2534.138 | 0.015 | -18448.941 | -1237.137 |
|  |  | Phase-Randomised | -27078.247^*^ | 2281.212 | 0.000 | -34825.217 | -19331.277 |
|  | SIMPLE + PNM | SIMPLE | -3867.140^*^ | 398.845 | 0.000 | -5221.611 | -2512.668 |
|  |  | PNM-only | 13115.805^*^ | 1904.187 | 0.000 | 6649.210 | 19582.400 |
|  |  | ICA + PNM | 8107.140^*^ | 1344.678 | 0.000 | 3540.629 | 12673.650 |
|  |  | ICA-only | 16295.121^*^ | 2267.060 | 0.000 | 8596.213 | 23994.029 |
|  |  | SIMPLE + ICA | 3272.766 | 1254.837 | 0.324 | -988.645 | 7534.177 |
|  |  | Phase-Randomised | -13962.442^*^ | 711.677 | 0.000 | -16379.290 | -11545.594 |
|  | ICA + PNM | SIMPLE | -11974.279^*^ | 1482.058 | 0.000 | -17007.333 | -6941.226 |
|  |  | PNM-only | 5008.665 | 2545.456 | 1.000 | -3635.674 | 13653.004 |
|  |  | SIMPLE + PNM | -8107.140^*^ | 1344.678 | 0.000 | -12673.650 | -3540.629 |
|  |  | ICA-only | 8187.981^*^ | 1157.359 | 0.000 | 4257.603 | 12118.359 |
|  |  | SIMPLE + ICA | -4834.374^*^ | 870.250 | 0.000 | -7789.732 | -1879.016 |
|  |  | Phase-Randomised | -22069.582^*^ | 1721.695 | 0.000 | -27916.438 | -16222.726 |
|  | ICA-only | SIMPLE | -20162.261^*^ | 2385.561 | 0.000 | -28263.598 | -12060.924 |
|  |  | PNM-only | -3179.316 | 3182.679 | 1.000 | -13987.657 | 7629.025 |
|  |  | SIMPLE + PNM | -16295.121^*^ | 2267.060 | 0.000 | -23994.029 | -8596.213 |
|  |  | ICA + PNM | -8187.981^*^ | 1157.359 | 0.000 | -12118.359 | -4257.603 |
|  |  | SIMPLE + ICA | -13022.355^*^ | 1330.079 | 0.000 | -17539.289 | -8505.421 |
|  |  | Phase-Randomised | -30257.563^*^ | 2622.377 | 0.000 | -39163.123 | -21352.003 |
|  | SIMPLE + ICA | SIMPLE | -7139.905^*^ | 1416.479 | 0.001 | -11950.253 | -2329.558 |
|  |  | PNM-only | 9843.039^*^ | 2534.138 | 0.015 | 1237.137 | 18448.941 |
|  |  | SIMPLE + PNM | -3272.766 | 1254.837 | 0.324 | -7534.177 | 988.645 |
|  |  | ICA + PNM | 4834.374^*^ | 870.250 | 0.000 | 1879.016 | 7789.732 |
|  |  | ICA-only | 13022.355^*^ | 1330.079 | 0.000 | 8505.421 | 17539.289 |
|  |  | Phase-Randomised | -17235.208^*^ | 1582.052 | 0.000 | -22607.837 | -11862.579 |
|  | Phase-Randomised | SIMPLE | 10095.302^*^ | 598.790 | 0.000 | 8061.818 | 12128.787 |
|  |  | PNM-only | 27078.247^*^ | 2281.212 | 0.000 | 19331.277 | 34825.217 |
|  |  | SIMPLE + PNM | 13962.442^*^ | 711.677 | 0.000 | 11545.594 | 16379.290 |
|  |  | ICA + PNM | 22069.582^*^ | 1721.695 | 0.000 | 16222.726 | 27916.438 |
|  |  | ICA-only | 30257.563^*^ | 2622.377 | 0.000 | 21352.003 | 39163.123 |
|  |  | SIMPLE + ICA | 17235.208^*^ | 1582.052 | 0.000 | 11862.579 | 22607.837 |
| Based on estimated marginal means | | | | | | | |
| *. The mean difference is significant at the .05 level. | | | | | | | |
| b. Adjustment for multiple comparisons: Bonferroni. | | | | | | | |

**Supplementary Table 2F.)** Post-hoc paired *t*-tests between all pipelines for each RSN separately, for connectivity changes after drug administration between RSNs and the rest of the brain

# Supplementary Table 3: Midazolam - Results from ANOVA on Dual Regression Quantification

| (I) Pipeline | | Mean Difference (I-J) | Std. Error | Sig.^b^ | 95% Confidence Interval for Difference^b^ | |
| --- | --- | --- | --- | --- | --- | --- |
|  |  |  |  |  | Lower Bound | Upper Bound |
| SIMPLE | PNM-only | 2036.754 | 918.289 | 0.762 | -1081.744 | 5155.252 |
|  | SIMPLE + PNM | 2283.498^*^ | 621.001 | 0.025 | 174.585 | 4392.410 |
|  | ICA + PNM | 20005.961^*^ | 3296.270 | 0.000 | 8811.866 | 31200.055 |
|  | ICA-only | 19400.563^*^ | 3309.757 | 0.000 | 8160.667 | 30640.459 |
|  | SIMPLE + ICA | 18889.607^*^ | 3257.892 | 0.000 | 7825.844 | 29953.370 |
|  | Phase-Randomised | 11683.408^*^ | 1779.632 | 0.000 | 5639.797 | 17727.018 |
| PNM-only | SIMPLE | -2036.754 | 918.289 | 0.762 | -5155.252 | 1081.744 |
|  | PNM-only | 246.743 | 687.571 | 1.000 | -2088.238 | 2581.725 |
|  | ICA + PNM | 17969.207^*^ | 2910.450 | 0.000 | 8085.353 | 27853.060 |
|  | ICA-only | 17363.808^*^ | 2946.583 | 0.000 | 7357.246 | 27370.371 |
|  | SIMPLE + ICA | 16852.853^*^ | 2886.050 | 0.000 | 7051.862 | 26653.844 |
|  | Phase-Randomised | 9646.654^*^ | 1577.924 | 0.000 | 4288.043 | 15005.264 |
| SIMPLE + PNM | SIMPLE | -2283.498^*^ | 621.001 | 0.025 | -4392.410 | -174.585 |
|  | PNM-only | -246.743 | 687.571 | 1.000 | -2581.725 | 2088.238 |
|  | ICA + PNM | 17722.463^*^ | 3121.915 | 0.000 | 7120.476 | 28324.450 |
|  | ICA-only | 17117.065^*^ | 3167.468 | 0.000 | 6360.381 | 27873.749 |
|  | SIMPLE + ICA | 16606.110^*^ | 3088.191 | 0.000 | 6118.649 | 27093.570 |
|  | Phase-Randomised | 9399.910^*^ | 1691.023 | 0.000 | 3657.215 | 15142.605 |
| ICA + PNM | SIMPLE | -20005.961^*^ | 3296.270 | 0.000 | -31200.055 | -8811.866 |
|  | PNM-only | -17969.207^*^ | 2910.450 | 0.000 | -27853.060 | -8085.353 |
|  | SIMPLE + PNM | -17722.463^*^ | 3121.915 | 0.000 | -28324.450 | -7120.476 |
|  | ICA-only | -605.398 | 352.256 | 1.000 | -1801.656 | 590.859 |
|  | SIMPLE + ICA | -1116.354^*^ | 313.657 | 0.033 | -2181.529 | -51.179 |
|  | Phase-Randomised | -8322.553^*^ | 1655.376 | 0.001 | -13944.191 | -2700.915 |
| ICA-only | SIMPLE | -19400.563^*^ | 3309.757 | 0.000 | -30640.459 | -8160.667 |
|  | PNM-only | -17363.808^*^ | 2946.583 | 0.000 | -27370.371 | -7357.246 |
|  | SIMPLE + PNM | -17117.065^*^ | 3167.468 | 0.000 | -27873.749 | -6360.381 |
|  | ICA + PNM | 605.398 | 352.256 | 1.000 | -590.859 | 1801.656 |
|  | SIMPLE + ICA | -510.955 | 410.132 | 1.000 | -1903.758 | 881.848 |
|  | Phase-Randomised | -7717.155^*^ | 1656.827 | 0.002 | -13343.721 | -2090.589 |
| SIMPLE + ICA | SIMPLE | -18889.607^*^ | 3257.892 | 0.000 | -29953.370 | -7825.844 |
|  | PNM-only | -16852.853^*^ | 2886.050 | 0.000 | -26653.844 | -7051.862 |
|  | SIMPLE + PNM | -16606.110^*^ | 3088.191 | 0.000 | -27093.570 | -6118.649 |
|  | ICA + PNM | 1116.354^*^ | 313.657 | 0.033 | 51.179 | 2181.529 |
|  | ICA-only | 510.955 | 410.132 | 1.000 | -881.848 | 1903.758 |
|  | Phase-Randomised | -7206.199^*^ | 1625.599 | 0.004 | -12726.716 | -1685.683 |
| 7 | SIMPLE | -11683.408^*^ | 1779.632 | 0.000 | -17727.018 | -5639.797 |
|  | PNM-only | -9646.654^*^ | 1577.924 | 0.000 | -15005.264 | -4288.043 |
|  | SIMPLE + PNM | -9399.910^*^ | 1691.023 | 0.000 | -15142.605 | -3657.215 |
|  | ICA + PNM | 8322.553^*^ | 1655.376 | 0.001 | 2700.915 | 13944.191 |
|  | ICA-only | 7717.155^*^ | 1656.827 | 0.002 | 2090.589 | 13343.721 |
|  | SIMPLE + ICA | 7206.199^*^ | 1625.599 | 0.004 | 1685.683 | 12726.716 |
| Based on estimated marginal means | | | | | | |
| *. The mean difference is significant at the .05 level. | | | | | | |
| b. Adjustment for multiple comparisons: Bonferroni. | | | | | | |

**Supplementary Table 3A.)** Post-hoc paired *t*-tests between drug modulations in connectivity between pre-processing pipelines, across RSNs, for connectivity changes within RSNs

| (I) Pipeline | | Mean Difference (I-J) | Std. Error | Sig.^b^ | 95% Confidence Interval for Difference^b^ | |
| --- | --- | --- | --- | --- | --- | --- |
|  |  |  |  |  | Lower Bound | Upper Bound |
| SIMPLE | PNM-only | 3920.521^*^ | 394.206 | 0.000 | 2581.801 | 5259.241 |
|  | SIMPLE + PNM | 791.183^*^ | 90.794 | 0.000 | 482.848 | 1099.518 |
|  | ICA + PNM | 2770.401^*^ | 310.529 | 0.000 | 1715.848 | 3824.953 |
|  | ICA-only | 2605.941^*^ | 309.623 | 0.000 | 1554.465 | 3657.417 |
|  | SIMPLE + ICA | 2664.032^*^ | 311.896 | 0.000 | 1604.838 | 3723.226 |
|  | Phase-Randomised | 1451.418^*^ | 190.459 | 0.000 | 804.621 | 2098.216 |
| PNM-only | SIMPLE | -3920.521^*^ | 394.206 | 0.000 | -5259.241 | -2581.801 |
|  | PNM-only | -3129.338^*^ | 360.604 | 0.000 | -4353.944 | -1904.733 |
|  | ICA + PNM | -1150.120^*^ | 256.997 | 0.003 | -2022.880 | -277.361 |
|  | ICA-only | -1314.580^*^ | 257.468 | 0.001 | -2188.938 | -440.222 |
|  | SIMPLE + ICA | -1256.489^*^ | 253.012 | 0.001 | -2115.717 | -397.262 |
|  | Phase-Randomised | -2469.103^*^ | 280.125 | 0.000 | -3420.403 | -1517.802 |
| SIMPLE + PNM | SIMPLE | -791.183^*^ | 90.794 | 0.000 | -1099.518 | -482.848 |
|  | PNM-only | 3129.338^*^ | 360.604 | 0.000 | 1904.733 | 4353.944 |
|  | ICA + PNM | 1979.218^*^ | 248.110 | 0.000 | 1136.640 | 2821.796 |
|  | ICA-only | 1814.758^*^ | 247.479 | 0.000 | 974.323 | 2655.194 |
|  | SIMPLE + ICA | 1872.849^*^ | 249.337 | 0.000 | 1026.103 | 2719.595 |
|  | Phase-Randomised | 660.236^*^ | 139.532 | 0.002 | 186.386 | 1134.086 |
| ICA + PNM | SIMPLE | -2770.401^*^ | 310.529 | 0.000 | -3824.953 | -1715.848 |
|  | PNM-only | 1150.120^*^ | 256.997 | 0.003 | 277.361 | 2022.880 |
|  | SIMPLE + PNM | -1979.218^*^ | 248.110 | 0.000 | -2821.796 | -1136.640 |
|  | ICA-only | -164.459^*^ | 20.423 | 0.000 | -233.816 | -95.103 |
|  | SIMPLE + ICA | -106.369^*^ | 18.994 | 0.000 | -170.871 | -41.867 |
|  | Phase-Randomised | -1318.982^*^ | 135.624 | 0.000 | -1779.558 | -858.406 |
| ICA-only | SIMPLE | -2605.941^*^ | 309.623 | 0.000 | -3657.417 | -1554.465 |
|  | PNM-only | 1314.580^*^ | 257.468 | 0.001 | 440.222 | 2188.938 |
|  | SIMPLE + PNM | -1814.758^*^ | 247.479 | 0.000 | -2655.194 | -974.323 |
|  | ICA + PNM | 164.459^*^ | 20.423 | 0.000 | 95.103 | 233.816 |
|  | SIMPLE + ICA | 58.091 | 17.340 | 0.056 | -0.797 | 116.979 |
|  | Phase-Randomised | -1154.523^*^ | 133.123 | 0.000 | -1606.606 | -702.439 |
| SIMPLE + ICA | SIMPLE | -2664.032^*^ | 311.896 | 0.000 | -3723.226 | -1604.838 |
|  | PNM-only | 1256.489^*^ | 253.012 | 0.001 | 397.262 | 2115.717 |
|  | SIMPLE + PNM | -1872.849^*^ | 249.337 | 0.000 | -2719.595 | -1026.103 |
|  | ICA + PNM | 106.369^*^ | 18.994 | 0.000 | 41.867 | 170.871 |
|  | ICA-only | -58.091 | 17.340 | 0.056 | -116.979 | 0.797 |
|  | Phase-Randomised | -1212.613^*^ | 136.367 | 0.000 | -1675.713 | -749.513 |
| 7 | SIMPLE | -1451.418^*^ | 190.459 | 0.000 | -2098.216 | -804.621 |
|  | PNM-only | 2469.103^*^ | 280.125 | 0.000 | 1517.802 | 3420.403 |
|  | SIMPLE + PNM | -660.236^*^ | 139.532 | 0.002 | -1134.086 | -186.386 |
|  | ICA + PNM | 1318.982^*^ | 135.624 | 0.000 | 858.406 | 1779.558 |
|  | ICA-only | 1154.523^*^ | 133.123 | 0.000 | 702.439 | 1606.606 |
|  | SIMPLE + ICA | 1212.613^*^ | 136.367 | 0.000 | 749.513 | 1675.713 |
| Based on estimated marginal means | | | | | | |
| *. The mean difference is significant at the .05 level. | | | | | | |
| b. Adjustment for multiple comparisons: Bonferroni. | | | | | | |

**Supplementary Table 3B.)** Post-hoc paired *t*-tests between drug modulations in connectivity between pre-processing pipelines, across RSNs, for connectivity changes between RSNs and the rest of the brain

| Pipeline | Mean | Std. Error | 95% Confidence Interval | |
| --- | --- | --- | --- | --- |
|  |  |  | Lower Bound | Upper Bound |
| SIMPLE | 26419.661 | 3305.209 | 19598.045 | 33241.276 |
| PNM-only | 24382.907 | 2980.628 | 18231.192 | 30534.621 |
| SIMPLE + PNM | 24136.163 | 3224.024 | 17482.105 | 30790.221 |
| ICA + PNM | 6413.700 | 1067.874 | 4209.717 | 8617.683 |
| ICA-only | 7019.098 | 1071.575 | 4807.476 | 9230.720 |
| SIMPLE + ICA | 7530.054 | 1107.679 | 5243.916 | 9816.191 |
| Phase-Randomised | 14736.253 | 1711.940 | 11202.983 | 18269.523 |

**Supplementary Table 3C.)** Average changes in connectivity within RSNS after drug administration, across RSNs, for each pipeline

| Pipeline | Mean | Std. Error | 95% Confidence Interval | |
| --- | --- | --- | --- | --- |
|  |  |  | Lower Bound | Upper Bound |
| SIMPLE | 2754.054 | 305.293 | 2123.960 | 3384.147 |
| PNM-only | -1166.467 | 252.565 | -1687.736 | -645.198 |
| SIMPLE + PNM | 1962.871 | 244.349 | 1458.559 | 2467.183 |
| ICA + PNM | -16.347 | 17.244 | -51.938 | 19.244 |
| ICA-only | 148.113 | 27.569 | 91.214 | 205.011 |
| SIMPLE + ICA | 90.022 | 27.753 | 32.743 | 147.301 |
| Phase-Randomised | 1302.635 | 128.054 | 1038.344 | 1566.927 |

**Supplementary Table 3D.)** Average changes in connectivity between RSNS and the rest of the brain after drug administration, across RSNs, for each pipeline

| RSN | | | Mean Difference (I-J) | Std. Error | Sig.^b^ | 95% Confidence Interval for Difference^b^ | |
| --- | --- | --- | --- | --- | --- | --- | --- |
|  |  |  |  |  |  | Lower Bound | Upper Bound |
| rFPN | SIMPLE | PNM-only | 2458.899^*^ | 300.518 | 0.000 | 1438.345 | 3479.453 |
|  |  | SIMPLE + PNM | 0.000 | 0.000 |  | 0.000 | 0.000 |
|  |  | ICA + PNM | 578.688^*^ | 63.440 | 0.000 | 363.248 | 794.129 |
|  |  | ICA-only | 714.986^*^ | 84.651 | 0.000 | 427.510 | 1002.461 |
|  |  | SIMPLE + ICA | 13.888^*^ | 2.046 | 0.000 | 6.941 | 20.835 |
|  |  | Phase-Randomised | 0.000 | 0.000 |  | 0.000 | 0.000 |
|  | PNM-only | SIMPLE | -2458.899^*^ | 300.518 | 0.000 | -3479.453 | -1438.345 |
|  |  | SIMPLE + PNM | -2458.899^*^ | 300.518 | 0.000 | -3479.453 | -1438.345 |
|  |  | ICA + PNM | -1880.211^*^ | 298.841 | 0.000 | -2895.072 | -865.350 |
|  |  | ICA-only | -1743.913^*^ | 286.044 | 0.000 | -2715.314 | -772.513 |
|  |  | SIMPLE + ICA | -2445.011^*^ | 299.623 | 0.000 | -3462.529 | -1427.493 |
|  |  | Phase-Randomised | -2458.899^*^ | 300.518 | 0.000 | -3479.453 | -1438.345 |
|  | SIMPLE + PNM | SIMPLE | 0.000 | 0.000 |  | 0.000 | 0.000 |
|  |  | PNM-only | 2458.899^*^ | 300.518 | 0.000 | 1438.345 | 3479.453 |
|  |  | ICA + PNM | 578.688^*^ | 63.440 | 0.000 | 363.248 | 794.129 |
|  |  | ICA-only | 714.986^*^ | 84.651 | 0.000 | 427.510 | 1002.461 |
|  |  | SIMPLE + ICA | 13.888^*^ | 2.046 | 0.000 | 6.941 | 20.835 |
|  |  | Phase-Randomised | 0.000 | 0.000 |  | 0.000 | 0.000 |
|  | ICA + PNM | SIMPLE | -578.688^*^ | 63.440 | 0.000 | -794.129 | -363.248 |
|  |  | PNM-only | 1880.211^*^ | 298.841 | 0.000 | 865.350 | 2895.072 |
|  |  | SIMPLE + PNM | -578.688^*^ | 63.440 | 0.000 | -794.129 | -363.248 |
|  |  | ICA-only | 136.297 | 58.175 | 0.583 | -61.264 | 333.859 |
|  |  | SIMPLE + ICA | -564.800^*^ | 62.750 | 0.000 | -777.898 | -351.702 |
|  |  | Phase-Randomised | -578.688^*^ | 63.440 | 0.000 | -794.129 | -363.248 |
|  | ICA-only | SIMPLE | -714.986^*^ | 84.651 | 0.000 | -1002.461 | -427.510 |
|  |  | PNM-only | 1743.913^*^ | 286.044 | 0.000 | 772.513 | 2715.314 |
|  |  | SIMPLE + PNM | -714.986^*^ | 84.651 | 0.000 | -1002.461 | -427.510 |
|  |  | ICA + PNM | -136.297 | 58.175 | 0.583 | -333.859 | 61.264 |
|  |  | SIMPLE + ICA | -701.098^*^ | 83.585 | 0.000 | -984.951 | -417.244 |
|  |  | Phase-Randomised | -714.986^*^ | 84.651 | 0.000 | -1002.461 | -427.510 |
|  | SIMPLE + ICA | SIMPLE | -13.888^*^ | 2.046 | 0.000 | -20.835 | -6.941 |
|  |  | PNM-only | 2445.011^*^ | 299.623 | 0.000 | 1427.493 | 3462.529 |
|  |  | SIMPLE + PNM | -13.888^*^ | 2.046 | 0.000 | -20.835 | -6.941 |
|  |  | ICA + PNM | 564.800^*^ | 62.750 | 0.000 | 351.702 | 777.898 |
|  |  | ICA-only | 701.098^*^ | 83.585 | 0.000 | 417.244 | 984.951 |
|  |  | Phase-Randomised | -13.888^*^ | 2.046 | 0.000 | -20.835 | -6.941 |
|  | Phase-Randomised | SIMPLE | 0.000 | 0.000 |  | 0.000 | 0.000 |
|  |  | PNM-only | 2458.899^*^ | 300.518 | 0.000 | 1438.345 | 3479.453 |
|  |  | SIMPLE + PNM | 0.000 | 0.000 |  | 0.000 | 0.000 |
|  |  | ICA + PNM | 578.688^*^ | 63.440 | 0.000 | 363.248 | 794.129 |
|  |  | ICA-only | 714.986^*^ | 84.651 | 0.000 | 427.510 | 1002.461 |
|  |  | SIMPLE + ICA | 13.888^*^ | 2.046 | 0.000 | 6.941 | 20.835 |
| lFPN | SIMPLE | PNM-only | 11.458^*^ | 2.485 | 0.002 | 3.018 | 19.897 |
|  |  | SIMPLE + PNM | 3.341 | 1.482 | 0.704 | -1.690 | 8.372 |
|  |  | ICA + PNM | 23.431^*^ | 2.909 | 0.000 | 13.552 | 33.310 |
|  |  | ICA-only | 148.765^*^ | 19.360 | 0.000 | 83.019 | 214.511 |
|  |  | SIMPLE + ICA | 11.458^*^ | 2.485 | 0.002 | 3.018 | 19.897 |
|  |  | Phase-Randomised | 11.458^*^ | 2.485 | 0.002 | 3.018 | 19.897 |
|  | PNM-only | SIMPLE | -11.458^*^ | 2.485 | 0.002 | -19.897 | -3.018 |
|  |  | SIMPLE + PNM | -8.117^*^ | 1.757 | 0.002 | -14.084 | -2.149 |
|  |  | ICA + PNM | 11.974^*^ | 1.264 | 0.000 | 7.682 | 16.265 |
|  |  | ICA-only | 137.308^*^ | 19.039 | 0.000 | 72.653 | 201.962 |
|  |  | SIMPLE + ICA | 0.000 | 0.000 |  | 0.000 | 0.000 |
|  |  | Phase-Randomised | 0.000 | 0.000 |  | 0.000 | 0.000 |
|  | SIMPLE + PNM | SIMPLE | -3.341 | 1.482 | 0.704 | -8.372 | 1.690 |
|  |  | PNM-only | 8.117^*^ | 1.757 | 0.002 | 2.149 | 14.084 |
|  |  | ICA + PNM | 20.090^*^ | 2.222 | 0.000 | 12.545 | 27.635 |
|  |  | ICA-only | 145.424^*^ | 19.104 | 0.000 | 80.549 | 210.300 |
|  |  | SIMPLE + ICA | 8.117^*^ | 1.757 | 0.002 | 2.149 | 14.084 |
|  |  | Phase-Randomised | 8.117^*^ | 1.757 | 0.002 | 2.149 | 14.084 |
|  | ICA + PNM | SIMPLE | -23.431^*^ | 2.909 | 0.000 | -33.310 | -13.552 |
|  |  | PNM-only | -11.974^*^ | 1.264 | 0.000 | -16.265 | -7.682 |
|  |  | SIMPLE + PNM | -20.090^*^ | 2.222 | 0.000 | -27.635 | -12.545 |
|  |  | ICA-only | 125.334^*^ | 18.744 | 0.000 | 61.678 | 188.990 |
|  |  | SIMPLE + ICA | -11.974^*^ | 1.264 | 0.000 | -16.265 | -7.682 |
|  |  | Phase-Randomised | -11.974^*^ | 1.264 | 0.000 | -16.265 | -7.682 |
|  | ICA-only | SIMPLE | -148.765^*^ | 19.360 | 0.000 | -214.511 | -83.019 |
|  |  | PNM-only | -137.308^*^ | 19.039 | 0.000 | -201.962 | -72.653 |
|  |  | SIMPLE + PNM | -145.424^*^ | 19.104 | 0.000 | -210.300 | -80.549 |
|  |  | ICA + PNM | -125.334^*^ | 18.744 | 0.000 | -188.990 | -61.678 |
|  |  | SIMPLE + ICA | -137.308^*^ | 19.039 | 0.000 | -201.962 | -72.653 |
|  |  | Phase-Randomised | -137.308^*^ | 19.039 | 0.000 | -201.962 | -72.653 |
|  | SIMPLE + ICA | SIMPLE | -11.458^*^ | 2.485 | 0.002 | -19.897 | -3.018 |
|  |  | PNM-only | 0.000 | 0.000 |  | 0.000 | 0.000 |
|  |  | SIMPLE + PNM | -8.117^*^ | 1.757 | 0.002 | -14.084 | -2.149 |
|  |  | ICA + PNM | 11.974^*^ | 1.264 | 0.000 | 7.682 | 16.265 |
|  |  | ICA-only | 137.308^*^ | 19.039 | 0.000 | 72.653 | 201.962 |
|  |  | Phase-Randomised | 0.000 | 0.000 |  | 0.000 | 0.000 |
|  | Phase-Randomised | SIMPLE | -11.458^*^ | 2.485 | 0.002 | -19.897 | -3.018 |
|  |  | PNM-only | 0.000 | 0.000 |  | 0.000 | 0.000 |
|  |  | SIMPLE + PNM | -8.117^*^ | 1.757 | 0.002 | -14.084 | -2.149 |
|  |  | ICA + PNM | 11.974^*^ | 1.264 | 0.000 | 7.682 | 16.265 |
|  |  | ICA-only | 137.308^*^ | 19.039 | 0.000 | 72.653 | 201.962 |
|  |  | SIMPLE + ICA | 0.000 | 0.000 |  | 0.000 | 0.000 |
| SMN | SIMPLE | PNM-only | 10540.328^*^ | 3017.631 | 0.039 | 292.488 | 20788.167 |
|  |  | SIMPLE + PNM | 14030.711^*^ | 2837.370 | 0.001 | 4395.037 | 23666.384 |
|  |  | ICA + PNM | 88936.466^*^ | 15280.480 | 0.000 | 37044.136 | 140828.796 |
|  |  | ICA-only | 89663.800^*^ | 15292.915 | 0.000 | 37729.240 | 141598.359 |
|  |  | SIMPLE + ICA | 89119.176^*^ | 15277.944 | 0.000 | 37235.458 | 141002.894 |
|  |  | Phase-Randomised | 40428.904^*^ | 8305.196 | 0.001 | 12224.557 | 68633.251 |
|  | PNM-only | SIMPLE | -10540.328^*^ | 3017.631 | 0.039 | -20788.167 | -292.488 |
|  |  | SIMPLE + PNM | 3490.383 | 2028.167 | 1.000 | -3397.247 | 10378.013 |
|  |  | ICA + PNM | 78396.138^*^ | 14566.239 | 0.000 | 28929.361 | 127862.914 |
|  |  | ICA-only | 79123.472^*^ | 14591.616 | 0.000 | 29570.514 | 128676.429 |
|  |  | SIMPLE + ICA | 78578.848^*^ | 14569.392 | 0.000 | 29101.364 | 128056.333 |
|  |  | Phase-Randomised | 29888.576^*^ | 7672.019 | 0.014 | 3834.491 | 55942.661 |
|  | SIMPLE + PNM | SIMPLE | -14030.711^*^ | 2837.370 | 0.001 | -23666.384 | -4395.037 |
|  |  | PNM-only | -3490.383 | 2028.167 | 1.000 | -10378.013 | 3397.247 |
|  |  | ICA + PNM | 74905.755^*^ | 13878.378 | 0.000 | 27774.944 | 122036.565 |
|  |  | ICA-only | 75633.089^*^ | 13901.194 | 0.000 | 28424.796 | 122841.382 |
|  |  | SIMPLE + ICA | 75088.466^*^ | 13881.317 | 0.000 | 27947.677 | 122229.255 |
|  |  | Phase-Randomised | 26398.193^*^ | 6839.301 | 0.016 | 3172.008 | 49624.379 |
|  | ICA + PNM | SIMPLE | -88936.466^*^ | 15280.480 | 0.000 | -140828.796 | -37044.136 |
|  |  | PNM-only | -78396.138^*^ | 14566.239 | 0.000 | -127862.914 | -28929.361 |
|  |  | SIMPLE + PNM | -74905.755^*^ | 13878.378 | 0.000 | -122036.565 | -27774.944 |
|  |  | ICA-only | 727.334^*^ | 147.363 | 0.001 | 226.892 | 1227.775 |
|  |  | SIMPLE + ICA | 182.711 | 63.835 | 0.180 | -34.073 | 399.494 |
|  |  | Phase-Randomised | -48507.562^*^ | 7523.137 | 0.000 | -74056.048 | -22959.075 |
|  | ICA-only | SIMPLE | -89663.800^*^ | 15292.915 | 0.000 | -141598.359 | -37729.240 |
|  |  | PNM-only | -79123.472^*^ | 14591.616 | 0.000 | -128676.429 | -29570.514 |
|  |  | SIMPLE + PNM | -75633.089^*^ | 13901.194 | 0.000 | -122841.382 | -28424.796 |
|  |  | ICA + PNM | -727.334^*^ | 147.363 | 0.001 | -1227.775 | -226.892 |
|  |  | SIMPLE + ICA | -544.623^*^ | 103.062 | 0.000 | -894.622 | -194.624 |
|  |  | Phase-Randomised | -49234.896^*^ | 7549.663 | 0.000 | -74873.463 | -23596.328 |
|  | SIMPLE + ICA | SIMPLE | -89119.176^*^ | 15277.944 | 0.000 | -141002.894 | -37235.458 |
|  |  | PNM-only | -78578.848^*^ | 14569.392 | 0.000 | -128056.333 | -29101.364 |
|  |  | SIMPLE + PNM | -75088.466^*^ | 13881.317 | 0.000 | -122229.255 | -27947.677 |
|  |  | ICA + PNM | -182.711 | 63.835 | 0.180 | -399.494 | 34.073 |
|  |  | ICA-only | 544.623^*^ | 103.062 | 0.000 | 194.624 | 894.622 |
|  |  | Phase-Randomised | -48690.272^*^ | 7526.065 | 0.000 | -74248.702 | -23131.843 |
|  | Phase-Randomised | SIMPLE | -40428.904^*^ | 8305.196 | 0.001 | -68633.251 | -12224.557 |
|  |  | PNM-only | -29888.576^*^ | 7672.019 | 0.014 | -55942.661 | -3834.491 |
|  |  | SIMPLE + PNM | -26398.193^*^ | 6839.301 | 0.016 | -49624.379 | -3172.008 |
|  |  | ICA + PNM | 48507.562^*^ | 7523.137 | 0.000 | 22959.075 | 74056.048 |
|  |  | ICA-only | 49234.896^*^ | 7549.663 | 0.000 | 23596.328 | 74873.463 |
|  |  | SIMPLE + ICA | 48690.272^*^ | 7526.065 | 0.000 | 23131.843 | 74248.702 |
| VN | SIMPLE | PNM-only | -801.617 | 3761.875 | 1.000 | -13576.902 | 11973.668 |
|  |  | SIMPLE + PNM | -336.408 | 1689.547 | 1.000 | -6074.088 | 5401.273 |
|  |  | ICA + PNM | 30473.749^*^ | 5632.153 | 0.000 | 11347.022 | 49600.476 |
|  |  | ICA-only | 25727.060^*^ | 5884.678 | 0.004 | 5742.763 | 45711.357 |
|  |  | SIMPLE + ICA | 24181.664^*^ | 5596.428 | 0.005 | 5176.260 | 43187.068 |
|  |  | Phase-Randomised | 29648.628^*^ | 3671.411 | 0.000 | 17180.560 | 42116.695 |
|  | PNM-only | SIMPLE | 801.617 | 3761.875 | 1.000 | -11973.668 | 13576.902 |
|  |  | SIMPLE + PNM | 465.209 | 3838.285 | 1.000 | -12569.562 | 13499.981 |
|  |  | ICA + PNM | 31275.366^*^ | 5349.259 | 0.000 | 13109.346 | 49441.385 |
|  |  | ICA-only | 26528.677^*^ | 5820.468 | 0.003 | 6762.436 | 46294.918 |
|  |  | SIMPLE + ICA | 24983.281^*^ | 5672.284 | 0.004 | 5720.270 | 44246.291 |
|  |  | Phase-Randomised | 30450.244^*^ | 4725.726 | 0.000 | 14401.734 | 46498.755 |
|  | SIMPLE + PNM | SIMPLE | 336.408 | 1689.547 | 1.000 | -5401.273 | 6074.088 |
|  |  | PNM-only | -465.209 | 3838.285 | 1.000 | -13499.981 | 12569.562 |
|  |  | ICA + PNM | 30810.156^*^ | 5985.717 | 0.001 | 10482.732 | 51137.581 |
|  |  | ICA-only | 26063.468^*^ | 6548.093 | 0.012 | 3826.222 | 48300.714 |
|  |  | SIMPLE + ICA | 24518.072^*^ | 5998.429 | 0.009 | 4147.477 | 44888.666 |
|  |  | Phase-Randomised | 29985.035^*^ | 4628.640 | 0.000 | 14266.228 | 45703.843 |
|  | ICA + PNM | SIMPLE | -30473.749^*^ | 5632.153 | 0.000 | -49600.476 | -11347.022 |
|  |  | PNM-only | -31275.366^*^ | 5349.259 | 0.000 | -49441.385 | -13109.346 |
|  |  | SIMPLE + PNM | -30810.156^*^ | 5985.717 | 0.001 | -51137.581 | -10482.732 |
|  |  | ICA-only | -4746.689 | 2079.566 | 0.664 | -11808.870 | 2315.493 |
|  |  | SIMPLE + ICA | -6292.085 | 1864.898 | 0.053 | -12625.256 | 41.086 |
|  |  | Phase-Randomised | -825.121 | 4250.541 | 1.000 | -15259.908 | 13609.666 |
|  | ICA-only | SIMPLE | -25727.060^*^ | 5884.678 | 0.004 | -45711.357 | -5742.763 |
|  |  | PNM-only | -26528.677^*^ | 5820.468 | 0.003 | -46294.918 | -6762.436 |
|  |  | SIMPLE + PNM | -26063.468^*^ | 6548.093 | 0.012 | -48300.714 | -3826.222 |
|  |  | ICA + PNM | 4746.689 | 2079.566 | 0.664 | -2315.493 | 11808.870 |
|  |  | SIMPLE + ICA | -1545.396 | 2400.619 | 1.000 | -9697.870 | 6607.078 |
|  |  | Phase-Randomised | 3921.568 | 4269.368 | 1.000 | -10577.155 | 18420.290 |
|  | SIMPLE + ICA | SIMPLE | -24181.664^*^ | 5596.428 | 0.005 | -43187.068 | -5176.260 |
|  |  | PNM-only | -24983.281^*^ | 5672.284 | 0.004 | -44246.291 | -5720.270 |
|  |  | SIMPLE + PNM | -24518.072^*^ | 5998.429 | 0.009 | -44888.666 | -4147.477 |
|  |  | ICA + PNM | 6292.085 | 1864.898 | 0.053 | -41.086 | 12625.256 |
|  |  | ICA-only | 1545.396 | 2400.619 | 1.000 | -6607.078 | 9697.870 |
|  |  | Phase-Randomised | 5466.964 | 4427.291 | 1.000 | -9568.066 | 20501.993 |
|  | Phase-Randomised | SIMPLE | -29648.628^*^ | 3671.411 | 0.000 | -42116.695 | -17180.560 |
|  |  | PNM-only | -30450.244^*^ | 4725.726 | 0.000 | -46498.755 | -14401.734 |
|  |  | SIMPLE + PNM | -29985.035^*^ | 4628.640 | 0.000 | -45703.843 | -14266.228 |
|  |  | ICA + PNM | 825.121 | 4250.541 | 1.000 | -13609.666 | 15259.908 |
|  |  | ICA-only | -3921.568 | 4269.368 | 1.000 | -18420.290 | 10577.155 |
|  |  | SIMPLE + ICA | -5466.964 | 4427.291 | 1.000 | -20501.993 | 9568.066 |
| pDMN | SIMPLE | PNM-only | 11.458^*^ | 2.485 | 0.002 | 3.018 | 19.897 |
|  |  | SIMPLE + PNM | 3.341 | 1.482 | 0.704 | -1.690 | 8.372 |
|  |  | ICA + PNM | 23.431^*^ | 2.909 | 0.000 | 13.552 | 33.310 |
|  |  | ICA-only | 148.765^*^ | 19.360 | 0.000 | 83.019 | 214.511 |
|  |  | SIMPLE + ICA | 11.458^*^ | 2.485 | 0.002 | 3.018 | 19.897 |
|  |  | Phase-Randomised | 11.458^*^ | 2.485 | 0.002 | 3.018 | 19.897 |
|  | PNM-only | SIMPLE | -11.458^*^ | 2.485 | 0.002 | -19.897 | -3.018 |
|  |  | SIMPLE + PNM | -8.117^*^ | 1.757 | 0.002 | -14.084 | -2.149 |
|  |  | ICA + PNM | 11.974^*^ | 1.264 | 0.000 | 7.682 | 16.265 |
|  |  | ICA-only | 137.308^*^ | 19.039 | 0.000 | 72.653 | 201.962 |
|  |  | SIMPLE + ICA | 0.000 | 0.000 |  | 0.000 | 0.000 |
|  |  | Phase-Randomised | 0.000 | 0.000 |  | 0.000 | 0.000 |
|  | SIMPLE + PNM | SIMPLE | -3.341 | 1.482 | 0.704 | -8.372 | 1.690 |
|  |  | PNM-only | 8.117^*^ | 1.757 | 0.002 | 2.149 | 14.084 |
|  |  | ICA + PNM | 20.090^*^ | 2.222 | 0.000 | 12.545 | 27.635 |
|  |  | ICA-only | 145.424^*^ | 19.104 | 0.000 | 80.549 | 210.300 |
|  |  | SIMPLE + ICA | 8.117^*^ | 1.757 | 0.002 | 2.149 | 14.084 |
|  |  | Phase-Randomised | 8.117^*^ | 1.757 | 0.002 | 2.149 | 14.084 |
|  | ICA + PNM | SIMPLE | -23.431^*^ | 2.909 | 0.000 | -33.310 | -13.552 |
|  |  | PNM-only | -11.974^*^ | 1.264 | 0.000 | -16.265 | -7.682 |
|  |  | SIMPLE + PNM | -20.090^*^ | 2.222 | 0.000 | -27.635 | -12.545 |
|  |  | ICA-only | 125.334^*^ | 18.744 | 0.000 | 61.678 | 188.990 |
|  |  | SIMPLE + ICA | -11.974^*^ | 1.264 | 0.000 | -16.265 | -7.682 |
|  |  | Phase-Randomised | -11.974^*^ | 1.264 | 0.000 | -16.265 | -7.682 |
|  | ICA-only | SIMPLE | -148.765^*^ | 19.360 | 0.000 | -214.511 | -83.019 |
|  |  | PNM-only | -137.308^*^ | 19.039 | 0.000 | -201.962 | -72.653 |
|  |  | SIMPLE + PNM | -145.424^*^ | 19.104 | 0.000 | -210.300 | -80.549 |
|  |  | ICA + PNM | -125.334^*^ | 18.744 | 0.000 | -188.990 | -61.678 |
|  |  | SIMPLE + ICA | -137.308^*^ | 19.039 | 0.000 | -201.962 | -72.653 |
|  |  | Phase-Randomised | -137.308^*^ | 19.039 | 0.000 | -201.962 | -72.653 |
|  | SIMPLE + ICA | SIMPLE | -11.458^*^ | 2.485 | 0.002 | -19.897 | -3.018 |
|  |  | PNM-only | 0.000 | 0.000 |  | 0.000 | 0.000 |
|  |  | SIMPLE + PNM | -8.117^*^ | 1.757 | 0.002 | -14.084 | -2.149 |
|  |  | ICA + PNM | 11.974^*^ | 1.264 | 0.000 | 7.682 | 16.265 |
|  |  | ICA-only | 137.308^*^ | 19.039 | 0.000 | 72.653 | 201.962 |
|  |  | Phase-Randomised | 0.000 | 0.000 |  | 0.000 | 0.000 |
|  | Phase-Randomised | SIMPLE | -11.458^*^ | 2.485 | 0.002 | -19.897 | -3.018 |
|  |  | PNM-only | 0.000 | 0.000 |  | 0.000 | 0.000 |
|  |  | SIMPLE + PNM | -8.117^*^ | 1.757 | 0.002 | -14.084 | -2.149 |
|  |  | ICA + PNM | 11.974^*^ | 1.264 | 0.000 | 7.682 | 16.265 |
|  |  | ICA-only | 137.308^*^ | 19.039 | 0.000 | 72.653 | 201.962 |
|  |  | SIMPLE + ICA | 0.000 | 0.000 |  | 0.000 | 0.000 |
| aDMN | SIMPLE | PNM-only | 0.000 | 0.000 |  | 0.000 | 0.000 |
|  |  | SIMPLE + PNM | 0.000 | 0.000 |  | 0.000 | 0.000 |
|  |  | ICA + PNM | 0.000 | 0.000 |  | 0.000 | 0.000 |
|  |  | ICA-only | 0.000 | 0.000 |  | 0.000 | 0.000 |
|  |  | SIMPLE + ICA | 0.000 | 0.000 |  | 0.000 | 0.000 |
|  |  | Phase-Randomised | 0.000 | 0.000 |  | 0.000 | 0.000 |
|  | PNM-only | SIMPLE | 0.000 | 0.000 |  | 0.000 | 0.000 |
|  |  | SIMPLE + PNM | 0.000 | 0.000 |  | 0.000 | 0.000 |
|  |  | ICA + PNM | 0.000 | 0.000 |  | 0.000 | 0.000 |
|  |  | ICA-only | 0.000 | 0.000 |  | 0.000 | 0.000 |
|  |  | SIMPLE + ICA | 0.000 | 0.000 |  | 0.000 | 0.000 |
|  |  | Phase-Randomised | 0.000 | 0.000 |  | 0.000 | 0.000 |
|  | SIMPLE + PNM | SIMPLE | 0.000 | 0.000 |  | 0.000 | 0.000 |
|  |  | PNM-only | 0.000 | 0.000 |  | 0.000 | 0.000 |
|  |  | ICA + PNM | 0.000 | 0.000 |  | 0.000 | 0.000 |
|  |  | ICA-only | 0.000 | 0.000 |  | 0.000 | 0.000 |
|  |  | SIMPLE + ICA | 0.000 | 0.000 |  | 0.000 | 0.000 |
|  |  | Phase-Randomised | 0.000 | 0.000 |  | 0.000 | 0.000 |
|  | ICA + PNM | SIMPLE | 0.000 | 0.000 |  | 0.000 | 0.000 |
|  |  | PNM-only | 0.000 | 0.000 |  | 0.000 | 0.000 |
|  |  | SIMPLE + PNM | 0.000 | 0.000 |  | 0.000 | 0.000 |
|  |  | ICA-only | 0.000 | 0.000 |  | 0.000 | 0.000 |
|  |  | SIMPLE + ICA | 0.000 | 0.000 |  | 0.000 | 0.000 |
|  |  | Phase-Randomised | 0.000 | 0.000 |  | 0.000 | 0.000 |
|  | ICA-only | SIMPLE | 0.000 | 0.000 |  | 0.000 | 0.000 |
|  |  | PNM-only | 0.000 | 0.000 |  | 0.000 | 0.000 |
|  |  | SIMPLE + PNM | 0.000 | 0.000 |  | 0.000 | 0.000 |
|  |  | ICA + PNM | 0.000 | 0.000 |  | 0.000 | 0.000 |
|  |  | SIMPLE + ICA | 0.000 | 0.000 |  | 0.000 | 0.000 |
|  |  | Phase-Randomised | 0.000 | 0.000 |  | 0.000 | 0.000 |
|  | SIMPLE + ICA | SIMPLE | 0.000 | 0.000 |  | 0.000 | 0.000 |
|  |  | PNM-only | 0.000 | 0.000 |  | 0.000 | 0.000 |
|  |  | SIMPLE + PNM | 0.000 | 0.000 |  | 0.000 | 0.000 |
|  |  | ICA + PNM | 0.000 | 0.000 |  | 0.000 | 0.000 |
|  |  | ICA-only | 0.000 | 0.000 |  | 0.000 | 0.000 |
|  |  | Phase-Randomised | 0.000 | 0.000 |  | 0.000 | 0.000 |
|  | Phase-Randomised | SIMPLE | 0.000 | 0.000 |  | 0.000 | 0.000 |
|  |  | PNM-only | 0.000 | 0.000 |  | 0.000 | 0.000 |
|  |  | SIMPLE + PNM | 0.000 | 0.000 |  | 0.000 | 0.000 |
|  |  | ICA + PNM | 0.000 | 0.000 |  | 0.000 | 0.000 |
|  |  | ICA-only | 0.000 | 0.000 |  | 0.000 | 0.000 |
|  |  | SIMPLE + ICA | 0.000 | 0.000 |  | 0.000 | 0.000 |
| Based on estimated marginal means | | | | | | | |
| *. The mean difference is significant at the .05 level. | | | | | | | |
| b. Adjustment for multiple comparisons: Bonferroni. | | | | | | | |

**Supplementary Table 3E.)** Post-hoc paired *t*-tests between all pipelines for each RSN separately, for connectivity changes after drug administration within RSNs

| RSN | | | Mean Difference (I-J) | Std. Error | Sig.^b^ | 95% Confidence Interval for Difference^b^ | |
| --- | --- | --- | --- | --- | --- | --- | --- |
|  |  |  |  |  |  | Lower Bound | Upper Bound |
| rFPN | SIMPLE | PNM-only | 17284.811^*^ | 1923.542 | 0.000 | 10752.486 | 23817.135 |
|  |  | SIMPLE + PNM | -120.892^*^ | 19.859 | 0.000 | -188.334 | -53.451 |
|  |  | ICA + PNM | -145.599^*^ | 21.197 | 0.000 | -217.584 | -73.614 |
|  |  | ICA-only | -112.437^*^ | 21.989 | 0.001 | -187.110 | -37.763 |
|  |  | SIMPLE + ICA | -148.730^*^ | 21.262 | 0.000 | -220.936 | -76.523 |
|  |  | Phase-Randomised | -225.398^*^ | 22.310 | 0.000 | -301.163 | -149.632 |
|  | PNM-only | SIMPLE | -17284.811^*^ | 1923.542 | 0.000 | -23817.135 | -10752.486 |
|  |  | SIMPLE + PNM | -17405.703^*^ | 1926.638 | 0.000 | -23948.543 | -10862.862 |
|  |  | ICA + PNM | -17430.410^*^ | 1927.198 | 0.000 | -23975.152 | -10885.668 |
|  |  | ICA-only | -17397.247^*^ | 1926.781 | 0.000 | -23940.572 | -10853.922 |
|  |  | SIMPLE + ICA | -17433.540^*^ | 1927.327 | 0.000 | -23978.722 | -10888.359 |
|  |  | Phase-Randomised | -17510.208^*^ | 1931.988 | 0.000 | -24071.217 | -10949.200 |
|  | SIMPLE + PNM | SIMPLE | 120.892^*^ | 19.859 | 0.000 | 53.451 | 188.334 |
|  |  | PNM-only | 17405.703^*^ | 1926.638 | 0.000 | 10862.862 | 23948.543 |
|  |  | ICA + PNM | -24.707^*^ | 4.695 | 0.000 | -40.653 | -8.761 |
|  |  | ICA-only | 8.456 | 6.606 | 1.000 | -13.977 | 30.888 |
|  |  | SIMPLE + ICA | -27.838^*^ | 4.569 | 0.000 | -43.355 | -12.320 |
|  |  | Phase-Randomised | -104.506^*^ | 11.694 | 0.000 | -144.218 | -64.793 |
|  | ICA + PNM | SIMPLE | 145.599^*^ | 21.197 | 0.000 | 73.614 | 217.584 |
|  |  | PNM-only | 17430.410^*^ | 1927.198 | 0.000 | 10885.668 | 23975.152 |
|  |  | SIMPLE + PNM | 24.707^*^ | 4.695 | 0.000 | 8.761 | 40.653 |
|  |  | ICA-only | 33.163^*^ | 4.246 | 0.000 | 18.743 | 47.582 |
|  |  | SIMPLE + ICA | -3.131^*^ | 0.726 | 0.005 | -5.597 | -0.664 |
|  |  | Phase-Randomised | -79.799^*^ | 9.939 | 0.000 | -113.552 | -46.045 |
|  | ICA-only | SIMPLE | 112.437^*^ | 21.989 | 0.001 | 37.763 | 187.110 |
|  |  | PNM-only | 17397.247^*^ | 1926.781 | 0.000 | 10853.922 | 23940.572 |
|  |  | SIMPLE + PNM | -8.456 | 6.606 | 1.000 | -30.888 | 13.977 |
|  |  | ICA + PNM | -33.163^*^ | 4.246 | 0.000 | -47.582 | -18.743 |
|  |  | SIMPLE + ICA | -36.293^*^ | 4.214 | 0.000 | -50.603 | -21.983 |
|  |  | Phase-Randomised | -112.961^*^ | 10.244 | 0.000 | -147.749 | -78.174 |
|  | SIMPLE + ICA | SIMPLE | 148.730^*^ | 21.262 | 0.000 | 76.523 | 220.936 |
|  |  | PNM-only | 17433.540^*^ | 1927.327 | 0.000 | 10888.359 | 23978.722 |
|  |  | SIMPLE + PNM | 27.838^*^ | 4.569 | 0.000 | 12.320 | 43.355 |
|  |  | ICA + PNM | 3.131^*^ | 0.726 | 0.005 | 0.664 | 5.597 |
|  |  | ICA-only | 36.293^*^ | 4.214 | 0.000 | 21.983 | 50.603 |
|  |  | Phase-Randomised | -76.668^*^ | 9.806 | 0.000 | -109.970 | -43.366 |
|  | Phase-Randomised | SIMPLE | 225.398^*^ | 22.310 | 0.000 | 149.632 | 301.163 |
|  |  | PNM-only | 17510.208^*^ | 1931.988 | 0.000 | 10949.200 | 24071.217 |
|  |  | SIMPLE + PNM | 104.506^*^ | 11.694 | 0.000 | 64.793 | 144.218 |
|  |  | ICA + PNM | 79.799^*^ | 9.939 | 0.000 | 46.045 | 113.552 |
|  |  | ICA-only | 112.961^*^ | 10.244 | 0.000 | 78.174 | 147.749 |
|  |  | SIMPLE + ICA | 76.668^*^ | 9.806 | 0.000 | 43.366 | 109.970 |
| lFPN | SIMPLE | PNM-only | 338.684^*^ | 45.663 | 0.000 | 183.612 | 493.756 |
|  |  | SIMPLE + PNM | 230.348^*^ | 34.116 | 0.000 | 114.490 | 346.207 |
|  |  | ICA + PNM | 344.617^*^ | 45.466 | 0.000 | 190.215 | 499.019 |
|  |  | ICA-only | 366.261^*^ | 46.956 | 0.000 | 206.798 | 525.724 |
|  |  | SIMPLE + ICA | 338.684^*^ | 45.663 | 0.000 | 183.612 | 493.756 |
|  |  | Phase-Randomised | 184.899^*^ | 39.012 | 0.002 | 52.415 | 317.383 |
|  | PNM-only | SIMPLE | -338.684^*^ | 45.663 | 0.000 | -493.756 | -183.612 |
|  |  | SIMPLE + PNM | -108.336^*^ | 13.422 | 0.000 | -153.916 | -62.756 |
|  |  | ICA + PNM | 5.933^*^ | 0.709 | 0.000 | 3.527 | 8.340 |
|  |  | ICA-only | 27.577^*^ | 3.665 | 0.000 | 15.130 | 40.024 |
|  |  | SIMPLE + ICA | 0.000 | 0.000 |  | 0.000 | 0.000 |
|  |  | Phase-Randomised | -153.785^*^ | 21.893 | 0.000 | -228.133 | -79.437 |
|  | SIMPLE + PNM | SIMPLE | -230.348^*^ | 34.116 | 0.000 | -346.207 | -114.490 |
|  |  | PNM-only | 108.336^*^ | 13.422 | 0.000 | 62.756 | 153.916 |
|  |  | ICA + PNM | 114.269^*^ | 13.222 | 0.000 | 69.366 | 159.171 |
|  |  | ICA-only | 135.913^*^ | 15.196 | 0.000 | 84.307 | 187.518 |
|  |  | SIMPLE + ICA | 108.336^*^ | 13.422 | 0.000 | 62.756 | 153.916 |
|  |  | Phase-Randomised | -45.449 | 20.730 | 0.804 | -115.849 | 24.950 |
|  | ICA + PNM | SIMPLE | -344.617^*^ | 45.466 | 0.000 | -499.019 | -190.215 |
|  |  | PNM-only | -5.933^*^ | 0.709 | 0.000 | -8.340 | -3.527 |
|  |  | SIMPLE + PNM | -114.269^*^ | 13.222 | 0.000 | -159.171 | -69.366 |
|  |  | ICA-only | 21.644^*^ | 3.538 | 0.000 | 9.629 | 33.659 |
|  |  | SIMPLE + ICA | -5.933^*^ | 0.709 | 0.000 | -8.340 | -3.527 |
|  |  | Phase-Randomised | -159.718^*^ | 21.762 | 0.000 | -233.623 | -85.813 |
|  | ICA-only | SIMPLE | -366.261^*^ | 46.956 | 0.000 | -525.724 | -206.798 |
|  |  | PNM-only | -27.577^*^ | 3.665 | 0.000 | -40.024 | -15.130 |
|  |  | SIMPLE + PNM | -135.913^*^ | 15.196 | 0.000 | -187.518 | -84.307 |
|  |  | ICA + PNM | -21.644^*^ | 3.538 | 0.000 | -33.659 | -9.629 |
|  |  | SIMPLE + ICA | -27.577^*^ | 3.665 | 0.000 | -40.024 | -15.130 |
|  |  | Phase-Randomised | -181.362^*^ | 22.204 | 0.000 | -256.765 | -105.959 |
|  | SIMPLE + ICA | SIMPLE | -338.684^*^ | 45.663 | 0.000 | -493.756 | -183.612 |
|  |  | PNM-only | 0.000 | 0.000 |  | 0.000 | 0.000 |
|  |  | SIMPLE + PNM | -108.336^*^ | 13.422 | 0.000 | -153.916 | -62.756 |
|  |  | ICA + PNM | 5.933^*^ | 0.709 | 0.000 | 3.527 | 8.340 |
|  |  | ICA-only | 27.577^*^ | 3.665 | 0.000 | 15.130 | 40.024 |
|  |  | Phase-Randomised | -153.785^*^ | 21.893 | 0.000 | -228.133 | -79.437 |
|  | Phase-Randomised | SIMPLE | -184.899^*^ | 39.012 | 0.002 | -317.383 | -52.415 |
|  |  | PNM-only | 153.785^*^ | 21.893 | 0.000 | 79.437 | 228.133 |
|  |  | SIMPLE + PNM | 45.449 | 20.730 | 0.804 | -24.950 | 115.849 |
|  |  | ICA + PNM | 159.718^*^ | 21.762 | 0.000 | 85.813 | 233.623 |
|  |  | ICA-only | 181.362^*^ | 22.204 | 0.000 | 105.959 | 256.765 |
|  |  | SIMPLE + ICA | 153.785^*^ | 21.893 | 0.000 | 79.437 | 228.133 |
| SMN | SIMPLE | PNM-only | 5944.907^*^ | 679.454 | 0.000 | 3637.488 | 8252.325 |
|  |  | SIMPLE + PNM | 4704.393^*^ | 522.344 | 0.000 | 2930.519 | 6478.267 |
|  |  | ICA + PNM | 14743.153^*^ | 1715.745 | 0.000 | 8916.502 | 20569.805 |
|  |  | ICA-only | 14757.874^*^ | 1716.635 | 0.000 | 8928.202 | 20587.545 |
|  |  | SIMPLE + ICA | 14757.874^*^ | 1716.635 | 0.000 | 8928.202 | 20587.545 |
|  |  | Phase-Randomised | 7775.080^*^ | 1067.857 | 0.000 | 4148.650 | 11401.509 |
|  | PNM-only | SIMPLE | -5944.907^*^ | 679.454 | 0.000 | -8252.325 | -3637.488 |
|  |  | SIMPLE + PNM | -1240.514 | 414.178 | 0.132 | -2647.056 | 166.028 |
|  |  | ICA + PNM | 8798.247^*^ | 1200.504 | 0.000 | 4721.349 | 12875.145 |
|  |  | ICA-only | 8812.967^*^ | 1201.290 | 0.000 | 4733.402 | 12892.532 |
|  |  | SIMPLE + ICA | 8812.967^*^ | 1201.290 | 0.000 | 4733.402 | 12892.532 |
|  |  | Phase-Randomised | 1830.173 | 684.480 | 0.279 | -494.314 | 4154.661 |
|  | SIMPLE + PNM | SIMPLE | -4704.393^*^ | 522.344 | 0.000 | -6478.267 | -2930.519 |
|  |  | PNM-only | 1240.514 | 414.178 | 0.132 | -166.028 | 2647.056 |
|  |  | ICA + PNM | 10038.761^*^ | 1350.800 | 0.000 | 5451.458 | 14626.063 |
|  |  | ICA-only | 10053.481^*^ | 1351.773 | 0.000 | 5462.877 | 14644.085 |
|  |  | SIMPLE + ICA | 10053.481^*^ | 1351.773 | 0.000 | 5462.877 | 14644.085 |
|  |  | Phase-Randomised | 3070.687^*^ | 765.077 | 0.011 | 472.494 | 5668.880 |
|  | ICA + PNM | SIMPLE | -14743.153^*^ | 1715.745 | 0.000 | -20569.805 | -8916.502 |
|  |  | PNM-only | -8798.247^*^ | 1200.504 | 0.000 | -12875.145 | -4721.349 |
|  |  | SIMPLE + PNM | -10038.761^*^ | 1350.800 | 0.000 | -14626.063 | -5451.458 |
|  |  | ICA-only | 14.720^*^ | 2.913 | 0.001 | 4.828 | 24.613 |
|  |  | SIMPLE + ICA | 14.720^*^ | 2.913 | 0.001 | 4.828 | 24.613 |
|  |  | Phase-Randomised | -6968.074^*^ | 730.214 | 0.000 | -9447.871 | -4488.276 |
|  | ICA-only | SIMPLE | -14757.874^*^ | 1716.635 | 0.000 | -20587.545 | -8928.202 |
|  |  | PNM-only | -8812.967^*^ | 1201.290 | 0.000 | -12892.532 | -4733.402 |
|  |  | SIMPLE + PNM | -10053.481^*^ | 1351.773 | 0.000 | -14644.085 | -5462.877 |
|  |  | ICA + PNM | -14.720^*^ | 2.913 | 0.001 | -24.613 | -4.828 |
|  |  | SIMPLE + ICA | 0.000 | 0.000 |  | 0.000 | 0.000 |
|  |  | Phase-Randomised | -6982.794^*^ | 731.017 | 0.000 | -9465.318 | -4500.270 |
|  | SIMPLE + ICA | SIMPLE | -14757.874^*^ | 1716.635 | 0.000 | -20587.545 | -8928.202 |
|  |  | PNM-only | -8812.967^*^ | 1201.290 | 0.000 | -12892.532 | -4733.402 |
|  |  | SIMPLE + PNM | -10053.481^*^ | 1351.773 | 0.000 | -14644.085 | -5462.877 |
|  |  | ICA + PNM | -14.720^*^ | 2.913 | 0.001 | -24.613 | -4.828 |
|  |  | ICA-only | 0.000 | 0.000 |  | 0.000 | 0.000 |
|  |  | Phase-Randomised | -6982.794^*^ | 731.017 | 0.000 | -9465.318 | -4500.270 |
|  | Phase-Randomised | SIMPLE | -7775.080^*^ | 1067.857 | 0.000 | -11401.509 | -4148.650 |
|  |  | PNM-only | -1830.173 | 684.480 | 0.279 | -4154.661 | 494.314 |
|  |  | SIMPLE + PNM | -3070.687^*^ | 765.077 | 0.011 | -5668.880 | -472.494 |
|  |  | ICA + PNM | 6968.074^*^ | 730.214 | 0.000 | 4488.276 | 9447.871 |
|  |  | ICA-only | 6982.794^*^ | 731.017 | 0.000 | 4500.270 | 9465.318 |
|  |  | SIMPLE + ICA | 6982.794^*^ | 731.017 | 0.000 | 4500.270 | 9465.318 |
| VN | SIMPLE | PNM-only | -357.562^*^ | 95.565 | 0.021 | -682.100 | -33.024 |
|  |  | SIMPLE + PNM | -508.735^*^ | 93.199 | 0.000 | -825.236 | -192.234 |
|  |  | ICA + PNM | 608.755^*^ | 156.628 | 0.015 | 76.850 | 1140.660 |
|  |  | ICA-only | -305.168 | 204.275 | 1.000 | -998.885 | 388.548 |
|  |  | SIMPLE + ICA | -85.577 | 191.383 | 1.000 | -735.510 | 564.357 |
|  |  | Phase-Randomised | 574.508^*^ | 95.910 | 0.000 | 248.798 | 900.218 |
|  | PNM-only | SIMPLE | 357.562^*^ | 95.565 | 0.021 | 33.024 | 682.100 |
|  |  | SIMPLE + PNM | -151.174 | 120.350 | 1.000 | -559.881 | 257.534 |
|  |  | ICA + PNM | 966.317^*^ | 121.682 | 0.000 | 553.087 | 1379.547 |
|  |  | ICA-only | 52.394 | 150.999 | 1.000 | -460.396 | 565.183 |
|  |  | SIMPLE + ICA | 271.985 | 155.228 | 1.000 | -255.169 | 799.139 |
|  |  | Phase-Randomised | 932.070^*^ | 119.072 | 0.000 | 527.703 | 1336.436 |
|  | SIMPLE + PNM | SIMPLE | 508.735^*^ | 93.199 | 0.000 | 192.234 | 825.236 |
|  |  | PNM-only | 151.174 | 120.350 | 1.000 | -257.534 | 559.881 |
|  |  | ICA + PNM | 1117.490^*^ | 196.940 | 0.000 | 448.685 | 1786.296 |
|  |  | ICA-only | 203.567 | 220.385 | 1.000 | -544.858 | 951.993 |
|  |  | SIMPLE + ICA | 423.159 | 208.781 | 1.000 | -285.860 | 1132.178 |
|  |  | Phase-Randomised | 1083.243^*^ | 169.170 | 0.000 | 508.745 | 1657.741 |
|  | ICA + PNM | SIMPLE | -608.755^*^ | 156.628 | 0.015 | -1140.660 | -76.850 |
|  |  | PNM-only | -966.317^*^ | 121.682 | 0.000 | -1379.547 | -553.087 |
|  |  | SIMPLE + PNM | -1117.490^*^ | 196.940 | 0.000 | -1786.296 | -448.685 |
|  |  | ICA-only | -913.923^*^ | 114.634 | 0.000 | -1303.218 | -524.629 |
|  |  | SIMPLE + ICA | -694.332^*^ | 104.146 | 0.000 | -1048.009 | -340.654 |
|  |  | Phase-Randomised | -34.247 | 105.974 | 1.000 | -394.134 | 325.639 |
|  | ICA-only | SIMPLE | 305.168 | 204.275 | 1.000 | -388.548 | 998.885 |
|  |  | PNM-only | -52.394 | 150.999 | 1.000 | -565.183 | 460.396 |
|  |  | SIMPLE + PNM | -203.567 | 220.385 | 1.000 | -951.993 | 544.858 |
|  |  | ICA + PNM | 913.923^*^ | 114.634 | 0.000 | 524.629 | 1303.218 |
|  |  | SIMPLE + ICA | 219.591 | 86.488 | 0.378 | -74.122 | 513.305 |
|  |  | Phase-Randomised | 879.676^*^ | 175.079 | 0.001 | 285.110 | 1474.242 |
|  | SIMPLE + ICA | SIMPLE | 85.577 | 191.383 | 1.000 | -564.357 | 735.510 |
|  |  | PNM-only | -271.985 | 155.228 | 1.000 | -799.139 | 255.169 |
|  |  | SIMPLE + PNM | -423.159 | 208.781 | 1.000 | -1132.178 | 285.860 |
|  |  | ICA + PNM | 694.332^*^ | 104.146 | 0.000 | 340.654 | 1048.009 |
|  |  | ICA-only | -219.591 | 86.488 | 0.378 | -513.305 | 74.122 |
|  |  | Phase-Randomised | 660.085^*^ | 166.476 | 0.012 | 94.736 | 1225.434 |
|  | Phase-Randomised | SIMPLE | -574.508^*^ | 95.910 | 0.000 | -900.218 | -248.798 |
|  |  | PNM-only | -932.070^*^ | 119.072 | 0.000 | -1336.436 | -527.703 |
|  |  | SIMPLE + PNM | -1083.243^*^ | 169.170 | 0.000 | -1657.741 | -508.745 |
|  |  | ICA + PNM | 34.247 | 105.974 | 1.000 | -325.639 | 394.134 |
|  |  | ICA-only | -879.676^*^ | 175.079 | 0.001 | -1474.242 | -285.110 |
|  |  | SIMPLE + ICA | -660.085^*^ | 166.476 | 0.012 | -1225.434 | -94.736 |
| pDMN | SIMPLE | PNM-only | -8.745^*^ | 2.068 | 0.006 | -15.768 | -1.722 |
|  |  | SIMPLE + PNM | 60.092^*^ | 7.527 | 0.000 | 34.530 | 85.655 |
|  |  | ICA + PNM | 663.310^*^ | 56.744 | 0.000 | 470.608 | 856.011 |
|  |  | ICA-only | 520.950^*^ | 51.964 | 0.000 | 344.481 | 697.419 |
|  |  | SIMPLE + ICA | 713.773^*^ | 67.470 | 0.000 | 484.646 | 942.899 |
|  |  | Phase-Randomised | -8.745^*^ | 2.068 | 0.006 | -15.768 | -1.722 |
|  | PNM-only | SIMPLE | 8.745^*^ | 2.068 | 0.006 | 1.722 | 15.768 |
|  |  | SIMPLE + PNM | 68.838^*^ | 8.634 | 0.000 | 39.518 | 98.158 |
|  |  | ICA + PNM | 672.055^*^ | 57.139 | 0.000 | 478.012 | 866.097 |
|  |  | ICA-only | 529.695^*^ | 51.320 | 0.000 | 355.411 | 703.979 |
|  |  | SIMPLE + ICA | 722.518^*^ | 67.321 | 0.000 | 493.895 | 951.141 |
|  |  | Phase-Randomised | 0.000 | 0.000 |  | 0.000 | 0.000 |
|  | SIMPLE + PNM | SIMPLE | -60.092^*^ | 7.527 | 0.000 | -85.655 | -34.530 |
|  |  | PNM-only | -68.838^*^ | 8.634 | 0.000 | -98.158 | -39.518 |
|  |  | ICA + PNM | 603.217^*^ | 56.661 | 0.000 | 410.798 | 795.637 |
|  |  | ICA-only | 460.857^*^ | 54.622 | 0.000 | 275.361 | 646.354 |
|  |  | SIMPLE + ICA | 653.680^*^ | 68.768 | 0.000 | 420.146 | 887.214 |
|  |  | Phase-Randomised | -68.838^*^ | 8.634 | 0.000 | -98.158 | -39.518 |
|  | ICA + PNM | SIMPLE | -663.310^*^ | 56.744 | 0.000 | -856.011 | -470.608 |
|  |  | PNM-only | -672.055^*^ | 57.139 | 0.000 | -866.097 | -478.012 |
|  |  | SIMPLE + PNM | -603.217^*^ | 56.661 | 0.000 | -795.637 | -410.798 |
|  |  | ICA-only | -142.360 | 45.609 | 0.098 | -297.246 | 12.527 |
|  |  | SIMPLE + ICA | 50.463 | 52.095 | 1.000 | -126.450 | 227.376 |
|  |  | Phase-Randomised | -672.055^*^ | 57.139 | 0.000 | -866.097 | -478.012 |
|  | ICA-only | SIMPLE | -520.950^*^ | 51.964 | 0.000 | -697.419 | -344.481 |
|  |  | PNM-only | -529.695^*^ | 51.320 | 0.000 | -703.979 | -355.411 |
|  |  | SIMPLE + PNM | -460.857^*^ | 54.622 | 0.000 | -646.354 | -275.361 |
|  |  | ICA + PNM | 142.360 | 45.609 | 0.098 | -12.527 | 297.246 |
|  |  | SIMPLE + ICA | 192.823^*^ | 42.776 | 0.003 | 47.556 | 338.089 |
|  |  | Phase-Randomised | -529.695^*^ | 51.320 | 0.000 | -703.979 | -355.411 |
|  | SIMPLE + ICA | SIMPLE | -713.773^*^ | 67.470 | 0.000 | -942.899 | -484.646 |
|  |  | PNM-only | -722.518^*^ | 67.321 | 0.000 | -951.141 | -493.895 |
|  |  | SIMPLE + PNM | -653.680^*^ | 68.768 | 0.000 | -887.214 | -420.146 |
|  |  | ICA + PNM | -50.463 | 52.095 | 1.000 | -227.376 | 126.450 |
|  |  | ICA-only | -192.823^*^ | 42.776 | 0.003 | -338.089 | -47.556 |
|  |  | Phase-Randomised | -722.518^*^ | 67.321 | 0.000 | -951.141 | -493.895 |
|  | Phase-Randomised | SIMPLE | 8.745^*^ | 2.068 | 0.006 | 1.722 | 15.768 |
|  |  | PNM-only | 0.000 | 0.000 |  | 0.000 | 0.000 |
|  |  | SIMPLE + PNM | 68.838^*^ | 8.634 | 0.000 | 39.518 | 98.158 |
|  |  | ICA + PNM | 672.055^*^ | 57.139 | 0.000 | 478.012 | 866.097 |
|  |  | ICA-only | 529.695^*^ | 51.320 | 0.000 | 355.411 | 703.979 |
|  |  | SIMPLE + ICA | 722.518^*^ | 67.321 | 0.000 | 493.895 | 951.141 |
| aDMN | SIMPLE | PNM-only | 321.032^*^ | 39.017 | 0.000 | 188.531 | 453.534 |
|  |  | SIMPLE + PNM | 381.890^*^ | 44.064 | 0.000 | 232.249 | 531.531 |
|  |  | ICA + PNM | 408.167^*^ | 47.456 | 0.000 | 247.007 | 569.328 |
|  |  | ICA-only | 408.167^*^ | 47.456 | 0.000 | 247.007 | 569.328 |
|  |  | SIMPLE + ICA | 408.167^*^ | 47.456 | 0.000 | 247.007 | 569.328 |
|  |  | Phase-Randomised | 408.167^*^ | 47.456 | 0.000 | 247.007 | 569.328 |
|  | PNM-only | SIMPLE | -321.032^*^ | 39.017 | 0.000 | -453.534 | -188.531 |
|  |  | SIMPLE + PNM | 60.858^*^ | 9.364 | 0.000 | 29.057 | 92.659 |
|  |  | ICA + PNM | 87.135^*^ | 12.992 | 0.000 | 43.014 | 131.256 |
|  |  | ICA-only | 87.135^*^ | 12.992 | 0.000 | 43.014 | 131.256 |
|  |  | SIMPLE + ICA | 87.135^*^ | 12.992 | 0.000 | 43.014 | 131.256 |
|  |  | Phase-Randomised | 87.135^*^ | 12.992 | 0.000 | 43.014 | 131.256 |
|  | SIMPLE + PNM | SIMPLE | -381.890^*^ | 44.064 | 0.000 | -531.531 | -232.249 |
|  |  | PNM-only | -60.858^*^ | 9.364 | 0.000 | -92.659 | -29.057 |
|  |  | ICA + PNM | 26.277^*^ | 4.672 | 0.000 | 10.410 | 42.144 |
|  |  | ICA-only | 26.277^*^ | 4.672 | 0.000 | 10.410 | 42.144 |
|  |  | SIMPLE + ICA | 26.277^*^ | 4.672 | 0.000 | 10.410 | 42.144 |
|  |  | Phase-Randomised | 26.277^*^ | 4.672 | 0.000 | 10.410 | 42.144 |
|  | ICA + PNM | SIMPLE | -408.167^*^ | 47.456 | 0.000 | -569.328 | -247.007 |
|  |  | PNM-only | -87.135^*^ | 12.992 | 0.000 | -131.256 | -43.014 |
|  |  | SIMPLE + PNM | -26.277^*^ | 4.672 | 0.000 | -42.144 | -10.410 |
|  |  | ICA-only | 0.000 | 0.000 |  | 0.000 | 0.000 |
|  |  | SIMPLE + ICA | 0.000 | 0.000 |  | 0.000 | 0.000 |
|  |  | Phase-Randomised | 0.000 | 0.000 |  | 0.000 | 0.000 |
|  | ICA-only | SIMPLE | -408.167^*^ | 47.456 | 0.000 | -569.328 | -247.007 |
|  |  | PNM-only | -87.135^*^ | 12.992 | 0.000 | -131.256 | -43.014 |
|  |  | SIMPLE + PNM | -26.277^*^ | 4.672 | 0.000 | -42.144 | -10.410 |
|  |  | ICA + PNM | 0.000 | 0.000 |  | 0.000 | 0.000 |
|  |  | SIMPLE + ICA | 0.000 | 0.000 |  | 0.000 | 0.000 |
|  |  | Phase-Randomised | 0.000 | 0.000 |  | 0.000 | 0.000 |
|  | SIMPLE + ICA | SIMPLE | -408.167^*^ | 47.456 | 0.000 | -569.328 | -247.007 |
|  |  | PNM-only | -87.135^*^ | 12.992 | 0.000 | -131.256 | -43.014 |
|  |  | SIMPLE + PNM | -26.277^*^ | 4.672 | 0.000 | -42.144 | -10.410 |
|  |  | ICA + PNM | 0.000 | 0.000 |  | 0.000 | 0.000 |
|  |  | ICA-only | 0.000 | 0.000 |  | 0.000 | 0.000 |
|  |  | Phase-Randomised | 0.000 | 0.000 |  | 0.000 | 0.000 |
|  | Phase-Randomised | SIMPLE | -408.167^*^ | 47.456 | 0.000 | -569.328 | -247.007 |
|  |  | PNM-only | -87.135^*^ | 12.992 | 0.000 | -131.256 | -43.014 |
|  |  | SIMPLE + PNM | -26.277^*^ | 4.672 | 0.000 | -42.144 | -10.410 |
|  |  | ICA + PNM | 0.000 | 0.000 |  | 0.000 | 0.000 |
|  |  | ICA-only | 0.000 | 0.000 |  | 0.000 | 0.000 |
|  |  | SIMPLE + ICA | 0.000 | 0.000 |  | 0.000 | 0.000 |
| Based on estimated marginal means | | | | | | | |
| *. The mean difference is significant at the .05 level. | | | | | | | |
| b. Adjustment for multiple comparisons: Bonferroni. | | | | | | | |

**Supplementary Table 3F.)** Post-hoc paired *t*-tests between all pipelines for each RSN separately, for connectivity changes after drug administration between RSNs and the rest of the brain

# Supplementary Figure 1: Whole-brain Node Connectivity - Top one percent of changes


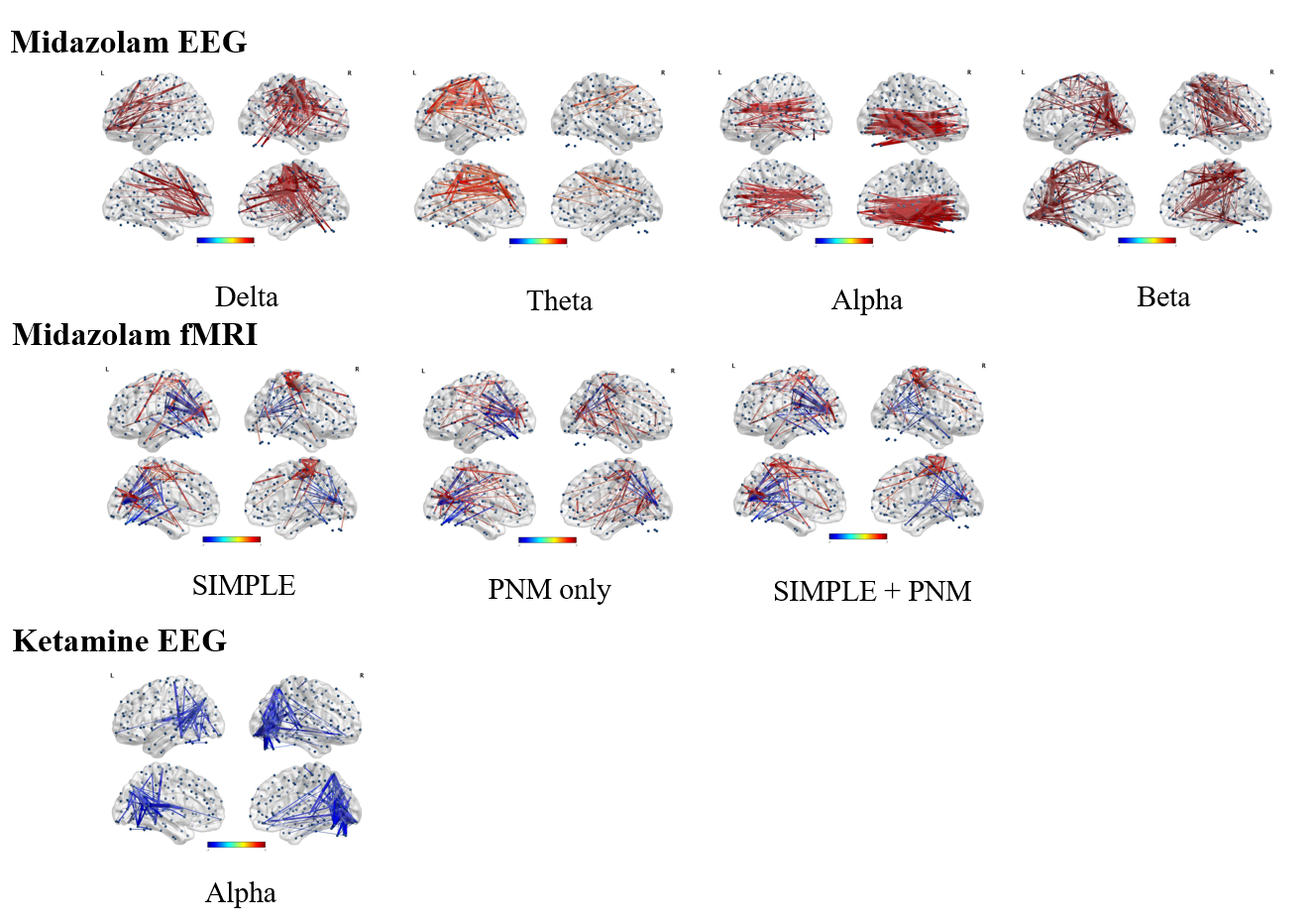


*Supplementary Figure One:* Top one percent of pharmacologically-modulated connectivity changes, for those results that had whole-brain changes, to assess the underlying spatial structure.

# Supplementary Figure 2: Whole-brain Node Connectivity - Placebo changes


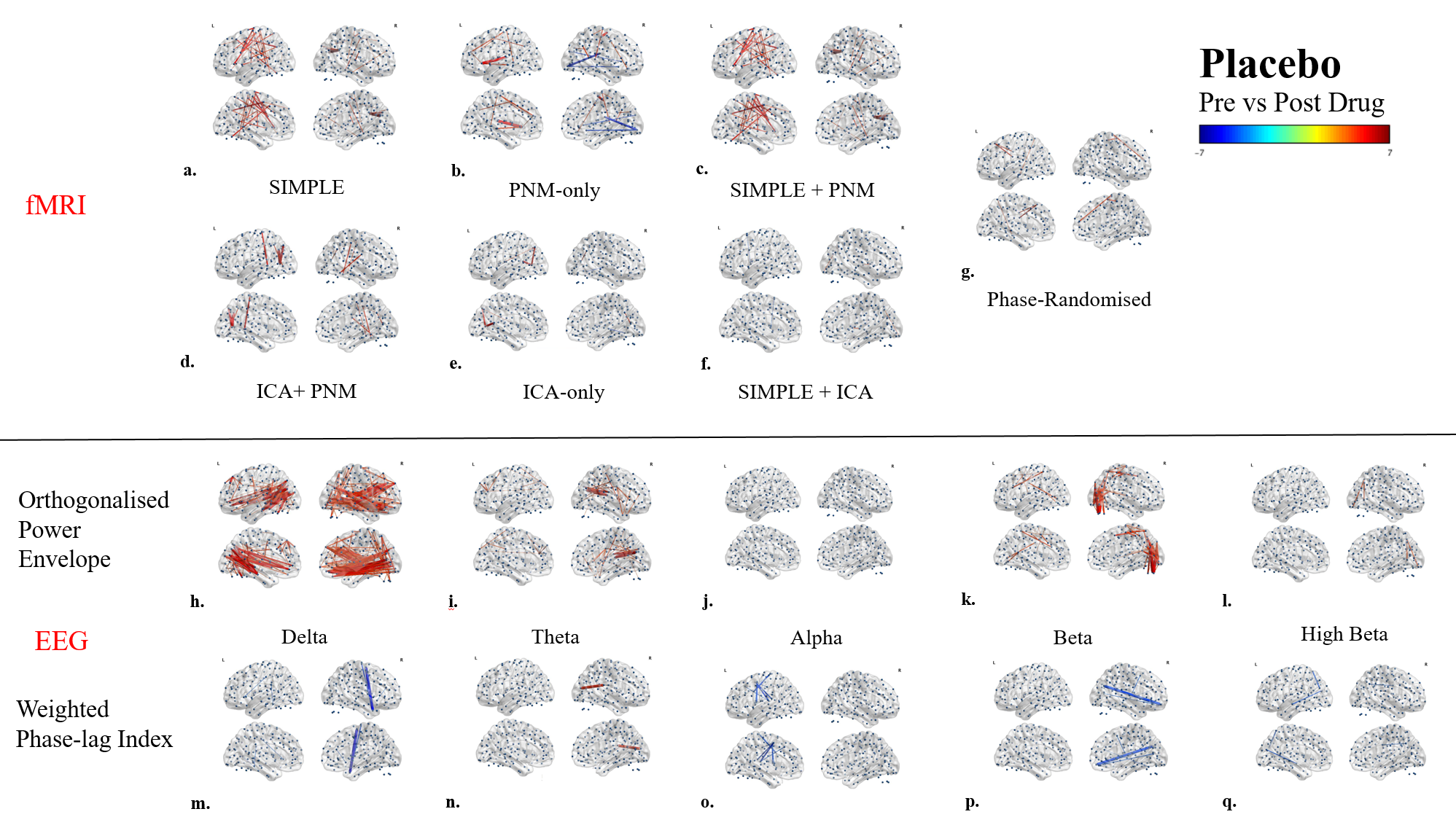


*Supplementary Figure Two:* ***Modulation of all-to-all connectivity of 264 functional nodes after placebo administration.*** *Depicted are results from a FDR corrected (p < .05) one-sample t-test on the difference between post- and pre-drug connectivity estimates, increases depicted in red-yellow, decreases in dark-light blue. Connectivity values are derived through Pearson correlation of the BOLD signal for each pre-processing pipeline (a-g), and via either Pearson correlation of the orthogonalised power envelope (h-l), or the weighted phase-lag index (m-q) for each EEG band.*

# Supplementary Figure 3: Node Connectivity - Changes to pDMN


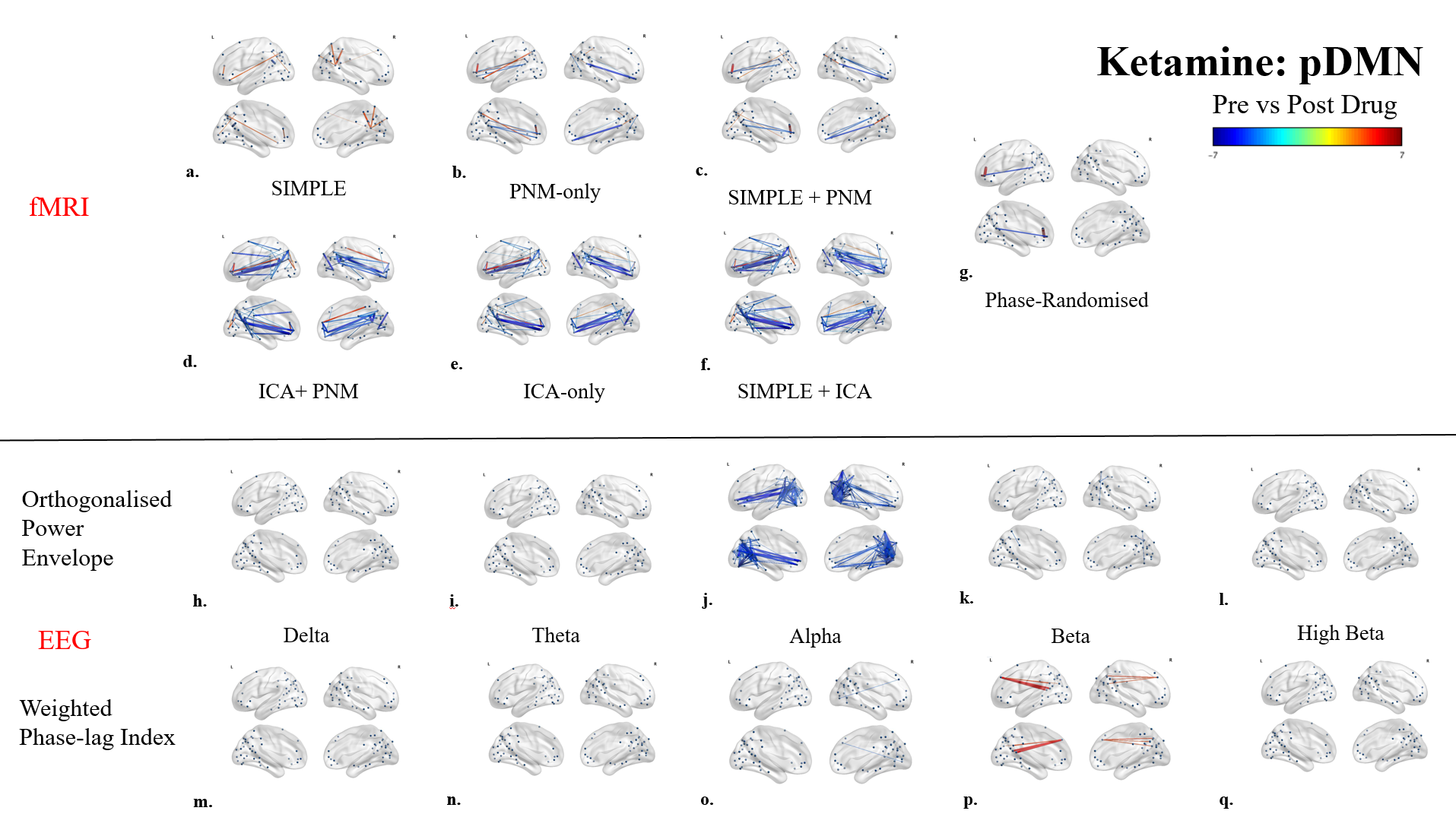


*Supplementary Figure Three:* ***Modulation of all-to-all connectivity of nodes within the pDMN component (derived from ICA in part one of the manuscript), after ketamine administration.*** *Depicted are results from a FDR corrected (p < .05) one-sample t-test on the difference between post- and pre-drug connectivity estimates, increases depicted in red-yellow, decreases in dark-light blue. Connectivity values are derived through Pearson correlation of the BOLD signal for each pre-processing pipeline (a-g), and via either Pearson correlation of the orthogonalised power envelope (h-l), or the weighted phase-lag index (m-q) for each EEG band.*

# Supplementary Figure 4: Node Connectivity - Changes to SMN


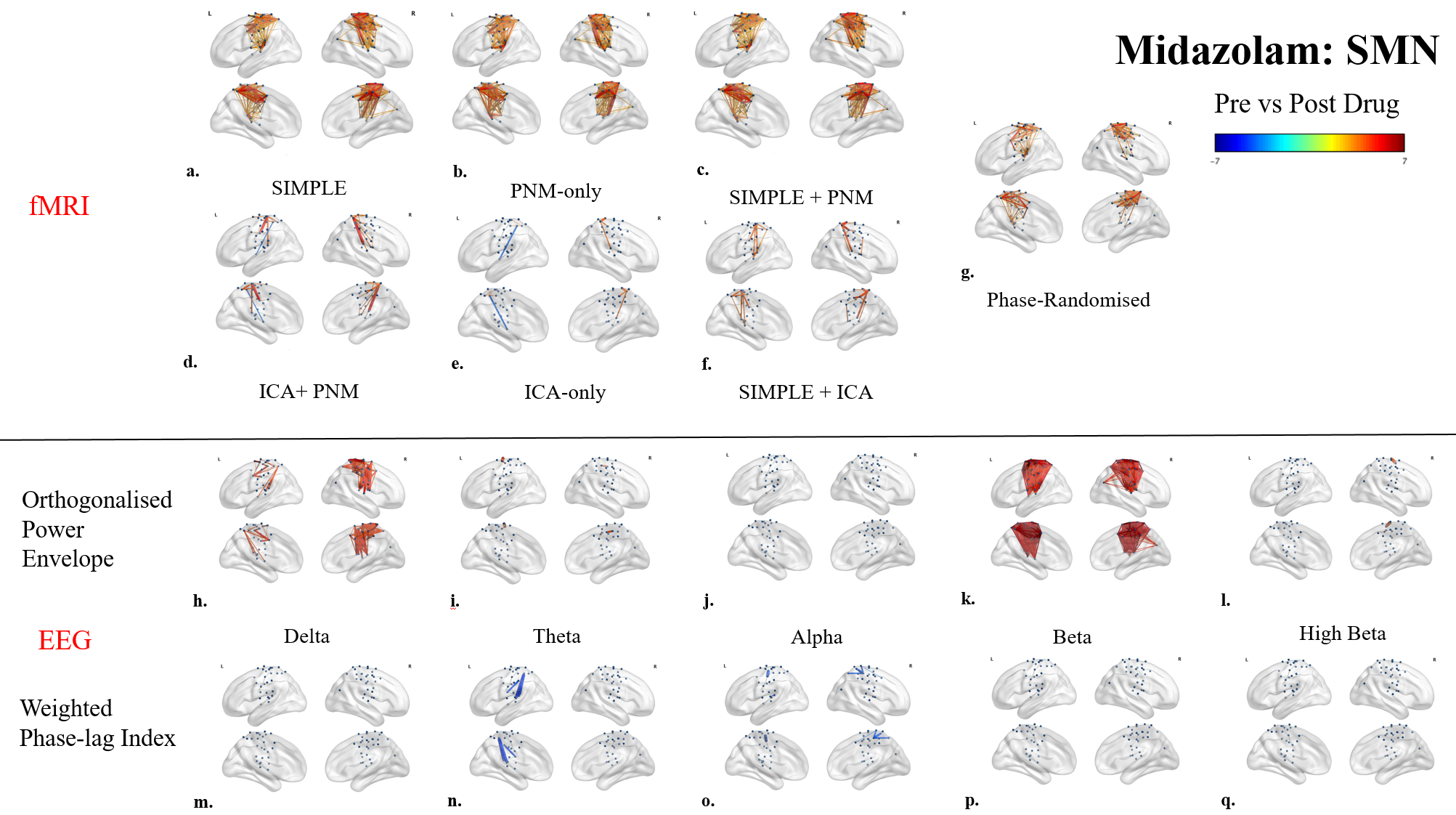


*Supplementary Figure Four:* ***Modulation of all-to-all connectivity of nodes within the SMN component (derived from ICA in part one of the manuscript), after midazolam administration.*** *Depicted are results from a FDR corrected (p < .05) one-sample t-test on the difference between post- and pre-drug connectivity estimates, increases depicted in red-yellow, decreases in dark-light blue. Connectivity values are derived through Pearson correlation of the BOLD signal for each pre-processing pipeline (a-g), and via either Pearson correlation of the orthogonalised power envelope (h-l), or the weighted phase-lag index (m-q) for each EEG band.*

# Supplementary Table 4: Strongest ten changes in BOLD whole-brain node connectivity after Ketamine

**Ketamine: fMRI**

1. **SIMPLE**

|  | Node 1 |  |  | Node 2 |  |  |
| --- | --- | --- | --- | --- | --- | --- |
| Power  Node # | MNI Coordinates  x y z | Region | Power  Node # | MNI Coordinates  x y z | Region | Inc. or Dec. |
| 95  103  100  100  88  100  216  209  95  60 | 11 -54 17  -10 55 39  -35 20 51  -35 20 51  -7 -55 27  -35 20 51  5 23 37  36 22 3  11 -54 17  36 10 1 | Precuneus  Frontal Pole  Mid. Front Gy.  Mid. Front Gy.  Precuneus  Mid Front. Gy.  Paracing. Gy.  Insular Ctx.  Precuneus  Insular Ctx. | 48  79  60  148  60  55  95  91  60  97 | 54 -28 34  -46 -61 21  36 10 1  20 -66 2  36 10 1  -45 0 9  11 -54 17  -3 -49 13  36 10 1  23 33 48 | Supramarg. Gy.  Lat. Occ. Ctx.  Insular Ctx.  Intracalc. Ctx.  Insular Ctx.  Central Operc. Ctx.  Precuneus  Post. Cing. Gy.  Insular Ctx.  Sup. Front. Gy. | ↑  ↓  ↑  ↑  ↑  ↑  ↑  ↑  ↑  ↑ |

1. **PNM ONLY**

|  | Node 1 |  |  | Node 2 |  |  |
| --- | --- | --- | --- | --- | --- | --- |
| Power  Node # | MNI Coordinates  x y z | Region | Power  Node # | MNI Coordinates  x y z | Region | Inc. or Dec. |
| 177  96  95  88  216  103  188  19  96  216 | -53 -49 43  52 -59 36  11 -54 17  -7 -55 27  5 23 37  -10 55 39  -42 38 21  13 -33 75  52 -59 36  5 23 37 | Supramarg. Gy.  Lat. Occ. Ctx.  Precuneus  Precuneus  Paracing. Gy.  Frontal Pole  Frontal Pole  Postcentral Gy.  Lat. Occ. Ctx.  Paracing. Gy. | 124  56  48  76  86  79  116  204  60  209 | -26 -40 -8  49 8 -1  54 -28 34  8 48 -15  -44 -65 35  -46 -61 21  65 -12 -19  55 -45 37  36 10 1  36 22 3 | Lingual Gy.  Cent. Operc. Ctx.  Supramarg. Gy.  Front. Med. Ctx.  Lat. Occ. Ctx.  Lat. Occ. Ctx.  Mid. Temp. Gy.  Supramarg. Gy.  Insular Ctx.  Insular Ctx. | ↑  ↑  ↑  ↓  ↑  ↓  ↑  ↑  ↑  ↓ |

1. **SIMPLE + PNM**

|  | Node 1 |  |  | Node 2 |  |  |
| --- | --- | --- | --- | --- | --- | --- |
| Power  Node # | MNI Coordinates  x y z | Region | Power  Node # | MNI Coordinates  x y z | Region | Inc. or Dec. |
| 177  79  48  88  96  100  132  198  38  56 | -53 -49 43  -46 -61 21  54 -28 34  -7 -55 27  52 -59 36  -35 20 51  -31 19 -19  -42 45 -2  -16 -46 73  49 8 -1 | Supramarg. Gy.  Lat. Occ. Ctx.  Supramarg. Gy.  Precuneus  Lat. Occ. Ctx.  Mid. Front. Gy.  Front. Orb. Ctx.  Frontal Pole  Postcentral Gy.  Cent. Operc. Ctx. | 124  103  95  76  56  58  180  113  204  92 | -26 -40 -8  -10 55 39  11 -54 17  8 48 -15  49 8 -1  -51 8 -2  24 45 -15  -3 42 16  55 -45 37  8 -48 31 | Lingual Gy.  Frontal Pole  Precuneus  Front. Med. Ctx.  Cent. Operc. Ctx.  Cent. Operc. Ctx.  Frontal Pole  Ant. Cing. Gy.  Supramarg. Gy.  Post. Cing. Gy. | ↑  ↓  ↑  ↓  ↑  ↑  ↑  ↑  ↑  ↑ |

1. **ICA + PNM**

|  | Node 1 |  |  | Node 2 |  |  |
| --- | --- | --- | --- | --- | --- | --- |
| Power  Node # | MNI Coordinates  x y z | Region | Power  Node # | MNI Coordinates  x y z | Region | Inc. or Dec. |
| 189  189  109  109  88  103  216  180  209  188 | 38 43 15  38 43 15  -3 44 -9  -3 44 -9  -7 -55 27  -10 55 39  5 23 37  24 45 -15  36 22 3  -42 38 21 | Frontal Pole  Frontal Pole  Paracing. Gy.  Paracing. Gy.  Precuneus  R. Frontal Pole  Paracing. Gy.  L. Frontal Pole  Insular Ctx.  Frontal Pole | 109  98  48  90  190  189  209  189  105  88 | -3 44 -9  -10 39 52  54 -28 34  -11 -56 16  49 -42 45  38 43 15  36 22 3  38 43 15  6 54 16  -7 -55 27 | Paracing. Gy.  Sup. Front. Gy.  Supramarg.Gy.  Precuneus  Supramarg. Gy.  L. Frontal Pole  Insular Ctx.  L. Frontal Pole  Paracing. Gy.  Precuneus | ↑  ↑  ↑  ↓  ↑  ↑  ↓  ↓  ↑  ↑ |

1. **ICA ONLY**

|  | Node 1 |  |  | Node 2 |  |  |
| --- | --- | --- | --- | --- | --- | --- |
| Power  Node # | MNI Coordinates  x y z | Region | Power  Node # | MNI Coordinates  x y z | Region | Inc. or Dec |
| 129  96  109  31  150  54  209  215  189  23 | -53 3 -27  52 -59 36  -3 44 -9  10 -17 74  27 -59 -9  7 8 51  36 22 3  0 30 27  38 43 15  -23 -30 72 | R. Temp. Pole  Lat. Occ. Ctx.  Paracing. Gy.  Precentral Gy.  Temp. Occ. Fusi.  SMA  Insular Ctx.  Ant. Cing. Gy.  L. Frontal Pole  Postcentral Gy. | 128  56  90  21  47  60  216  216  103  34 | 52 7 -30  49 8 -1  -11 -56 16  29 -17 71  -3 2 53  36 10 1  5 23 37  5 23 37  -10 55 39  -21 -31 61 | L. Temp. Pole  Cent. Oper. Ctx.  Precuneus  Precentral Gy.  SMA  Insular Ctx.  Paracing. Gy.  Paracing. Gy.  R. Frontal Pole  Postcentral Gy. | ↓  ↑  ↓  ↓  ↓  ↓  ↓  ↓  ↑  ↓ |

1. **SIMPLE + ICA**

|  | Node 1 |  |  | Node 2 |  |  |
| --- | --- | --- | --- | --- | --- | --- |
| Power  Node # | MNI Coordinates  x y z | Region | Power  Node # | MNI Coordinates  x y z | Region | Inc. or Dec. |
| 209  90  213  109  96  170  43  109  31  91 | 36 22 3  -11 -56 16  -1 15 44  -3 44 -9  52 -59 36  6 -81 6  36 -9 14  -3 44 -9  10 -17 74  -3 -49 13 | Insular Ctx.  Precuneus  Paracing. Gy.  Paracing. Gy.  Lat. Occ. Ctx.  Intracalc. Ctx.  Insular Ctx.  Paracing. Gy.  Precentral Gy.  Post. Cing. Gy. | 79  95  146  90  60  213  24  95  21  110 | -46 -61 21  11 -54 17  -8 -81 7  -11 -56 16  36 10 1  -1 15 44  -40 -19 54  11 -54 17  29 -17 71  8 42 -5 | Lat. Occ. Ctx.  Precuneus  Intracalc. Ctx.  Precuneus  Insular Ctx.  Paracing. Gy.  Precentral Gy.  Precuneus  Precentral Gy.  Paracing. Gy. | ↑  ↓  ↓  ↓  ↑  ↓  ↓  ↓  ↓  ↓ |

1. **PHASE-RANDOMISED**

|  | Node 1 |  |  | Node 2 |  |  |
| --- | --- | --- | --- | --- | --- | --- |
| Power  Node # | MNI Coordinates  x y z | Region | Power  Node # | MNI Coordinates  x y z | Region | Inc. or Dec. |
| 100  113  189  100  100  100  100  198  88  216 | -35 20 51  -3 42 16  38 43 15  -35 20 51  -35 20 51  -35 20 51  -35 20 51  -42 45 -2  -7 -55 27  5 23 37 | Mid. Front. Gy.  Ant. Cing. Gy.  Frontal Pole  Mid. Front. Gy.  Mid. Front. Gy.  Mid. Front. Gy.  Mid. Front. Gy.  Frontal Pole  Precuneus  Paracing. Gy. | 60  198  95  208  56  58  52  107  60  95 | 36 10 1  -42 45 -2  11 -54 17  -35 20 0  49 8 -1  -51 8 -2  37 1 -4  -7 51 -1  36 10 1  11 -54 17 | Insular Ctx.  Frontal Pole  Precuneus  Insular Ctx.  Cent. Operc. Ctx.  Cent. Operc. Ctx.  Insular Ctx.  Paracing. Gy.  Insular Ctx.  Precuneus | ↑  ↑  ↑  ↑  ↑  ↑  ↑  ↑  ↑  ↑ |

***Supplementary Tables 4a-g:*** The ten connections with the strongest changes in connectivity from the BOLD signal after Ketamine administration, for each pre-processing pipeline. Displayed are the node numbers from the database of 264 nodes (Power et al., 2011), their MNI coordinates, and their most probabilistic anatomical region derived from the Harvard-Oxford Cortical and Subcortical Atlases using FSLeyes (<https://zenodo.org/record/2770825>).

# Supplementary Table 5: Strongest ten changes in orthogonalised power envelope EEG whole-brain node connectivity after Ketamine

1. **Delta**

No significant changes in connectivity

1. **Theta**

No significant changes in connectivity

1. **Alpha**

|  | Node 1 |  |  | Node 2 |  |  |
| --- | --- | --- | --- | --- | --- | --- |
| Power  Node  # | MNI Coordinates  x y z | Region | Power  Node # | MNI Coordinates  x y z | Region | Inc.  or  Dec. |
| 223  183  161  161  166  260  148  143  144  245 | -2 -13 12  -18 -76 -24  42 -66 -8  42 -66 -8  -16 -77 34  -27 -71 37  20 -66 2  18 -47 -10  40 -72 14  22 -58 -23 | L. Thalamus  Cerebellum  Lat. Occ. Ctx.  Lat. Occ. Ctx.  Cuneal Ctx.  Lat. Occ. Ctx.  Intracalc. Ctx.  Lingual Gy.  Lat. Occ. Ctx.  Cerebellum | 91  144  146  142  143  143  146  90  141  170 | -3 -49 13  40 -72 14  -8 -81 7  -12 -95 -13  18 -47 -10  18 -47 -10  -8 -81 7  -11 -56 16  17 -91 -14  6 -81 6 | Post. Cing. Gy.  Lat. Occ. Ctx.  Intracalc. Ctx.  Occ. Pole  Lingual Gy.  Lingual Gy.  Intracalc. Ctx.  Precuneus  Occ. Pole  Intracalc. Ctx. | ↓  ↓  ↓  ↓  ↓  ↓  ↓  ↓  ↓  ↓ |

1. **Beta**

|  | Node 1 |  | |  | Node 2 |  |  |
| --- | --- | --- | --- | --- | --- | --- | --- |
| Power  Node  # | MNI Coordinates  x y z | | Region | Power  Node # | MNI Coordinates  x y z | Region | Inc.  or  Dec. |
| 35  258  32  23  31  30  34  263  39  39 | -13 -17 75  25 -58 60  22 -42 69  -23 -30 72  10 -17 74  -29 -43 61  -21 -31 61  -17 -59 64  2 -28 60  2 -28 60 | | Precentral Gy.  Lat. Occ. Ctx.  Postcentral Gy.  Postcentral Gy.  Precentral Gy.  Postcentral Gy.  Precentral Gy.  Lat. Occ. Ctx.  Precentral Gy.  Precentral Gy. | 27  30  30  13  19  27  27  23  25  32 | -38 -27 69  -29 -43 61  -29 -43 61  -7 -52 61  13 -33 75  -38 -27 69  -38 -27 69  -23 -30 72  29 -39 59  22 -42 69 | Postcentral Gy.  Postcentral Gy.  Postcentral Gy.  Precuenus  Postcentral Gy.  Postcentral Gy.  Postcentral Gy.  Postcentral Gy.  Postcentral Gy.  Postcentral Gy. | ↓  ↓  ↓  ↓  ↓  ↓  ↓  ↓  ↓  ↓ |

1. **Beta High**

No significant changes in connectivity

***Supplementary Tables 5a-g:*** The ten connections with the strongest changes in connectivity of the orthogonalized power envelope of each EEG band after Ketamine administration. Displayed are the node numbers from the database of 264 nodes (Power et al., 2011), their MNI coordinates, and their most probabilistic anatomical region derived from the Harvard-Oxford Cortical and Subcortical Atlases using FSLeyes (<https://zenodo.org/record/2770825>).

# Supplementary Table 6: Strongest ten changes in WPLI EEG whole-brain node connectivity after Ketamine

1. **Delta**

|  | Node 1 |  |  | Node 2 |  |  |
| --- | --- | --- | --- | --- | --- | --- |
| Power  Node  # | MNI Coordinates  x y z | Region | Power  Node # | MNI Coordinates  x y z | Region | Inc.  or  Dec. |
| 73 | -30 -27 12 | Insular Ctx. | 50 | -16 -5 71 | Sup. Front. Gy. | ↑ |

1. **Theta**

|  | Node 1 |  |  | Node 2 |  |  |
| --- | --- | --- | --- | --- | --- | --- |
| Power  Node  # | MNI Coordinates  x y z | Region | Power  Node # | MNI Coordinates  x y z | Region | Inc.  or  Dec. |
| 224  229  228  185  232  220  220  197  223  185 | -10 -18 7  31 -14 2  -15 4 8  35 -67 -34  -31 -11 0  -39 51 17  -39 51 17  -34 55 4  -2 -13 12  35 -67 -34 | L. Thalamus  R. Putamen  L. Caudate  Cerebellum  L. Putamen  Frontal Pole  Frontal Pole  Frontal Pole  L. Thalamus  Cerebellum | 185  220  185  57  185  67  43  72  185  73 | 35 -67 -34  -39 51 17  35 -67 -34  -34 3 4  35 -67 -34  43 -23 20  36 -9 14  59 -17 29  35 -67 -34  -30 -27 12 | Cerebellum  Frontal Pole  Cerebellum  Insular Ctx.  Cerebellum  Par. Operc. Ctx.  Insular Ctx.  Postcentral Gy.  Cerebellum  Insular Ctx. | ↑  ↑  ↑  ↑  ↑  ↑  ↑  ↑  ↑  ↑ |

1. **Alpha**

|  | Node 1 |  |  | Node 2 |  |  |
| --- | --- | --- | --- | --- | --- | --- |
| Power  Node  # | MNI Coordinates  x y z | Region | Power  Node # | MNI Coordinates  x y z | Region | Inc.  or  Dec. |
| 119  86  43  200  86  195  181  254  259  177 | 65 -31 -9  -44 -65 35  36 -9 14  43 49 -2  -44 -65 35  -42 -55 45  34 54 -13  46 -47 -17  -33 -46 47  -53 -49 43 | Mid. Temp. Gy.  Lat. Occ. Ctx.  Insular Ctx.  Frontal Pole  Lat. Occ. Ctx.  Angular Gy.  Frontal Pole  Temp. Occ. Fusi.  Supramarg. Gy.  Supramarg. Gy. | 86  9  4  153  11  9  153  144  11  9 | -44 -65 35  65 -24 -19  -56 -45 -24  43 -78 -12  55 -31 -17  65 -24 -19  43 -78 -12  40 -72 14  55 -31 -17  65 -24 -19 | Lat. Occ. Ctx.  Mid. Temp. Gy.  Inf. Temp. Gy.  Lat. Occ. Ctx.  Inf. Temp. Gy.  Mid. Temp. Gy.  Lat. Occ. Ctx.  Lat. Occ. Ctx.  Inf. Temp. Gy.  Mid. Temp. Gy. | ↓  ↓  ↓  ↓  ↓  ↓  ↓  ↓  ↓  ↓ |

1. **Beta**

|  | Node 1 |  |  | Node 2 |  |  |
| --- | --- | --- | --- | --- | --- | --- |
| Power  Node  # | MNI Coordinates  x y z | Region | Power  Node # | MNI Coordinates  x y z | Region | Inc.  or  Dec. |
| 104  104  104  187  163  159  103  145  135  104 | -20 45 39  -20 45 39  -20 45 39  -41 6 33  6 -72 24  15 -77 31  -10 55 39  8 -72 11  11 -66 42  -20 45 39 | Frontal Pole  Frontal Pole  Frontal Pole  Mid. Front. Gy.  Cuneal Ctx.  Cuneal Ctx.  Frontal Pole  Incalc. Ctx.  Precuneus  Frontal Pole | 90  95  93  161  104  104  90  104  99  88 | -11 -56 16  11 -54 17  15 -63 26  42 -66 -8  -20 45 39  -20 45 39  -11 -56 16  -20 45 39  -16 29 53  -7 -55 27 | Precuneus  Precuneus  Precuneus  Lat. Occ. Ctx.  Frontal Pole  Frontal Pole  Precuneus  Frontal Pole  Sup. Front. Gy.  Precuneus | ↑  ↑  ↑  ↑  ↑  ↑  ↑  ↑  ↑  ↑ |

1. **Beta High**

|  | Node 1 |  |  | Node 2 |  |  |
| --- | --- | --- | --- | --- | --- | --- |
| Power  Node  # | MNI Coordinates  x y z | Region | Power  Node # | MNI Coordinates  x y z | Region | Inc.  or  Dec. |
| 260 | -27 -71 37 | Lat Occ. Ctx. | 50 | -16 -5 71 | Sup. Front. Gy. | ↑ |

***Supplementary Tables 6a-g:*** The ten connections with the strongest changes in phase-based connectivity in each EEG band after Ketamine administration. Displayed are the node numbers from the database of 264 nodes (Power et al., 2011), their MNI coordinates, and their most probabilistic anatomical region derived from the Harvard-Oxford Cortical and Subcortical Atlases using FSLeyes (<https://zenodo.org/record/2770825>).

# Supplementary Table 7: Strongest ten changes in BOLD whole-brain node connectivity after Midazolam

1. **SIMPLE**

|  | Node 1 |  |  | Node 2 |  |  |
| --- | --- | --- | --- | --- | --- | --- |
| Power  Node # | MNI Coordinates  x y z | Region | Power  Node # | MNI Coordinates  x y z | Region | Inc. or Dec. |
| 146  19  143  41  167  22  146  30  15  170 | -8 -81 7  13 -33 75  18 -47 -10  38 -17 45  -3 -81 21  10 -46 73  -8 -81 7  -29 -43 61  0 -15 47  6 -81 6 | Intracalc. Ctx.  Postcentral Gy.  Lingual Gy.  Precentral Gy.  Cuneal Ctx  Postcentral Gy.  Intracalc. Ctx.  Postcentral Gy.  Post. Cing. Gy.  Intracalc. Ctx. | 20  18  90  25  90  14  69  146  41  20 | -54 -23 43  -7 -33 72  -11 -56 16  29 -39 59  -11 -56 16  -14 -18 40  -53 -22 23  -8 -81 7  38 -17 45  -54 -23 43 | Postcentral Gy.  Postcentral Gy.  Precuneus  Postcentral Gy.  Precuneus  Precentral Gy.  Cent. Operc. Ctx.  Intracalc. Ctx.  Precentral Gy.  Postcentral Gy. | ↓  ↑  ↑  ↑  ↑  ↑  ↓  ↓  ↑  ↓ |

1. **PNM ONLY**

|  | Node 1 |  |  | Node 2 |  |  |
| --- | --- | --- | --- | --- | --- | --- |
| Power  Node # | MNI Coordinates  x y z | Region | Power  Node # | MNI Coordinates  x y z | Region | Inc. or Dec. |
| 143  25  183  163  167  146  146  149  146  170 | 18 -47 -10  29 -39 59  -18 -76 -24  6 -72 24  -3 -81 21  -8 -81 7  -8 -81 7  -24 -91 19  -8 -81 7  6 -81 6 | Lingual Gy.  Postcentral Gy.  Occ. Fusi. Gy.  Cuneal Ctx.  Cuneal Ctx.  Intracalc. Ctx.  Intracalc. Ctx.  Occ. Pole  Intracalc. Ctx.  Intracalc. Ctx. | 90  146  63  90  90  30  48  90  20  30 | -11 -56 16  -8 -81 7  58 -16 7  -11 -56 16  -11 -56 16  -29 -43 61  54 -28 34  -11 -56 16  -54 -23 43  -29 -43 61 | Precuneus  Intracalc Ctx.  Planum Temp.  Precuneus  Precuneus  Postcentral Gy.  Supramarg. Gy.  Precuneus  Postcentral Gy.  Postcentral Gy. | ↑  ↓  ↓  ↑  ↑  ↓  ↓  ↑  ↓  ↓ |

1. **SIMPLE + PNM**

|  | Node 1 |  |  | Node 2 |  |  |
| --- | --- | --- | --- | --- | --- | --- |
| Power  Node # | MNI Coordinates  x y z | Region | Power  Node # | MNI Coordinates  x y z | Region | Inc. or Dec. |
| 146  167  19  170  143  146  146  183  41  39 | -8 -81 7  -3 -81 21  13 -33 75  6 -81 6  18 -47 -10  -8 -81 7  -8 -81 7  -18 -76 -24  38 -17 45  2 -28 60 | Intracalc. Ctx.  Cuneal Ctx.  Postcentral Gy.  Intracalc. Ctx.  Lingual Gy.  Intracalc. Ctx.  Intracalc. Ctx.  Occ. Fusi. Gy.  Precentral Gy.  Precentral Gy. | 20  90  18  20  90  69  30  63  25  13 | -54 -23 43  -11 -56 16  -7 -33 72  -54 -23 43  -11 -56 16  -53 -22 23  -29 -43 61  58 -16 7  29 -39 59  -7 -52 61 | Postcentral Gy.  Precuneus  Postcentral Gy.  Postcentral Gy.  Precuneus  Cent. Operc. Ctx.  Postcentral Gy.  Planum Temp.  Postcentral Gy.  Precuneus | ↓  ↑  ↑  ↓  ↑  ↓  ↓  ↓  ↑  ↑ |

1. **ICA + PNM**

|  | Node 1 |  |  | Node 2 |  |  |
| --- | --- | --- | --- | --- | --- | --- |
| Power  Node # | MNI Coordinates  x y z | Region | Power  Node # | MNI Coordinates  x y z | Region | Inc. or Dec. |
| 243  48  210  146  145  170  216  155  109  167 | -16 -65 -20  54 -28 34  37 32 -2  -8 -81 7  8 -72 11  6 -81 6  5 23 37  -14 -91 31  -3 44 -9  -3 -81 21 | Cerebellum  Supramarg. Gy.  Front. Orb. Ctx.  Intracalc. Ctx.  Intracalc. Ctx.  Intracalc. Ctx.  Paracing. Cy.  Occ. Pole  Paracing. Cy.  Cuneal Ctx. | 48  146  110  69  170  48  90  156  59  48 | 54 -28 34  -8 -81 7  8 42 -5  -53 -22 23  6 -81 6  54 -28 34  -11 -56 16  15 -87 37  -5 18 34  54 -28 34 | Supramarg Gy.  Intracalc. Ctx.  Paracing. Gy.  Cent. Operc. Ctx.  Intracalc. Ctx.  Supramarg. Gy.  Precuneus  Lat. Occ. Ctx.  Ant. Cing. Gy.  Supramarg. Gy. | ↓  ↓  ↑  ↓  ↑  ↓  ↑  ↑  ↑  ↓ |

1. **ICA ONLY**

|  | Node 1 |  |  | Node 2 |  |  |
| --- | --- | --- | --- | --- | --- | --- |
| Power  Node # | MNI Coordinates  x y z | Region | Power  Node # | MNI Coordinates  x y z | Region | Inc. or Dec. |
| 216  60  89  155  48  146  109  216  170  97 | 5 23 37  36 10 1  6 -59 35  -14 -91 31  54 -28 34  -8 -81 7  -3 44 -9  5 23 37  6 -81 6  23 33 48 | Paracing. Gy  Insula Ctx.  Precuneus  Occ. Pole  Supramarg. Gy.  Intracalc. Ctx.  Paracing. Gy.  Paracing. Cy.  Intracalc. Ctx.  Sup. Front. Gy. | 87  92  58  160  167  48  59  90  145  162 | -39 -75 44  8 -48 31  -51 8 -2  -16 -52 -1  -3 -81 21  54 -28 34  -5 18 34  -11 -56 16  8 -72 11  24 -87 24 | Lat. Occ. Ctx.  Post. Cing. Gy.  Central Oper. Ctx.  Lingual gy.  Cuneal Ctx.  Supramarg. Gy  Ant. Cing. Gy.  Precuneus  Intracalc Ctx.  Lat. Occ. Ctx. | ↑  ↑  ↑  ↑  ↓  ↓  ↑  ↑  ↑  ↑ |

1. **SIMPLE + ICA**

|  | Node 1 |  |  | Node 2 |  |  |
| --- | --- | --- | --- | --- | --- | --- |
| Power  Node # | MNI Coordinates  x y z | Region | Power  Node # | MNI Coordinates  x y z | Region | Inc. or Dec. |
| 146  48  167  170  221  143  48  155  92  209 | -8 -81 7  54 -28 34  -3 -81 21  6 -81 6  2 -24 30  18 -47 -10  54 -28 34  -14 -91 31  8 -48 31  36 22 3 | Intracalc. Ctx.  Supramarg. Gy.  Cuneal Ctx.  Intracalc. Ctx.  Post. Cing. Gy.  Lingual Gy.  Supramarg. Gy.  Occ. Pole  Post. Cing. Gy.  Insular Ctx. | 48  170  48  254  202  90  156  156  60  76 | 54 -28 34  6 -81 6  54 -28 34  46 -47 -17  -3 26 44  -11 -56 16  15 -87 37  15 -87 37  36 10 1  8 48 -15 | Supramarg. Gy.  Intracalc. Ctx.  Supramarg. Gy.  Temp. Occ. Fus.  Paracing. Gy.  Precuneus  Occ. Pole  Occ. Pole  Insular Ctx.  Frontal Med. Ctx. | ↓  ↓  ↓  ↓  ↑  ↑  ↓  ↑  ↑  ↑ |

1. **PHASE-RANDOMISED**

|  | Node 1 |  |  | Node 2 |  |  |
| --- | --- | --- | --- | --- | --- | --- |
| Power  Node # | MNI Coordinates  x y z | Region | Power  Node # | MNI Coordinates  x y z | Region | Inc. or Dec. |
| 19  40  143  39  32  167  203  99  19  15 | 13 -33 75  3 -17 58  18 -47 -10  2 -28 60  22 -42 69  -3 -81 21  11 -39 50  -16 29 53  13 -33 75  0 -15 47 | Postcentral Gy.  Precentral Gy.  Lingual Gy.  Precentral Gy.  Postcentral Gy.  Cuneal Ctx.  Precuneus  Sup. Front. Gy.  Postcentral Gy.  Post. Cing. Gy. | 18  17  90  13  39  90  17  50  17  13 | -7 -33 72  -7 -21 65  -11 -56 16  -7 -52 61  2 -28 60  -11 -56 16  -7 -21 65  -16 -5 71  -7 -21 65  -7 -52 61 | Postcentral Gy.  Precentral Gy.  Precuneus  Precuneus  Precentral Gy.  Precuneus  Precentral Gy.  Sup. Front. Gy.  Precentral Gy.  Precunueus | ↑  ↑  ↑  ↑  ↑  ↑  ↑  ↑  ↑  ↑ |

***Supplementary Tables 7a-g:*** The ten connections with the strongest changes in connectivity from the BOLD signal after Midazolam administration, for each pre-processing pipeline. Displayed are the node numbers from the database of 264 nodes (Power et al., 2011), their MNI coordinates, and their most probabilistic anatomical region derived from the Harvard-Oxford Cortical and Subcortical Atlases using FSLeyes (<https://zenodo.org/record/2770825>).

# Supplementary Table 8: Strongest ten changes in orthogonalised power envelope EEG whole-brain node connectivity after Midazolam

1. **Delta**

|  | Node 1 |  |  | Node 2 |  |  |
| --- | --- | --- | --- | --- | --- | --- |
| Power  Node # | MNI Coordinates  x y z | Region | Power  Node  # | MNI Coordinates  x y z | Region | Inc.  or  Dec. |
| 61  221  221  223  224  133  224  182  134  215 | 32 -26 13  2 -24 30  2 -24 30  -2 -13 12  -10 -18 7  -2 -35 31  -10 -18 7  -21 41 -20  -7 -71 42  0 30 27 | Insular Ctx  Post. Cing. Gy.  Post. Cing. Gy.  L. Thalamus  L. Thalamus  Post. Cing. Gy.  L. Thalamus  Frontal Pole  Precuneus  Ant. Cing. Gy. | 59  31  217  31  78  31  75  53  105  61 | -5 18 34  10 -17 74  10 22 27  10 -17 74  -18 63 -9  10 -17 74  6 67 -4  13 -1 70  6 54 16  32 -26 13 | Ant. Cing. Gy.  Precentral Gy.  Ant. Cing. Gy.  Precentral Gy.  Frontal Pole  Precentral Gy.  Frontal Pole  Sup. Front. Gy.  Paracing. Gy.  Insular Ctx | ↑  ↑  ↑  ↑  ↑  ↑  ↑  ↑  ↑  ↑ |

1. **Theta**

|  | Node 1 |  |  | Node 2 |  |  |
| --- | --- | --- | --- | --- | --- | --- |
| Power  Node # | MNI Coordinates  x y z | Region | Power  Node # | MNI Coordinates  x y z | Region | Inc.  or  Dec. |
| 201  100  203  201  201  220  202  258  202  188 | -42 25 30  -35 20 51  11 -39 50  -42 25 30  -42 25 30  -39 51 17  -3 26 44  25 -58 60  -3 26 44  -42 38 21 | Mid. Front. Gy.  Mid. Front. Gy.  Precuneus  Mid. Front. Gy.  Mid. Front. Gy.  Frontal Pole  Paracing. Ctx.  Lat. Occ. Ctx.  Paracing. Ctx.  Frontal Pole | 92  39  100  94  133  188  148  99  43  92 | 8 -48 31  2 -28 60  -35 20 51  -2 -37 44  -2 -35 31  -42 38 21  20 -66 2  -16 29 53  36 -9 14  8 -48 31 | Post. Cing. Gy.  Precentral Gy.  Mid. Front. Gy.  Post. Cing. Gy.  Post. Cing. Gy. Frontal Pole  Intracal. Ctx.  Sup. Front. Gy.  Insular Ctx.  Post. Cing. Gy. | ↑  ↑  ↑  ↑  ↑  ↑  ↑  ↑  ↑  ↑ |

1. **Alpha**

|  | Node 1 |  |  | Node 2 | |  |  |
| --- | --- | --- | --- | --- | --- | --- | --- |
| Power  Node # | MNI Coordinates  x y z | Region | Power  Node  # | MNI Coordinates  x y z | Region | | Inc.  or  Dec. |
| 188  188  188  242  188  188  189  208  198  208 | -42 38 21  -42 38 21  -42 38 21  -49 25 -1  -42 38 21  -42 38 21  38 43 15  -35 20 0  -42 45 -2  -35 20 0 | Frontal Pole  Frontal Pole  Frontal Pole  Front. Oper. Ctx.  Frontal Pole  Frontal Pole  Frontal Pole  Insular Ctx.  Frontal Pole  Insular Ctx. | 163  145  167  169  162  144  169  144  157  169 | 6 -72 24  8 -72 11  -3 -81 21  37 -84 13  24 -87 24  40 -72 14  37 -84 13  40 -72 14  29 -77 25  37 -84 13 | Precuneus  Intracalc. Ctx.  Cuneal Ctx.  Lat. Occ. Ctx.  Lat. Occ. Ctx  Lat. Occ. Ctx.  Lat. Occ. Ctx.  Lat. Occ. Ctx.  Lat. Occ. Ctx.  Lat. Occ. Ctx. | | ↑  ↑  ↑  ↑  ↑  ↑  ↑  ↑  ↑  ↑ |

1. **Beta**

|  | Node 1 |  |  | Node 2 |  |  |
| --- | --- | --- | --- | --- | --- | --- |
| Power  Node # | MNI Coordinates  x y z | Region | Power  Node  # | MNI Coordinates  x y z | Region | Inc.  or  Dec. |
| 263  252  64  248  251  263  111  134  251  202 | -17 -59 64  -52 -63 5  -38 -33 17  -31 -10 -36  10 -62 61  -17 -59 64  -11 45 8  -7 -71 42  10 -62 61  -3 26 44 | Lat. Occ. Ctx.  Lat. Occ. Ctx.  Planum Temp.  Temp. Fusi. Ctx.  Lat. Occ. Ctx.  Lat. Occ. Ctx.  Ant. Cing. Gy.  Precuneus  Lat. Occ. Ctx.  Paracing. Gy. | 262  39  1  164  213  252  110  35  216  97 | -42 -60 -9  2 -28 60  -25 -98 -12  -42 -74 0  -1 15 44  -52 -63 5  8 42 -5  -13 -17 75  5 23 37  23 33 48 | Inf. Temp. Gy.  Precentral Gy.  Occ. Pole  Lat. Occ. Ctx.  Paracing. Gy.  Lat Occ. Ctx.  Paracing. Gy.  Precentral Gy.  Paracing. Gy.  Sup. Front. Gy. | ↑  ↑  ↑  ↑  ↑  ↑  ↑  ↑  ↑  ↑ |

1. **Beta High**

|  | Node 1 |  |  | Node 2 |  |  |
| --- | --- | --- | --- | --- | --- | --- |
| Power  Node # | MNI Coordinates  x y z | Region | Power  Node # | MNI Coordinates  x y z | Region | Inc.  or  Dec. |
| 136  219  219  136  122  250  206  122  179  203 | 4 -48 51  26 50 27  26 50 27  4 -48 51  12 36 20  -50 -7 -39  31 33 26  12 36 20  58 -53 -14  11 -39 50 | Precuneus  Frontal Pole  Frontal Pole  Precuneus  Ant. Cing. Gy.  Inf. Temp. Gy.  Mid. Front. Gy.  Ant. Cing. Gy.  Inf. Temp. Gy.  Precuneus | 101  136  94  122  88  121  136  94  38  101 | 22 39 39  4 -48 51  -2 -37 44  12 36 20  -7 -55 27  13 30 59  4 -48 51  -2 -37 44  -16 -46 73  22 39 39 | Frontal Pole  Precuneus  Post. Cing. Gy.  Ant. Cing. Gy. Precuneus  Sup. Front. Gy.  Precuneus  Post. Cing. Gy.  Postcentral Gy.  Frontal Pole | ↑  ↑  ↑  ↑  ↑  ↑  ↑  ↑  ↑  ↑ |

***Supplementary Tables 8a-g:*** The ten connections with the strongest changes in connectivity of the orthogonalized power envelope of each EEG band after Midazolam administration. Displayed are the node numbers from the database of 264 nodes (Power et al., 2011), their MNI coordinates, and their most probabilistic anatomical region derived from the Harvard-Oxford Cortical and Subcortical Atlases using FSLeyes (<https://zenodo.org/record/2770825>).

# Supplementary Table 9: Strongest ten changes in WPLI EEG whole-brain node connectivity after Ketamine

1. **Delta**

|  | Node 1 |  |  | Node 2 |  |  |
| --- | --- | --- | --- | --- | --- | --- |
| Power  Node # | MNI Coordinates  x y z | Region | Power  Node # | MNI Coordinates  x y z | Region | Inc.  or  Dec. |
| 203  121  152  147  164  172  84  84  117  84 | 11 -39 50  13 30 59  -18 -68 5  -28 -79 19  -42 -74 0  -33 -79 -13  -58 -26 -15  -58 -26 -15  -56 -13 -10  -58 -26 -15 | Precuneus  Sup. Front. Gy.  Intracalc. Ctx.  Lat. Occ. Ctx.  Lat. Occ. Ctx.  Occ. Fusi. Gy.  Mid. Temp. Gy.  Mid. Temp. Gy.  Mid. Temp. Gy.  Mid. Temp. Gy. | 96  41  16  16  16  16  5  76  75  78 | 52 -59 36  38 -17 45  10 -2 45  10 -2 45  10 -2 45  10 -2 45  8 41 -24  8 48 -15  6 67 -4  -18 63 -9 | Lat. Occ. Cortex  Precentral Gr.  Ant. Cing. Gy.  Ant. Cing. Gy.  Ant. Cing. Gy.  Ant. Cing. Gy.  Front. Med. Ctx  Front. Med. Ctx  Frontal Pole  Frontal Pole | ↑  ↑  ↓  ↓  ↓  ↓  ↓  ↓  ↓  ↓ |

1. **Theta**

|  | Node 1 |  |  | Node 2 |  |  |
| --- | --- | --- | --- | --- | --- | --- |
| Power  Node  # | MNI Coordinates  x y z | Region | Power  Node # | MNI Coordinates  x y z | Region | Inc.  or  Dec. |
| 182  182  106  42  108  190  110  76  190  137 | -21 41 -20  -21 41 -20  6 64 22  -49 -11 35  9 54 3  49 -42 45  8 42 -5  8 48 -15  49 -42 45  -46 31 -13 | Frontal Pole  Frontal Pole  Frontal Pole  Precentral Gy.  Paracing. Gy.  Supramarg. Gy.  Paracing. Gy.  Front. Med. Ctx.  Supramarg. Gy.  Front. Orb. Ctx. | 44  26  33  6  33  182  33  33  8  44 | 51 -6 32  50 -20 42  -45 -32 47  -21 -22 -20  -45 -32 47  -21 41 -20  -45 -32 47  -45 -32 47  -37 -29 -26  51 -6 32 | Precentral Gy.  Postcentral Gy.  Postcentral Gy.  Parahipp. Gy.  Postcentral Gy.  Frontal Pole  Postcentral Gy.  Postcentral Gy.  Temp. Fusi. Ctx.  Precentral Gy. | ↓  ↓  ↓  ↓  ↓  ↓  ↓  ↓  ↓  ↓ |

1. **Alpha**

|  | Node 1 |  |  | Node 2 |  |  |
| --- | --- | --- | --- | --- | --- | --- |
| Power  Node # | MNI Coordinates  x y z | Region | Power  Node # | MNI Coordinates  x y z | Region | Inc.  or  Dec. |
| 225  223  226  234  81  222  263  233  245  263 | 12 -17 8  -2 -13 12  -5 -28 -4  9 -4 6  -44 12 -34  6 -24 0  -17 -59 64  15 5 7  22 -58 -23  -17 -59 64 | R. Thalamus  L. Thalamus  Brain Stem  R. Thalamus  Temporal Pole  R. Thalamus  Lat. Occ. Ctx.  R. Pallidum  Cerebellum  Lat. Occ. Cortex | 81  81  81  81  61  81  32  81  177  25 | -44 12 -34  -44 12 -34  -44 12 -34  -44 12 -34  32 -26 13  -44 12 -34  22 -42 69  -44 12 -34  -53 -49 43  29 -39 59 | Temporal Pole  Temporal Pole  Temporal Pole  Temporal Pole  Insular Cortex  Temporal Pole  Postcentral Gy.  Temporal Pole  Supramarg. Gy.  Postcentral Gy. | ↓  ↓  ↓  ↓  ↓  ↓  ↓  ↓  ↓  ↓ |

1. **Beta**

|  | Node 1 |  |  | Node 2 |  |  |
| --- | --- | --- | --- | --- | --- | --- |
| Power  Node  # | MNI Coordinates  x y z | Region | Power  Node # | MNI Coordinates  x y z | Region | Inc.  or  Dec. |
| 189  189  205  230  229  218  152  142  152  217 | 38 43 15  38 43 15  42 0 47  23 10 1  31 -14 2  31 56 14  -18 -68 5  -12 -95 -13  -18 -68 5  10 22 27 | Frontal Pole  Frontal Pole  Precentral Gy.  R. Putamen  R. Putamen  Frontal Pole  Intracalc. Ctx.  Occ. Pole  Intracalc. Ctx.  Ant. Cing. Gy. | 7  125  129  209  207  7  47  51  112  206 | 17 -28 -17  27 -37 -13  -53 3 -27  36 22 3  48 22 10  17 -28 -17  -3 2 53  -10 -2 42  -2 38 36  31 33 26 | Parahipp. Gy.  Parahipp Gy.  Mid. Temp. Gy.  Insular Ctx.  Inf. Front. Gy.  Parahipp. Gy.  SMA  SMA  Paracing. Gy.  Mid. Front. Gy. | ↓  ↓  ↓  ↓  ↓  ↓  ↓  ↓  ↓  ↓ |

1. **Beta-High**

|  | Node 1 |  |  | Node 2 |  |  |
| --- | --- | --- | --- | --- | --- | --- |
| Power  Node  # | MNI Coordinates  x y z | Region | Power  Node # | MNI Coordinates  x y z | Region | Inc.  or  Dec. |
| 260  156  146  156  260  260  170  171  260  98 | -27 -71 37  15 -87 37  -8 -81 7  15 -87 37  -27 -71 37  -27 -71 37  6 -81 6  -26 -90 3  -27 -71 37  -10 39 52 | Lat. Occ. Ctx.  Occ. Pole  Intracalc. Ctx.  Occ. Pole  Lat. Occ. Ctx.  Lat. Occ. Ctx.  Intracalc. Ctx.  Lat. Occ. Ctx.  Lat. Occ. Ctx.  Sup. Front. Gy. | 95  82  125  52  89  93  1  145  92  86 | 11 -54 17  46 16 -30  27 -37 -13  37 1 -4  6 -59 35  15 -63 26  -25 -98 -12  8 -72 11  8 -48 31  -44 -65 35 | Precuneus  Temporal Pole  Parahipp. Gy.  Insular Ctx.  Precuneus  Precuneus  Occ. Pole  Intracalc. Ctx.  Post. Cing. Gy. Lat. Occ. Ctx. | ↓  ↓  ↓  ↓  ↓  ↓  ↓  ↓  ↓  ↓ |

***Supplementary Tables 9a-g:*** The ten connections with the strongest changes in phase-based connectivity in each EEG band after Midazolam administration. Displayed are the node numbers from the database of 264 nodes (Power et al., 2011), their MNI coordinates, and their most probabilistic anatomical region derived from the Harvard-Oxford Cortical and Subcortical Atlases using FSLeyes (<https://zenodo.org/record/2770825>).

# Supplementary Figure 5: Spatial distribution of variance across time for the ketamine session


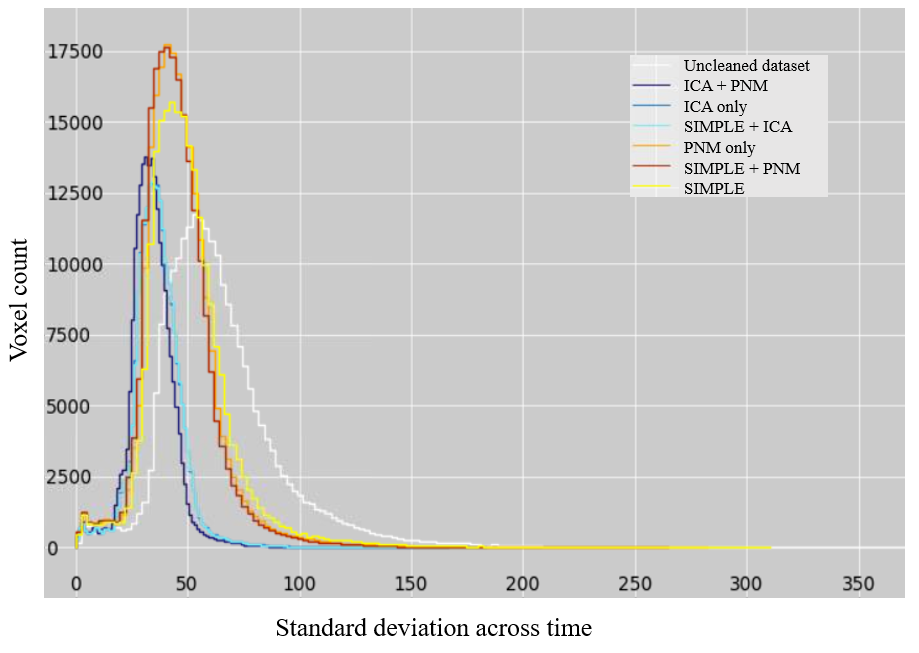


***Supplementary Figure 5:*** *Histogram of average variance (in standard deviation across time) for each voxel during the post-drug section of the ketamine session, for the uncleaned dataset, and the remaining variance after cleaning with the different pre-processing pipelines.*

# Supplementary Figure 6: Spatial distribution of variance across time for the midazolam session


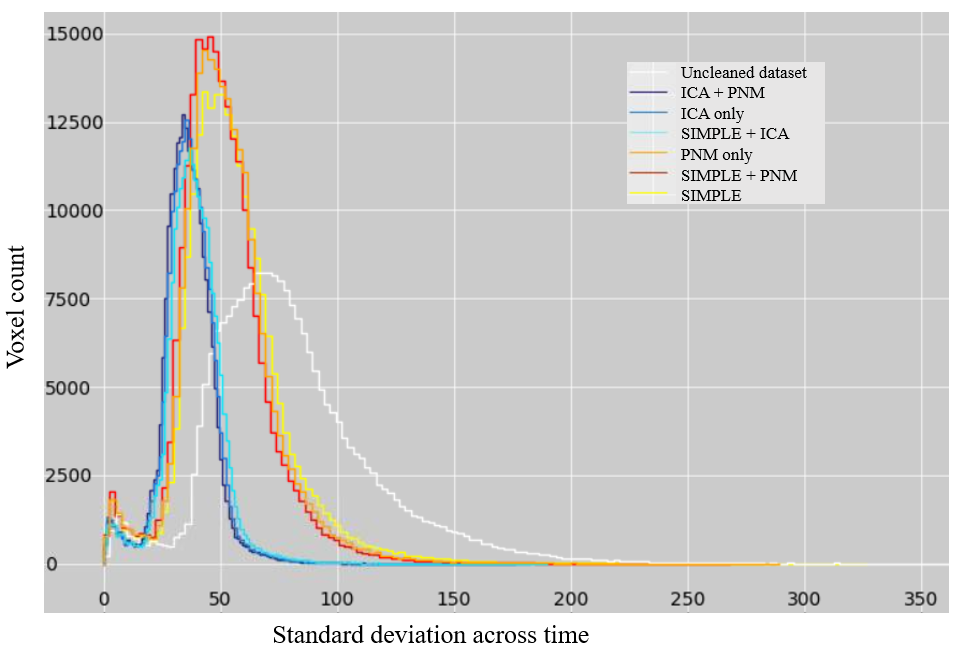


***Supplementary Figure 6:*** *Histogram of average variance (in standard deviation across time) for each voxel during the post-drug section of the midazolam session, for the uncleaned dataset, and the remaining variance after cleaning with the different pre-processing pipelines.*

# Supplementary Table 10: Differences in average variance across time between pre-processing pipelines

| **Pipeline** | **Ketamine** | **Midazolam** |
| --- | --- | --- |
| Uncleaned | 67.13 (1.99) | 85.96 (5.56) |
| SIMPLE without motion regressors | 56.94 (1.25) | 66.58 (3.89) |
| SIMPLE | 52.43 (0.90) | 58.96 (2.39) |
| PNM-only | 48.57 (0.92) | 55.96 (2.66) |
| SIMPLE + PNM | 47.34 (0.86) | 53.78 (2.27) |
| ICA-only | 37.7 (0.37) | 38.17 (0.60) |
| SIMPLE + ICA | 38.69 (0.37) | 39.91 (0.70) |
| ICA + PNM | 34.96 (0.37) | 37.26 (0.69) |

***Supplementary Table 10:*** Average variance across time, for the uncleaned ketamine and midazolam post-drug datasets, and the average variance remaining after cleaning with various pre-processing pipelines. Standard errors are presented in brackets.

# Supplementary Figure 7: Spatial maps of highest 75% temporal variance


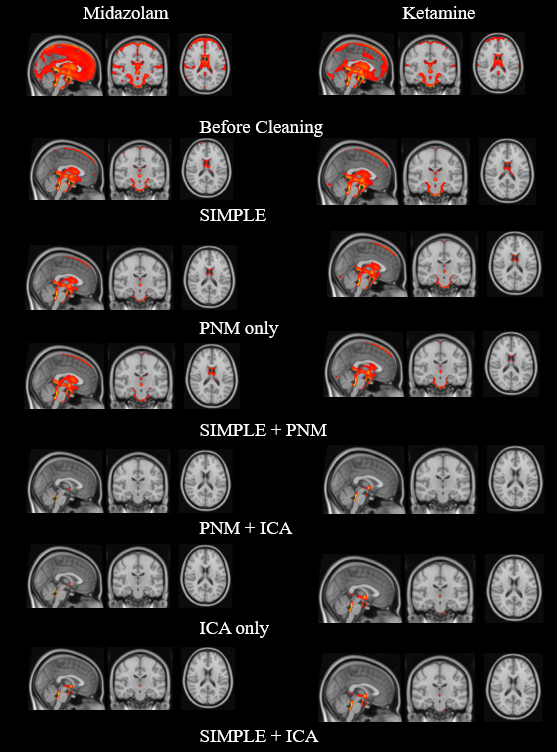


***Supplementary Figure 7:*** *Spatial maps of the 75% of voxels with the highest average standard deviation across time, before cleaning, and after cleaning with various pre-processing pipelines*
